# Hydrophilic BIPHEPHOS Ligand for Pd-Mediated Cysteine Allylation of Peptides and Proteins in Water

**DOI:** 10.1021/acs.orglett.5c04112

**Published:** 2025-11-11

**Authors:** Thomas Schlatzer, Julia Kriegesmann, Mark Bieber, Christian F. W. Becker, Rolf Breinbauer

**Affiliations:** † Institute of Organic Chemistry, 27253 Graz University of Technology, Stremayrgasse 9, A-8010 Graz, Austria; ‡ Institute of Biological Chemistry, Faculty of Chemistry, University of Vienna, Währinger Strasse 38, A-1090 Vienna, Austria

## Abstract

The selective and natural post-translational modification of peptides and proteins under biocompatible conditions remains a challenge. Herein, we report a hydrophilic variant of the BIPHEPHOS ligand featuring diethyl phosphonates as solubilizing groups, which were found to be catalytically isofunctional to unpolar *tert*-butyl. This enabled the fast introduction of native prenyl groups and bioconjugation handles into peptides and folded proteins under nearly aqueous conditions.

## Graphical abstract

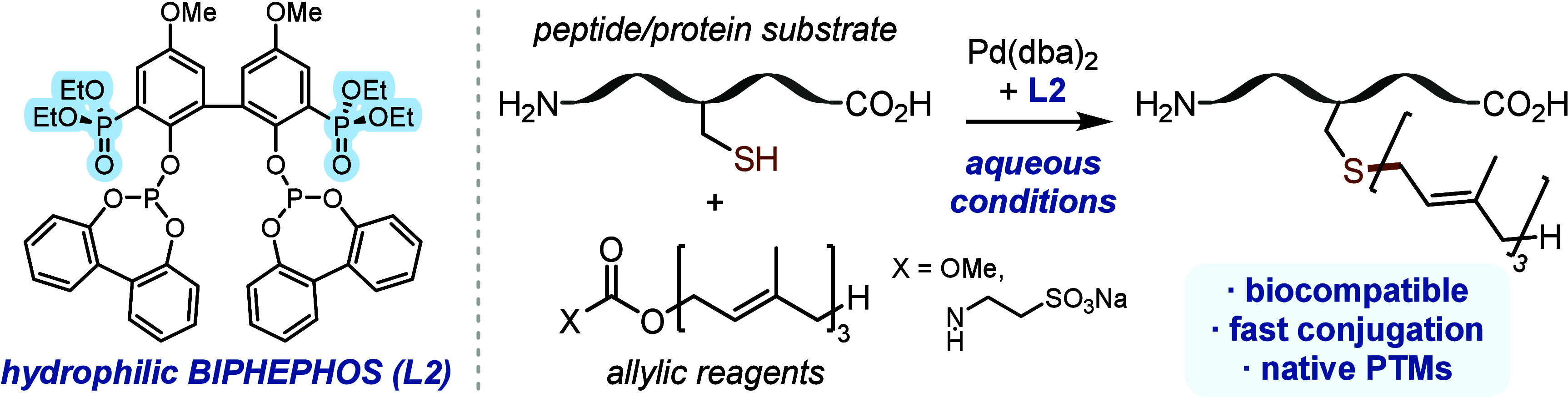

Post-translational modifications (PTMs) are important for controlling protein function and localization in cells.^1^ In order to investigate the influence of different PTMs on proteins and their networks in biophysical or cell biological experiments, it is important to obtain the desired homogeneously modified protein variants in sufficiently large amounts. This goal can be achieved by a variety of methods that were developed to introduce different modifications onto amino acid side chains.^2^ While several strategies enable the chemoselective introduction of such modifications into peptides and proteins,^3^ some challenges remain. These are related to the facts that in many cases non-native linkages are generated when introducing PTMs and that reaction conditions are often not biocompatible, thus disrupting protein folding.^2c^ Despite the plethora of available bioconjugation reactions, methods that are chemoselective, lead to a native PTM, and work under physiological conditions remain scarce.

We have previously reported a Pd-mediated *S*-allylation protocol for the chemoselective introduction of native prenylations into peptides and proteins.^4^ Prenylations are often used by cells to link intracellular proteins to membranes.^5^ Using this allylation protocol, we were able to chemoselectively farnesylate and geranylgeranylate peptides and proteins such as ubiquitin-like protein 3 (UBL3). Furthermore, we could attach fluorophores, affinity tags, or bioconjugation handles to cysteines within peptides and proteins. However, the catalyst system of Pd­(dba)_2_ with BIPHEPHOS as ligand depends on at least 50 vol % acetonitrile (ACN) in water as solvent to ensure sufficient solubility for efficient modification. These reaction conditions can lead to protein denaturation, which then requires an additional folding step, potentially impacting the overall yield and applicability of this allylation strategy. In order to overcome this limitation, we embarked on developing a water-soluble catalyst system based on a hydrophilic ligand for the Pd-catalyst.

The introduction of hydrophilic groups (anionic, cationic, or neutral) still represents the most reliable approach to increase the aqueous solubility of ligands and thereby their respective metal complexes.^6^ The hydrolytic instability of the phosphite groups of BIPHEPHOS (**L1**) excluded conventional sulfonation chemistry as well as late-stage cleavage of sulfonate esters.^7^ We thus envisioned replacing some substituents of the bridging biphenol of BIPHEPHOS (**L1**) with more polar groups, being synthetically more accessible than the flanking biphenols and preserving the modularity of the ligand assembly. At the outset, the 5,5′-positions seemed most advantageous from both a synthetic and catalytic point of view, since the structural editing of the ligand would be located as distant as possible from the coordinating motif (i.e., bisphosphite core). Unfortunately, all our efforts to exchange the 5,5′-substituents by groups that are larger than methoxy resulted in the formation of unsymmetric ligands (Figure 1). Ring-interconversion during the synthesis of biphenol bisphosphites has been previously reported; however, its origin remains poorly understood.^8^

**Figure 1:**
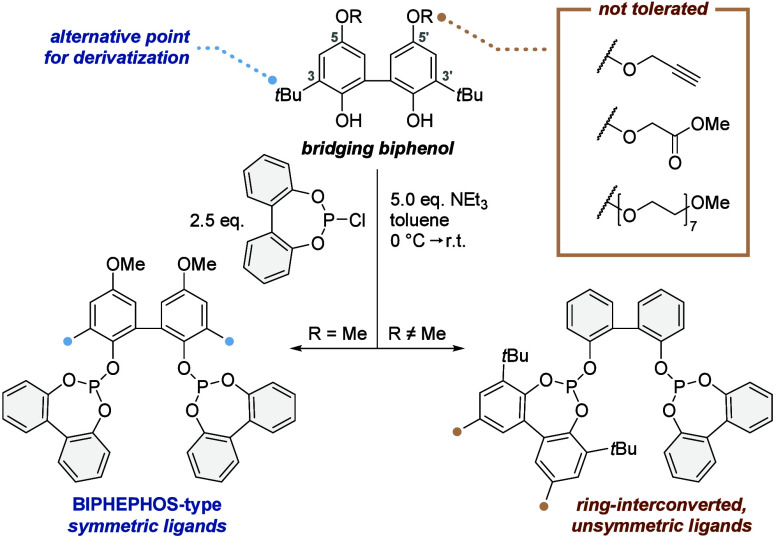
Structural constraints during the ligand assembly step lead to symmetric or ring-interconverted ligands.

We therefore turned our attention to the *tert*-butyl groups located at the 3,3′-positions of the bridging biphenol. Given the spatial proximity of the 3,3′-substituents to the metal center of Pd-allyl complexes,^9^ a possible relation between the steric bulk of these groups and the regioselectivity of the catalyst needed to be taken into consideration. To this end, we hypothesized that phosphonate esters might introduce enough polarity to serve as hydrophilic groups^10^ and yet resemble the steric demand of the *tert*-butyl groups. The formal substitution of the most hydrophobic group (i.e., *t*Bu) by the hydrophilic moiety was expected to have an even higher impact on the aqueous solubility of the ligand when compared to conventional strategies.^6^ In contrast to other hydrophilic groups, *ortho*-phosphonylated phenols are readily accessible (cf. phospho-Fries rearrangement^11^).

Starting from 2,2′-biphenol (**1**), dibromination followed by a Cu-catalyzed methanolysis of the intermediate aryl bromide **2** furnished the 3,3′-unsubstituted biphenol **3** in a good overall yield. This central intermediate in the synthesis of other BIPHEPHOS-analogues can be produced on a gram-scale due to the easy scalability of this two-step protocol, thus circumventing reproducibility issues with literature procedures.^12^ Subsequent Atherton–Todd reaction with diethylphosphonate afforded the bisphosphate **4**, which could be subjected to an O → C migration upon *ortho*-metalation with LDA. The resulting bisphosphonate **5** was then treated with chlorophosphite **6** to give the desired, symmetric bisphosphite ligand **L2** (Figure 2). The formal substitution of *tert*-butyl by diethylphosphonate had only a minor effect on the basicity of the phosphite, as evidenced by ^1^ *J*(^77^Se–^31^P) coupling constants^13^ of the corresponding diselenides (1044 Hz for **L2** versus 1037 Hz for BIPHEPHOS). Of note, the structural editing of the ligand did not affect its intrinsic *n*/*i* selectivity as well as reversibility in Pd-catalyzed *S*-allylations (Table S1), in line with our previous study.^4b^

**Figure 2:**
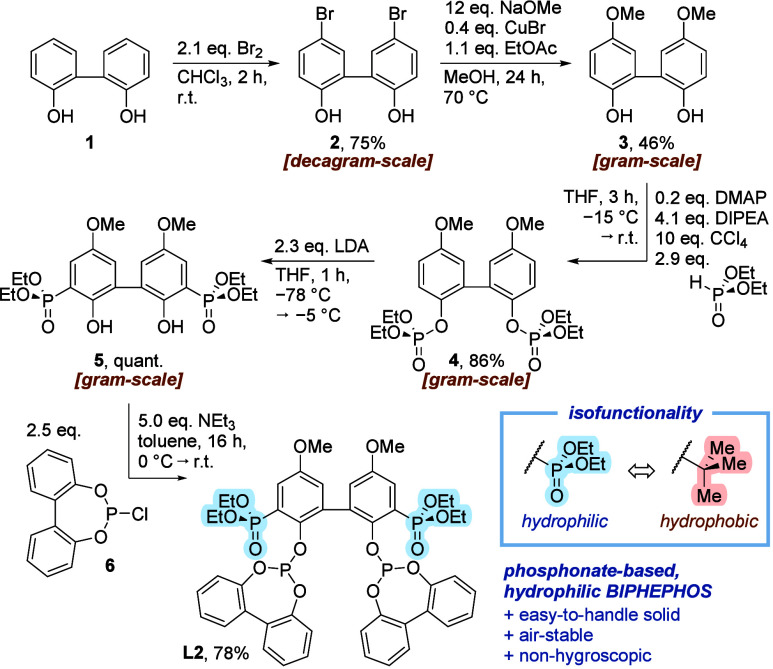
Synthesis of **L2** as a phosphonate-based, hydrophilic variant of BIPHEPHOS highlighting the catalytic isofunctionality between hydrophilic diethyl phosphonate and hydrophobic *tert*-butyl.

Having the hydrophilic ligand **L2** in hand, its performance in the Pd-catalyzed *S*-allylation was assessed and compared against BIPHEPHOS (**L1**) at 2 mol % catalyst loading using glutathione as a test substrate. To our delight, hydrophilic **L2** outperformed BIPHEPHOS (**L1**) under all conditions tested (Figure 3). The difference in catalytic performance (i.e., conversion toward the *S*-allylated product) became increasingly pronounced as the amount of organic cosolvent was reduced. This corroborates an immediate positive effect of the ligand’s hydrophilicity on its catalytic performance and suggested potential further applications in the context of longer peptides and proteins. To the best of our knowledge, this represents the first report of dialkyl phosphonate acting as a catalytically isofunctional group to *tert*-butyl. This strategy might be applicable for the synthesis of hydrophilic derivatives of various other ligands, given the prevalence of *tert*-butyl groups in ligand design.

**Figure 3:**
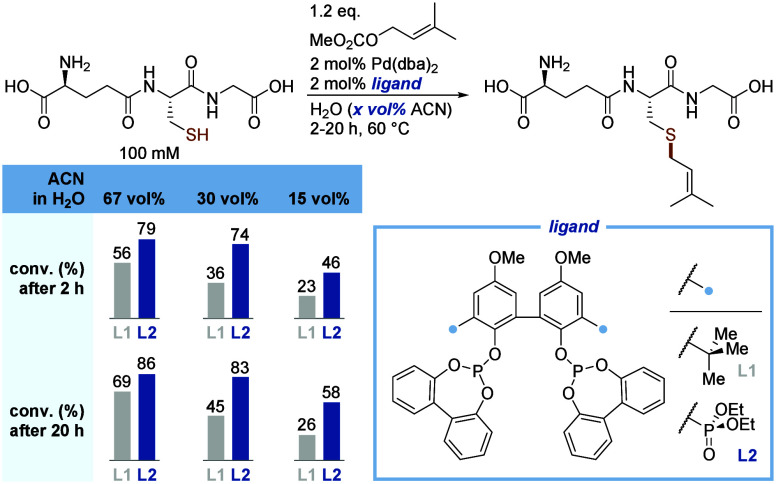
Catalytic performance in aqueous-phase *S*-allylation for **L2** in comparison with that of BIPHEPHOS (**L1**). Conversions were determined by HPLC-MS.

Next, we tested our reaction with the new bisphosphite ligand **L2** on peptide **P1** using 1.2 equiv of [PdL] as well as allylic carbonate **Ra**. A slight excess of Pd complex is required to compensate for nonspecific binding to the peptide substrate as reported previously.^4a^ However, here we use only 5 vol % ACN in water as solvent, an ACN concentration very well tolerated by most proteins.^14^ To our delight, the desired alkyne-modified product **P1a** was obtained with full conversion after only 5 min (Figure 4).

**Figure 4:**
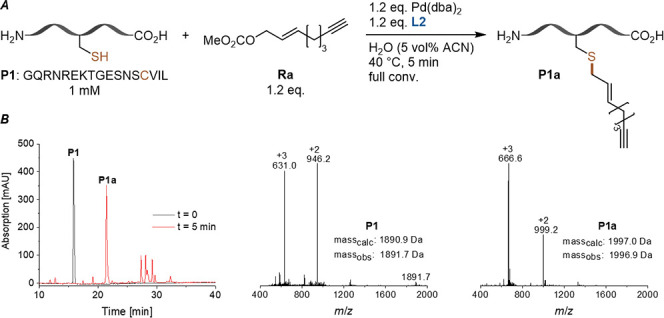
Modification of peptide **P1** with an alkyne functionality under nearly aqueous conditions (5 vol % ACN in H_2_O). *A*: Reaction conditions for cysteine labeling. *B*: HPLC traces (214 nm) of substrate (black) and crude reaction mixture (after 5 min, red) and mass spectra of unmodified and alkyne-modified **P1**.

In a next step, we compared farnesylation reactions of peptide **P1** using Pd­(dba)_2_ with ligands **L1** and **L2** in different solvents. When performing this reaction under previously published reaction conditions using Pd­(dba)_2_/**L1** and farnesyl carbonate **Rb** in 50 vol % ACN, full conversion was achieved in 5 h (Figure 5A).^4a^ With our new ligand **L2**, farnesyl carbonate **Rb**, and reducing the amount of ACN to 15 vol %, ∼80% conversion could be observed in 24 h (Figure S1A). Further decreasing the percentage of ACN to 5 vol % led to ∼50% conversion after 24 h (Figure S1B). These results indicated that additionally to the solubility of the [PdL] species also the solubility of the farnesyl reagent severely impacts conversion. The impact of the latter on peptide and protein modification reactions has been described previously.^15^ To address this issue, we employed a more water-soluble carbamate-based farnesylation reagent, **Rc** (Figure 5B).^16^ When Pd­(dba)_2_/**L2** and **Rc** were applied in 5 vol % ACN in water, formation of the desired product exceeded 50% after only 5 min (Figure S2A,B).

**Figure 5:**
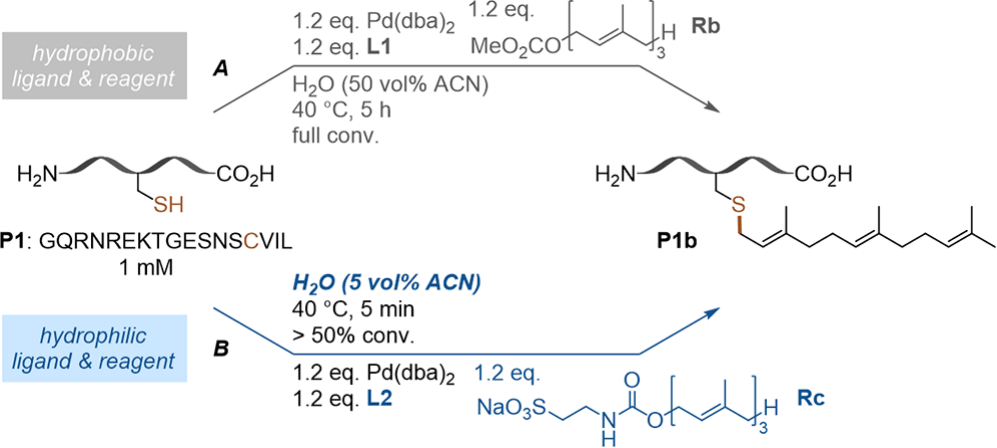
Farnesylation of **P1** under different solvent conditions. *A*: Previously published conditions with Pd­(dba)_2_/**L1** and **Rb** in 50 vol % ACN in H_2_O.^4a^ *B*: New reaction conditions with Pd­(dba)_2_/**L2** and farnesyl carbamate **Rc** in 5 vol % ACN in H_2_O.

Encouraged by these results, we applied the new Pd­(dba)_2_/**L2** system together with the alkyne carbonate reagent **Ra** to two model proteins: ubiquitin-like protein 3 (UBL3)^17^ and heat shock protein 27 (Hsp27).^18^ We dissolved the lyophilized proteins in degassed H_2_O and added the Pd complex dissolved in ACN, leading to a final concentration of 5 vol % ACN. For both proteins, the reaction was as fast as that for peptide **P1** (which is derived from the cysteine-containing *C*-terminus of UBL3), leading to full conversion to the desired products in only 5 min (Figure 6).

**Figure 6:**
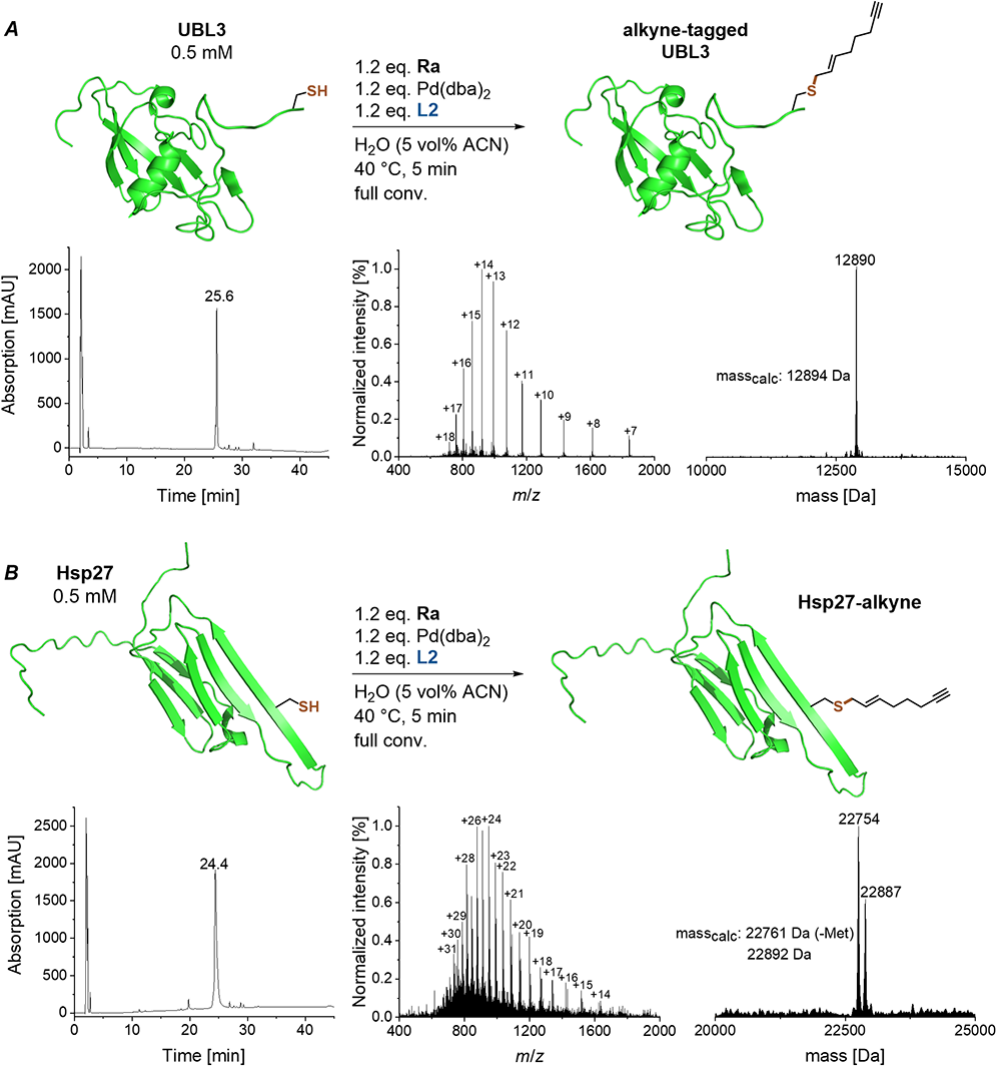
Modification of the model proteins UBL3 (*A*) and Hsp27 (*B*) with an alkyne functionality under nearly aqueous conditions (5 vol % ACN in H_2_O).

To take full advantage of the improved water solubility of ligand **L2** and the complete conversion, we aimed to isolate the modified and folded proteins without HPLC purification. Therefore, we removed all insoluble components formed during the reaction by centrifugation. The resulting supernatant was dialyzed against a degassed 50 mM potassium phosphate buffer (KP_i_) at pH 7 overnight. The protein solutions were analyzed by circular dichroism (CD) spectroscopy, affording very similar spectra as the unmodified, folded proteins. However, we observed a significant shift below 215 nm for all samples compared to the unmodified protein variants that have not seen the Pd complex (Figure 7A). Incubation of unmodified protein with 1.2 equiv of Pd­(dba)_2_ and 1.2 equiv of **L2**, or with 1.2 equiv of **L2** alone in 5 vol % ACN in H_2_O for 5 min at 40 °C led to a similar shift (Figure 7B). Since no such shift was observed in previous protein modification reactions with Pd­(dba)_2_ and the original BIPHEPHOS ligand,^4a^ we concluded that the [PdL] complex interacts more strongly with the protein in the absence of high ACN concentrations and impacts the CD traces. To ensure removal of Pd or ligand **L2** from modified proteins, treatment with chelating agents was investigated. While 10 mM EDTA proved to be ineffective, incubation with 10 mM DTT was able to revert the observed changes to the CD spectra (Figure 7C,D). This behavior was demonstrated for two proteins with different secondary structures, indicating that the addition of DTT is beneficial for [PdL] removal rather than an indication of protein denaturation. To further corroborate that the observed changes are based on interactions with the Pd complex, we carried out ICP-MS measurements to determine the amounts of Pd in a sample of modified UBL3. We found that purification including treatment with DTT reduced the residual Pd content by 96% as compared to the initial Pd loading.

**Figure 7:**
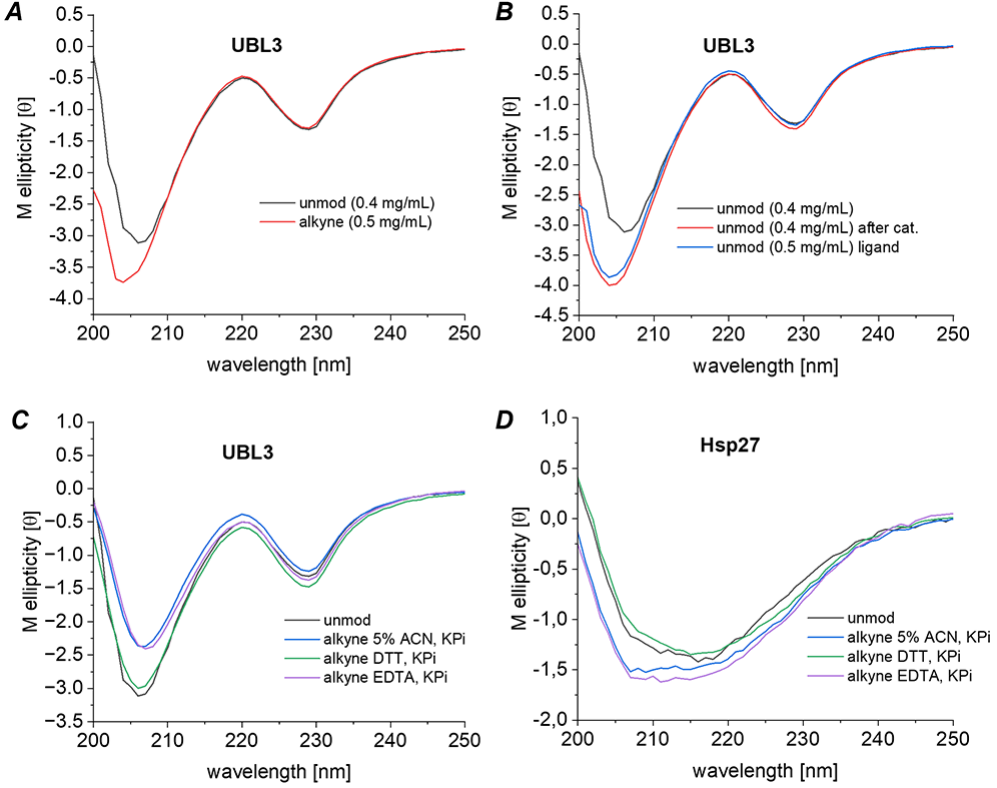
CD measurements of UBL3 and Hsp27. *A*: Comparison of unmodified UBL3 and alkyne-modified UBL3 in KP_i_. *B*: Comparison of unmodified UBL3 and unmodified UBL3 incubated with Pd­(dba)_2_/**L2** and ligand **L2** alone in KP_i_. *C*: Comparison of unmodified UBL3 in KP_i_ (black) and alkyne-modified UBL3 treated differently before dialysis against KP_i_: two-step dialysis against 5 vol % ACN in H_2_O, followed by KP_i_ dialysis (blue), incubation with 10 mM DTT and removal of the pellet by centrifugation (green), incubation with 10 mM EDTA and removal of the pellet by centrifugation (purple). *D*: Comparison of unmodified Hsp27 in KP_i_ (black) and alkyne-modified Hsp27 treated differently before dialysis against KP_i_: two-step dialysis against 5 vol % ACN in H_2_O, followed by KP_i_ dialysis (blue), incubation with 10 mM DTT and removal of the pellet by centrifugation (green), incubation with 10 mM EDTA and removal of the pellet by centrifugation (purple).

In a next step, we also tested the farnesyl carbamate reagent **Rc** with improved water solubility in modification reactions on UBL3 and Hsp27. For UBL3, ∼50% conversion was observed after 5 min (Figure S2C–E), but similar to reactions with peptide **P1**, increasing the reaction time did not lead to more product. In the case of Hsp27, no product was identified and we only found unmodified protein during LC-MS measurements at different time points (up to 24 h). This surprising finding could be explained by the fact that Hsp27 is known to form large oligomers (a crucial part of its chaperone function),^19^ which could lead to a drastically reduced accessibility for larger, hydrophobic reaction partners to the buried cysteine residue, explaining the difference in reactivity between alkyne and farnesyl modification of Hsp27. In UBL3 the cysteine is positioned at the exposed *C*-terminus. Overall, this reflects a potential opportunity for site-selectivity that can be achieved when using chemoselective reactions on folded proteins.

In conclusion, we report a bioorthogonal reaction based on a hydrophilic variant of the bisphosphite ligand BIPHEPHOS for the Pd-mediated *S*-allylation of folded peptides and proteins under aqueous conditions. Its design rests on the replacement of the lipophilic *tert*-butyl group with a hydrophilic dialkyl phosphonate acting as an isofunctional group, a strategy that might be more widely applicable when replacing hydrophobic *tert*-butyl groups prevalent in other ligands. We have applied the new Pd complex to introduce an alkyne handle into a peptide as well as the proteins UBL3 and Hsp27 with only 5 vol % ACN in water within 5 min. In order to extend this approach to native prenylations such as farnesylation, we employed a farnesyl carbamate reagent with increased water solubility that leads to better conversion than the established allyl carbonates. We believe that the very low amount of organic cosolvent required (5 vol % ACN) makes this reaction much more suitable for the modification of folded proteins, eliminating the need for late-stage folding steps potentially linked to low yields. At the same time, we see opportunities for site-selective modifications of folded proteins if only certain cysteine side chains are accessible and reduced.

## Supplementary Material



## Data Availability

The data underlying this study are available in the published article and its Supporting Information.

## Notes

Experimental procedures, characterization data, NMR spectra, chromatography traces, and mass spectra (PDF)

## §

Department of Chemistry, Chemistry Research Laboratory, University of Oxford, Oxford OX1 3TA, United Kingdom

## ⊥

Department of Biochemistry, Faculty of Chemistry and Biochemistry, Ruhr University, Bochum, Germany.
