# Supplementary material for: Hydrophilic BIPHEPHOS Ligand for Pd-Mediated Cysteine Allylation of Peptides and Proteins in Water
Source: Org Lett. 2025 Nov 11;27(46):12813–8. doi: 10.1021/acs.orglett.5c04112 (PMC12645573; doi:10.1021/acs.orglett.5c04112)
Supplement: Supplementary file 1 [file ol5c04112_si_001.pdf]

Supporting Information

Hydrophilic BIPHEPHOS Ligand for Pd-mediated Cysteine Allylation of Peptides and Proteins in Water

Thomas Schlatzer,[a] † Julia Kriegesmann,[b] † Mark Bieber,[a] Christian F. W. Becker*[b] and Rolf Breinbauer*[a]

[a] Institute of Organic Chemistry, Graz University of Technology, Stremayrgasse 9, A-8010 Graz, Austria
[b] Institute of Biological Chemistry, Faculty of Chemistry, University of Vienna, Währinger Strasse 38, A-1090 Vienna, Austria

†T.S. and J.K. contributed equally.

*Correspondence to: christian.becker@univie.ac.at and breinbauer@tugraz.at

Table of Contents

Supplementary Catalytic Data and Figures 4

n/*i* Selectivity 4

Farnesylation of **P1** and UBL3 5

Removal of Catalyst by Dialysis 6

General Information 7

Chemicals 7

Thin Layer Chromatography 8

Flash Column Chromatography 8

Gas Chromatography 8

High Performance Liquid Chromatography 9

Nuclear Magnetic Resonance Spectroscopy 9

High Resolution Mass Spectrometry 10

Determination of Melting Points 10

Experimental Procedures 11

Ligand Synthesis 11

2-(*tert*-Butyl)-4-(prop-2-yn-1-yloxy)phenol (**S1**) 11

3,3'-Di-*tert*-butyl-5,5'-bis(prop-2-yn-1-yloxy)-[1,1'-biphenyl]-2,2'-diol (**S2**) 11

4,8-Di-*tert*-butyl-6-((2'-(dibenzo[*d*,*f*][1,3,2]dioxaphosphepin-6-yloxy)-[1,1'-biphenyl]-2-yl)oxy)-2,10-bis(prop-2-yn-1-yloxy)dibenzo[*d*,*f*][1,3,2]dioxaphosphepine (**S3**) 12

Ethyl 2-(3-(*tert*-butyl)-4-hydroxyphenoxy)acetate (**S4**) 13

2,2'-((5,5'-Di-*tert*-butyl-6,6'-dihydroxy-[1,1'-biphenyl]-3,3'- diyl)bis(oxy))diacetic acid (**S5**) 13

Dimethyl 2,2'-((5,5'-di-*tert*-butyl-6,6'-dihydroxy-[1,1'-biphenyl]- 3,3'-diyl)bis(oxy))diacetate (**S6**) 14

Dimethyl 2,2'-((4,8-di-*tert*-butyl-6-((2'-(dibenzo[*d*,*f*][1,3,2]dioxa-phosphepin-6-yloxy)-[1,1'-biphenyl]-2-yl)oxy)dibenzo[*d*,*f*][1,3,2]- dioxaphosphepin-2,10-diyl)bis(oxy))diacetate (**S7**) 15

5,5'-Dibromo-[1,1'-biphenyl]-2,2'-diol (**2**) 15

5,5'-Dimethoxy-[1,1'-biphenyl]-2,2'-diol (**3**) 16

5,5'-Dimethoxy-[1,1'-biphenyl]-2,2'-diyl tetraethyl bis(phosphate) (**4**) 17

Tetraethyl (2,2'-dihydroxy-5,5'-dimethoxy-[1,1'-biphenyl]-3,3'-diyl)bis(phosphonate) (**5**) 17

6-Chlorodibenzo[*d*,*f*][1,3,2]dioxaphosphepine (**6**) 18

Tetraethyl (2,2'-bis(dibenzo[*d*,*f*][1,3,2]dioxaphosphepin-6-yloxy)-5,5'-dimethoxy-[1,1'-biphenyl]-3,3'-diyl)bis(phosphonate) (**L2**) 19

Synthesis and Characterization of Diselenides 19

Synthesis of Allylic Reagents 21

4-Nitrophenyl ((2*E*,6*E*)-3,7,11-trimethyldodeca-2,6,10-trien-1-yl) carbonate (**S8**) 21

Sodium 2-aminoethane-1-sulfonate (**S9**) 21

Sodium 2-(((((2*E*,6*E*)-3,7,11-trimethyldodeca-2,6,10-trien-1-yl)oxy)carbonyl)amino)ethane-1-sulfonate (**Rc**) **22**

Catalytic Performance Study **23**

Peptide/Protein Experiments 24

General Information **24**

Allylation of Peptide **P1** **24**

Farnesylation of Peptide **P1** using Carbonate Reagent **Rb** **24**

Farnesylation of Peptide **P1** using Carbamate Reagent **Rc** **24**

Allylation of Proteins **24**

Farnesylation of Proteins **25**

Dialysis of UBL3 and Hsp27 ...... **25**

Circular Dichroism **25**

Pd Content Determination by ICP-MS **26**

References **27**

NMR-Spectra **28**

Supplementary Catalytic Data and Figures

n/*i* Selectivity

In a flame-dried and argon-flushed Schlenk flask, equipped with a Teflon-coated magnetic stirring bar, Pd(dba)$_2$ (6.4 μmol) and **L1** or **L2** (6.4 μmol) were suspended in 1 mL anhydrous ACN and stirred in a pre-heated oil bath at 60 °C for 30 min to obtain a bright yellow solution. Then methyl prenyl carbonate (0.384 mmol) and 1-octanethiol (0.320 mmol) were added and the resulting mixture was stirred at 60 °C for the time specified in the table below.

Table S1. Time dependence of the *n*/*i* ratio during the Pd-catalyzed prenylation of 1-octanethiol.

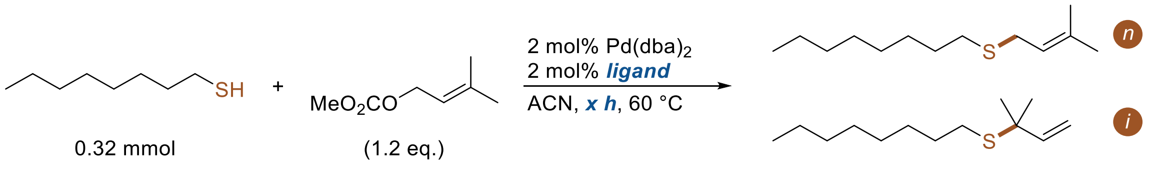

Entry	Time [h]	L1		L2	
		conv. [%][a]	*n*/*i* [a]	conv. [%][a]	*n*/*i* [a]
1	0.5	>99%	89/11	>99%	90/10
2	2	>99%	93/7	>99%	97/3
3	6	>99%	>99/1	>99%	>99/1

[a] Conversions as well as *n*/*i* ratios were determined by GC-MS without internal standard.

Farnesylation of P1 and UBL3

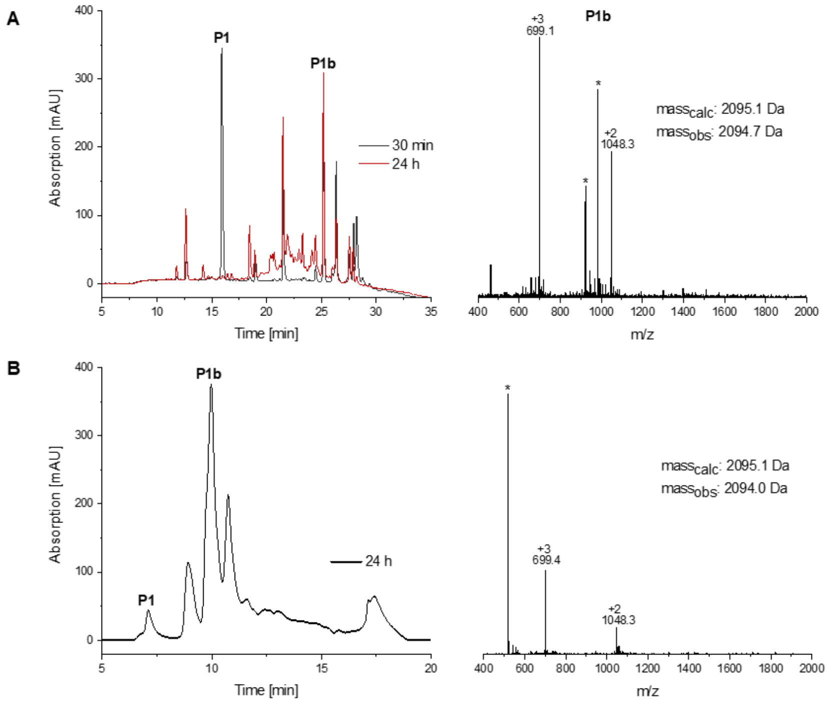

Figure S1: Analysis of **P1** farnesylation with ligand **L2** and farnesyl carbonate **Rb**. *A*: When using 15 vol% ACN as solvent, around 80% conversion could be observed after 24 h. *B*: Reducing the percentage of ACN to 5 vol% leads to around 50% conversion after 24 h. *Note:* Different retention times due to steeper HPLC gradient in *B* as compared to *A*.

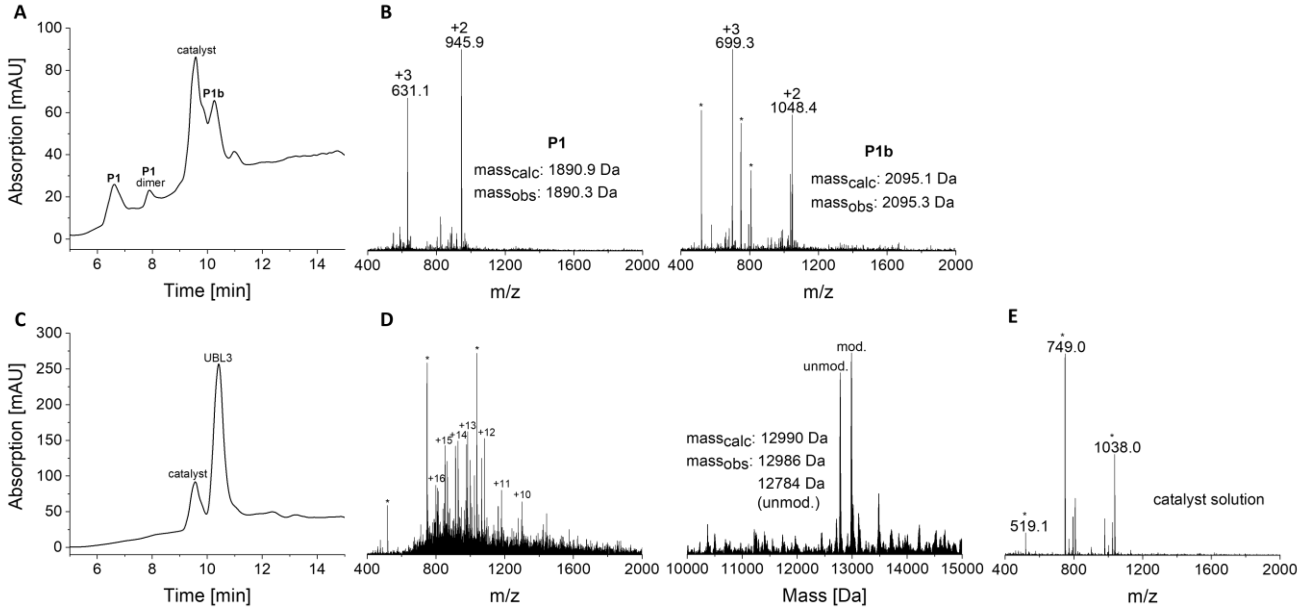

Figure S2: Analysis of **P1** and UBL3 farnesylation with ligand **L2** and farnesyl carbonate **Rc** after 5 min. Notably, the products coelute with the catalyst (*). *A*: HPLC analysis of farnesylated **P1**. *B*: MS spectra for unmodified and farnesylated **P1**. *C*: HPLC analysis of farnesylated UBL3. Unmodified and farnesylated UBL3 coelute. *D*: MS spectra and deconvoluted mass for unmodified and farnesylated UBL3. *E*: MS spectrum for the catalyst solution.

Removal of Catalyst by Dialysis

Dialysis of the reaction mixture against 50 mM phosphate buffer pH 7.0 after the allylation reaction removed most of the catalyst from the protein solution (below HPLC-MS detection limit). However, based on the shift during CD measurements we concluded that traces of the Pd-ligand still interfered with the protein. They could be successfully removed by incubation with 10 mM DTT.

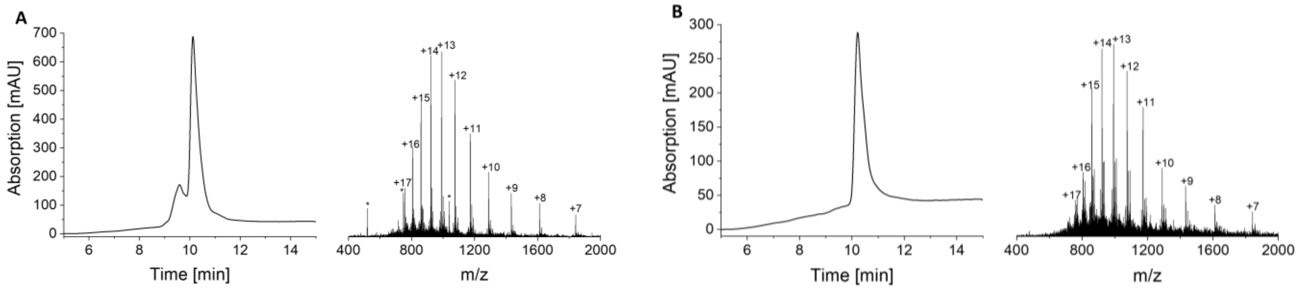

Figure S3: HPLC-MS analysis of UBL3 with an alkyne functionality before and after dialysis. *A*: Crude reaction mixture after modification with an alkyne. Pd-ligand signals are marked with *. *B*: Reaction mixture after dialysis against 50 mM phosphate buffer pH 7.0.

General Information

If reactions were performed under inert conditions, e.g. exclusion of water, oxygen or both, all experiments were carried out using established Schlenk techniques or inside a Glovebox (MBraun UNIlab pro). Herein solvents were dried and/or degassed with common methods and afterwards stored under inert gas atmosphere (argon or nitrogen) over molecular sieves. In some cases, when explicitly mentioned, dry solvents were received from the mentioned suppliers. In general, when high vacuum (*in vacuo*) was stated in experimental procedures, typically a vacuum of 10^{-2}-10^{-3} mbar was applied. Degassing of solvents or reaction mixtures was performed by bubbling argon from a balloon via cannula through the solvent or the reaction mixture during ultrasonication for about 20 min. All reagents were added in a counterstream of inert gas to keep the inert atmosphere. All reactions were stirred with Teflon-coated magnetic stirring bars.

Molecular sieves (Sigma-Aldrich, beads with 8-12 mesh) were activated in a round-bottom flask with a gas inlet adapter by heating them carefully in a heating mantle at level 1 at least for 24 h under high vacuum until complete dryness was obtained. These activated molecular sieves were stored at rt under argon atmosphere.

Temperatures were measured externally if not otherwise stated. When working at a temperature of 0 °C, an ice-water bath served as the cooling medium. Lower temperatures were achieved by using an acetone/dry ice cooling bath. Reactions, which were carried out at higher temperatures than rt, were heated in a silicon oil bath on a heating plate (RCT basic IKAMAG® safety control, 0-1500 rpm) equipped with an external temperature controller.

Chemicals

All commercially available chemicals and solvents were purchased from Acros Organics, Alfa Aesar, Fisher, Fluka, Honeywell, Merck, Roth, Sigma-Aldrich, TCI, VWR and used without further purification, unless otherwise stated. Protected Fmoc-amino acids, resins and coupling reagents were purchased from Novabiochem and Iris. The medium and buffers for protein expression and purification were prepared with substances from Roth, Sigma-Aldrich and PanReac AppliChem.

Acetonitrile: Anhydrous acetonitrile was purchased from Alfa Aesar. It was transferred into an amber 1 L Schlenk bottle and stored over activated 3 Å MS under argon atmosphere.

Dichloromethane: Anhydrous dichloromethane was produced by pre-drying EtOH stabilized dichloromethane over P_4O_{10} and afterwards heating it under reflux over CaH_2 for 24 h under argon atmosphere. It was distilled into an amber 1 L Schlenk bottle over activated 4 Å MS and under argon atmosphere.

N,*N*-Dimethylformamide: *N*,*N*-Dimethylformamide was purchased in extra dry quality from Alfa Aesar. It was transferred into an amber 1 L Schlenk bottle and stored over activated 4 Å MS under argon atmosphere.

Methanol: Methanol was purchased from Fisher and heated under reflux over Mg and I_2 for 2 h. It was distilled into an amber 1 L Schlenk bottle and stored over activated 3 Å MS under argon atmosphere.

NEt_3 (anhydrous): NEt_3 was dried over Na. It was distilled into an amber 1 L Schlenk bottle and stored over activated 4 Å molecular sieves under argon atmosphere.

Tetrahydrofuran: Tetrahydrofuran was purchased from VWR and heated under reflux over Na until benzophenone indicated dryness (intense blue color). It was distilled into an amber 1 L Schlenk bottle and stored over 4 Å molecular sieves under argon atmosphere.

Toluene: Toluene was purchased from Fisher and dried through an aluminium oxide column under inert conditions. It was filled into an amber 1 L Schlenk bottle and stored over activated 4 Å molecular sieves under argon atmosphere.

Solvents for peptide synthesis and chromatography were of “peptide synthesis grade” or “HPLC grade”.

Thin Layer Chromatography

Analytical thin layer chromatography (TLC) was carried out on Merck TLC silica gel aluminum sheets (silica gel 60, F254, 20 x 20 cm). All separated compounds were visualized by UV light (λ = 254 nm and/or λ = 366 nm) and by the listed staining reagents followed by the development in the heat.

$KMnO_4$: 3.0 g $KMnO_4$ as well as 20 g K_2CO_3 were dissolved in 300 mL H_2O and afterwards 5.0 mL 5 % aq. NaOH were added.

CAM: 50 g $(NH_4)_6Mo_7O_{24}$, 2.0 g $Ce(SO_4)_2$ and 50 mL conc. H_2SO_4 were dissolved in 400 mL water.

Flash Column Chromatography

Flash column chromatography was performed on silica gel 60 from Acros Organics with particle sizes between 35 µm and 70 µm. Depending on the problem of separation, a 30 to 100 fold excess of silica gel was used with respect to the dry amount of crude material. The dimension of the column was adjusted to the required amount of silica gel and formed a pad between 10 cm and 30 cm. In general, the silica gel was mixed with the eluent and the column was equilibrated. Subsequently, the crude material was dissolved in the eluent and loaded onto the top of the silica gel and the mobile phase was forced through the column using a rubber bulb pump. The volume of each collected fraction was adjusted between 20 % and 40 % of the silica gel volume.

Gas Chromatography

GC-MS analyses were performed on an Agilent Technologies 7890A GC system equipped with a 5975C mass selective detector (inert MSD with Triple Axis Detector system) by electron-impact ionization (EI) with a potential of E = 70 eV. Herein, the samples were separated depending on their boiling point and polarity. The desired crude materials or pure compounds were dissolved and the solutions were injected by employing the autosampler 7683B in a split mode 1/20 (inlet temperature: 280 °C; injection volume: 0.2 µL). Separations were carried out on an Agilent Technologies J&W GC HP-5MS capillary column ((5 %-phenyl)methylpolysiloxane, 30 m x 0.2 mm x 0.25 µm) with a constant helium flow rate (He 5.0 (Air Liquide), 1.085 $mL\cdot min^{-1}$, average velocity: 41.6 $cm\cdot s^{-1}$). A general gradient temperature method was used:

50S: initial temperature: 50 °C for 1 min; linear increase to 300 °C (40 $°C\cdot min^{-1}$); hold for 5 min; 1 min post-run at 300 °C; detecting range: 50.0-550.0 amu; solvent delay: 2.60 min.

High Performance Liquid Chromatography

Analytical HPLC-MS measurements were performed on a Shimadzu Nexera LCMS-2020 system (CBM-20A Prominence system controller, Nexera SIL-30AC autosampler, DGU-20A3 and DGU-20A5 on-line degassers, Nexera LC-30AD binary pump, FCV-20AH2 valve unit, CTO-20AC Prominence column oven, SPD-M20A Prominence photodiode array (PDA) detector (deuterium lamp, tungsten lamp, 190-800 nm)) equipped with single quadrupole ultra-fast LC/MS detector "LCMS-2020" or an Agilent Technologies 1200 Series system (G1379 Degasser, G1312 Binary Pump, G1367C HiP ALS SL Autosampler, G1330B FC/ALS Thermostat, G1316B TCC SL column compartment, G1365C MWD SL multiple wavelength detector (deuterium lamp, 190-400 nm)) equipped with a single quadrupole LCMS detector "6120 LC/MS" using electrospray ionization source (ESI in positive and negative mode). All separations were carried out on an Agilent Poroshell 120 SB-C18 (100 x 3.0 mm, 2.7 µm) column equipped with a Merck LiChroCART® 4-4 pre-column, or a reversed phase Agilent Poroshell 120 EC-C18 (100 x 3.0 mm, 2.7 µm) column equipped with a Merck LiChroCART® 4-4 pre-column. The following methods were used:

2-100-SB-C18: 0.0 min: 98 % H_2O + 0.01 % HCOOH and 2 % ACN; 0.0-6.0 min: linear gradient to 100 % ACN; 6.0-8.0 min: 100 % ACN; 8.0-8.5 min: linear gradient to 98 % H_2O + 0.01 % HCOOH and 2 % ACN; 8.5-9.5 min: 98 % H_2O + 0.01 % HCOOH and 2 % ACN; 0.700 mL·min^{-1}; 30 °C.

2-100-SB-C18-MeOH: 0.0 min: 98 % H_2O + 0.01 % HCOOH and 2 % MeOH; 0.0-6.0 min: linear gradient to 100 % MeOH; 6.0-8.0 min: 100 % MeOH; 8.0-8.5 min: linear gradient to 98 % H_2O + 0.01 % HCOOH and 2 % MeOH; 8.5-9.5 min: 98 % H_2O + 0.01 % HCOOH and 2 % MeOH; 0.700 mL·min^{-1}; 30 °C.

2-100-EC-C18: 0.0 min: 98 % H_2O + 0.05 % TFA and 2 % ACN; 0.0-6.0 min: linear gradient to 100 % ACN; 6.0-6.5 min: 100 % ACN; 6.5-6.51 min: linear gradient to 98 % H_2O + 0.05 % TFA and 2 % ACN; 6.51-8.5 min: 98 % H_2O + 0.05 % TFA and 2 % ACN; 0.700 mL·min^{-1}; 35 °C.

Nuclear Magnetic Resonance Spectroscopy

NMR spectra were recorded on a Bruker AVANCE III 300 spectrometer (^{1}H: 300.36 MHz; ^{13}C: 75.53 MHz) with autosampler, or a Varian Unity Inova 500 spectrometer (^{1}H: 499.87 MHz; ^{13}C: 125.69 MHz, ^{31}P: 202.35 MHz), or a Joel JNM-ECZL spectrometer (^{1}H: 399.78 MHz; ^{13}C: 100.35 MHz, ^{31}P: 161.83 MHz). Chemical shifts δ are referenced to the residual proton and carbon signal of the deuterated solvent ($CDCl_3$: δ = 7.26 ppm (^{1}H), 77.16 ppm (^{13}C); CD_2Cl_2: δ = 5.32 ppm (^{1}H), 53.84 ppm (^{13}C); DMSO-d_6: δ = 2.50 ppm (^{1}H), 39.52 ppm (^{13}C); CD_3OD: δ = 3.31 ppm (^{1}H), 49.00 ppm (^{13}C); D_2O: δ = 4.79 ppm (^{1}H)). Chemical shifts δ are given in ppm (parts per million) and coupling constants *J* in Hz (Hertz). If necessary, 1D spectra (APT and NOESY) as well as 2D spectra (H,H-COSY, HSQC, HMBC) were recorded for the identification and confirmation of the structure. Signal multiplicities are abbreviated as s (singlet), br s (broad singlet), d (doublet), dd (doublet of doublet), td (triplet of doublet), t (triplet), dt (doublet of triplet), q (quadruplet), p (pentet) and m (multiplet). Deuterated solvents for nuclear resonance spectroscopy were purchased from euriso-top®.

High Resolution Mass Spectrometry

High-resolution mass spectra were recorded on a Waters Micromass GCT Premier system. Ionization was realized by an electron impact source (EI ionization) at a constant potential of 70 eV. Herein, individual samples were either inserted directly (direct inlet electron impact ionization; DI-EI) or prior to this gas chromatographically separated on an Agilent 7890A system equipped with an Agilent Technologies J&W GC-column DB-5MS (length: 30 m; inner-diameter: 0.250 mm; film: 0.25 µm) at a constant helium flow. Molecule ions were analyzed by a time-of-flight (TOF) mass analyzer in the positive mode (TOF MS EI+).

Further high-resolution mass spectra were recorded using MALDI TOF on a Waters Micromass® MALDI micro MX Mass spectrometer. Dithranol (1,8-dihydroxy-9,10-dihydroanthracen-9-one) served as matrix and PEG as internal standard. Besides molecular formulas, calculated as well as determined *m/z* ratios of each molecule peak are denoted.

Further high-resolution mass spectra were recorded using a Bruker maXis UHR-TOF system (Qq-TOF instrument). The samples were injected directly, ionized via electrospray ionization (ESI) and analyzed in positive mode.

Further high-resolution mass spectra (LC-ESI-MS/MS) were acquired by data-dependent high-resolution tandem mass spectrometry on a QExactive Focus (Thermo Fisher Scientific, Germany). The electrospray ionization potential was set to +3.5 or -3.0 kV, the sheath gas flow was set to 20, and an auxiliary gas flow of 5 was used. Samples were diluted with an appropriate solvent (methanol or chloroform) and 1 µL was injected on a SeQuant®ZIC®-pHILIC HPLC column (Merck, 100 x 2.1 mm; 5 µm; 100 Å; peek coated; equipped with a guard column) or on a RP-column (Waters, ACQUITY UPLC HSS T3 150 x 2.1 mm; 1.8 µm with VanGuard column). The separation solvent (pHILIC: A: ACN, B: 25 mM NH_4HCO_3; RP: A: 0.1% HCOOH, B: 0.1% HCOOH in ACN) was delivered through an Ultimate 3000 HPLC system (Thermo Fisher Scientific, Germany) with a flow rate of 100 $\mu L{\cdot}min^{-1}$ and appropriate gradients were used for proper sample elution.

Inductively coupled plasma mass spectrometry (ICP-MS) was carried out on an Agilent ICP-MS 780 with an Agilent SPS 4 autosampler. The following settings were used to monitor ^{115}In and ^{106}Pd isotopes: RF power 1550, carrier gas at 1.08 $L{\cdot}min^{-1}$, plasma gas at 15 $L{\cdot}min^{-1}$, integration time 0.1 s, number of replicates 6 and number of sweeps 100. The limit of Pd quantification for this instrument is 0.026 $\mu g{\cdot}L^{-1}$.

Determination of Melting Points

Melting points were determined on a Mel-Temp® melting point apparatus from Electrothermal with an integrated microscopical support. They were measured in open capillary tubes with a mercury-in-glass thermometer and were not corrected.

Experimental Procedures

Ligand Synthesis

2-(*tert*-Butyl)-4-(prop-2-yn-1-yloxy)phenol (S1)

This compound was prepared according to a procedure described by Salunke et al.[1]

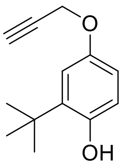

In a 500 mL three-necked round-bottom flask, equipped with a Teflon-coated magnetic stirring bar, air condenser and dropping funnel, 2-(*tert*-butyl)benzene-1,4-diol (3.29 g, 19.8 mmol) and K_2CO_3 (2.63 g. 19.0 mmol) were suspended in butanone (200 mL). Then a solution of propargyl bromide (2.23 g, 18.8 mmol) in butanone (100 mL) was added dropwise over a period of 30 min. The reddish suspension was heated under reflux for 17 h (reaction monitoring via TLC). The decolorized suspension was allowed to cool to r.t., filtered, washed with butanone (3 x 20 mL) and concentrated under reduced pressure. The crude product was purified via flash column chromatography (250 g SiO_2, 20.0 x 5.5 cm, cyclohexane:EtOAc = 9:1 (v/v)) to afford the desired compound as a red-brown oil (1.19 g, 31%).

$C_{13}H_{16}O_2$ [204.27 g·mol^{-1}]
R_f = 0.27 (cyclohexane:EtOAc = 9:1 (v/v), CAM)
GC-MS (method: 50S): t_R = 5.90 min; *m/z* (%) = 204 (42), 189 (14), 165 (100), 149 (39).
^{1}H-NMR (300 MHz, $CDCl_3$): δ = 6.95 (d, *J* = 2.8 Hz, 1H), 6.70 (dd, *J* = 8.5, 2.9 Hz, 1H), 6.60 (d, *J* = 8.5 Hz, 1H), 4.63 (d, *J* = 2.2 Hz, 2H), 4.58 (s, 1H), 2.51 (t, *J* = 2.1 Hz, 1H), 1.40 (s, 9H).
^{13}C-NMR (76 MHz, $CDCl_3$): δ = 151.6, 149.1, 137.8, 116.9, 115.6, 112.2, 79.2, 75.4, 56.7, 34.8, 29.6.
Analytical data are in accordance with the literature.[1]

3,3'-Di-*tert*-butyl-5,5'-bis(prop-2-yn-1-yloxy)-[1,1'-biphenyl]-2,2'-diol (S2)

This compound was prepared similar to a procedure described by van der Vlugt et al.[2]

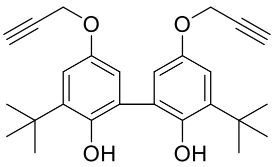

In a 100 mL round-bottom flask, equipped with a Teflon-coated magnetic stirring bar, **S1** (1.03 g, 5.07 mmol) was dissolved in MeOH (25 mL). Then a solution of $K_3[Fe(CN)_6]$ (1.68 g, 5.10 mmol) and KOH (1.18 g, 21.1 mmol) in water (25 mL) was added dropwise over a period of 30 min to give an orange suspension, which was stirred at r.t. for 3 h (reaction monitoring via TLC). Afterwards the reaction mixture was diluted with water (50 mL) and extracted with EtOAc (2 x 50 mL) and Et_2O (25 mL). The combined organic layers were dried over Na_2SO_4, filtered and concentrated under reduced pressure to afford the desired compound as a brown solid (1.05 g, quant.).

$C_{26}H_{30}O_4$ [406.52 g·mol^{-1}]

R_f = 0.54 (cyclohexane:EtOAc = 9:1 (v/v), $KMnO_4$)

HPLC-MS (method: 2-100-EC-C18): t_R = 6.68 min; *m/z* (ESI+) = 445 $[M+K]^+$.

mp = 85 °C

^{1}H-NMR (300 MHz, $CDCl_3$): δ = 7.05 (d, *J* = 2.8 Hz, 2H), 6.72 (d, *J* = 2.9 Hz, 2H), 5.10 (s, 2H), 4.65 (d, *J* = 2.1 Hz, 4H), 2.53 (t, *J* = 2.1 Hz, 2H), 1.43 (s, 18H).

^{13}C-NMR (76 MHz, $CDCl_3$): δ = 151.3, 146.8, 139.3, 123.0, 116.5, 113.5, 78.9, 75.7, 56.7, 35.4, 29.6.

HRMS (MALDI-TOF): calc. for $C_{26}H_{30}O_4^+$ $[M]^+$: 406.2144; found: 406.2215. (*Note: 17 ppm deviation)*

4,8-Di-*tert*-butyl-6-((2'-(dibenzo[*d*,*f*][1,3,2]dioxaphosphepin-6-yloxy)-[1,1'-biphenyl]-2-yl)oxy)-2,10-bis(prop-2-yn-1-yloxy)dibenzo[*d*,*f*][1,3,2]dioxaphosphepine (S3)

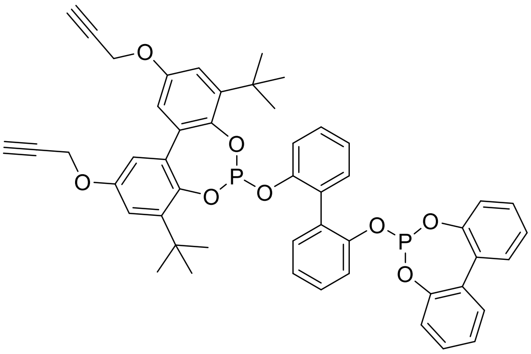

In an evacuated and argon-flushed 25 mL round-bottom flask, equipped with a Teflon-coated magnetic stirring bar, **6** (399 mg, 1.59 mmol) was cooled to 0 °C (ice bath). Then a solution of **S2** (301 mg, 0.740 mmol) and anhydrous NEt_3 (0.72 mL, 5.2 mmol) in anhydrous toluene (15 mL) was added dropwise over a period of 30 min. The yellow-orange suspension was stirred at 0 °C for 10 min and for an additional 16 h at r.t. (reaction monitoring via HPLC-MS). Subsequently, the reaction mixture was concentrated under reduced pressure. The crude product was purified via flash column chromatography (100 g SiO_2, 18.5 x 4.0 cm, cyclohexane:EtOAc = 20:1 to 10:1 (v/v)) to afford the desired compound as a yellow solid (520 mg, 84%).

$C_{50}H_{44}O_8P_2$ [834.84 g·mol^{-1}]

R_f = 0.32 (cyclohexane:EtOAc = 9:1 (v/v), $KMnO_4$)

HPLC-MS (method: 2-100-EC-C18): t_R = 8.34 min; *m/z* (ESI+) = 835 $[M+H]^+$.

mp = 76-77 °C

^{1}H-NMR (300 MHz, CD_3CN): δ = 7.62-7.04 (m, 14H), 6.99 (d, *J* = 2.9 Hz, 2H), 6.96-6.84 (m, 2H), 6.80 (d, *J* = 2.9 Hz, 2H), 4.75 (d, *J* = 2.2 Hz, 4H), 2.82 (t, *J* = 2.1 Hz, 2H), 1.23 (s, 18H).

^{13}C-NMR (76 MHz, CD_3CN): δ = 154.8, 150.4 (d, *J* = 3.7 Hz), 150.1 (d, *J* = 7.9 Hz), 149.7 (d, *J* = 5.0 Hz), 143.8 (d, *J* = 1.6 Hz), 142.6 (d, *J* = 5.9 Hz), 134.2 (d, *J* = 3.9 Hz), 133.6, 133.1, 131.8 (d, *J* = 3.2 Hz), 131.1 (d, *J* = 3.5 Hz), 130.9 (d, *J* = 0.9 Hz), 130.8 (d, *J* = 2.4 Hz), 130.4, 130.3, 130.2, 126.6, 125.4, 125.0, 123.0 (d, *J* = 0.9 Hz), 121.7 (d, *J* = 13.1 Hz), 121.6 (d, *J* = 10.1 Hz), 116.4, 115.2, 79.7, 77.0, 56.9, 36.0, 31.1 (d, *J* = 2.6 Hz).

^{31}P-NMR (122 MHz, CD_3CN): δ = 144.7 (d, *J* = 3.5 Hz), 138.0 (d, *J* = 3.3 Hz).

HRMS (MALDI-TOF): calc. for $C_{50}H_{44}NaO_{10}P_2^+$ $[M+2O+Na]^+$: 889.2308; found: 889.2320.

Ethyl 2-(3-(*tert*-butyl)-4-hydroxyphenoxy)acetate (S4)

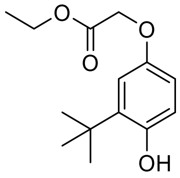

In a flame-dried, evacuated and nitrogen-flushed 500 mL three-neck round-bottom flask, equipped with a Teflon-coated magnetic stirring bar, dropping funnel, air condenser and bubbler, *tert*-butylhydroquinone (4.99 g, 30.0 mmol) and K_2CO_3 (8.28 g, 59.9 mmol) were suspended in anhydrous ACN (100 mL) and heated under reflux in an oil bath. Subsequently, a solution of ethyl bromoacetate (2.52 g, 15.1 mmol) in anhydrous ACN (10 mL) was added over a period of 30 min and the reaction mixture was heated under reflux for an additional 30 min (reaction monitoring via GC-MS). The red suspension was allowed to cool to r.t. and after evaporation of ACN the residue was extracted using CH_2Cl_2 (250 mL) and satd. NH_4Cl solution (125 mL). The aqueous layer was then acidified with 1 M aq. HCl to pH 5-6 and back-extracted with CH_2Cl_2 (2 x 125 mL). The combined organic layers were dried over Na_2SO_4, filtered and concentrated *in vacuo*. Purification via flash column chromatography (500 g SiO_2, 20.5 x 8.0 cm, toluene:EtOAc = 20:1 (v/v)) provided the desired compound as an off-white solid (2.35 g, 62%).

$C_{14}H_{20}O_4$ [252.31 g·mol^{-1}]
R_f = 0.36 (toluene:EtOAc = 10:1 (v/v), CAM)
GC-MS (method: 50S): t_R = 6.62 min; *m/z* (%) = 252 (59), 237 (100), 209 (15), 179 (8), 163 (26).
mp = 64 °C
^{1}H-NMR (300 MHz, $CDCl_3$): δ = 6.92 (s, 1H), 6.57 (m, 2H), 4.81 (s, 1H), 4.55 (s, 2H), 4.28 (q, *J* = 7.1 Hz, 2H), 1.38 (s, 9H), 1.30 (t, *J* = 7.1 Hz, 3H).
^{13}C-NMR (76 MHz, $CDCl_3$): δ = 169.7, 151.8, 149.3, 137.8, 116.9, 115.4, 111.9, 66.6, 61.5, 34.8, 29.6, 14.3.
HRMS (GC-EI TOF): calcd. for $C_{14}H_{20}O_4^+$ [M]$^+$: 252.1362; found: 252.1364.

2,2'-((5,5'-Di-*tert*-butyl-6,6'-dihydroxy-[1,1'-biphenyl]-3,3'- diyl)bis(oxy))diacetic acid (S5)

This compound was prepared similar to the literature.[2]

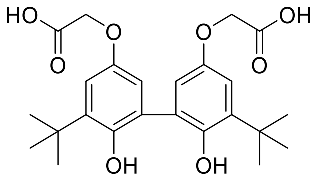

In a 500 mL one-neck round-bottom flask, equipped with a Teflon-coated magnetic stirring bar and dropping funnel, **S4** (2.00 g, 7.93 mmol) were dissolved in MeOH (40 mL). Subsequently, a solution of $K_3[Fe(CN)_6]$ (2.87 g, 8.72 mmol) and KOH (3.30 g, 58.8 mmol) in H_2O (40 mL) was added at r.t. over a period of 30 min. The resulting red suspension was stirred for an additional 1.5 h at r.t. (reaction monitoring via HPLC-MS). After the addition of H_2O (40 mL), the solution was carefully acidified with 1 M aq. HCl to pH 3.0 (pH meter). In order to check for HCN exposure a HCN sensor was placed next to the reaction vessel. The yellow suspension was then extracted with CH_2Cl_2 (3 x 150 mL). The combined organic layers were dried over Na_2SO_4, filtered and concentrated *in vacuo* to give the pure title compound as an off-white solid (1.70 g, 96%), which was used without further purification.

$C_{24}H_{30}O_8$ [446.50 g·mol^{-1}]
HPLC-MS (method: 2-100-SB-C18-MeOH): t_R = 6.95 min; *m/z* (ESI-) = 445.2 [M-H]$^-$.
mp = 102-104 °C
^{1}H-NMR (300 MHz, DMSO-d_6): δ = 12.93 (br s, 2H), 8.69 (s, 2H), 6.82 (d, *J* = 2.5 Hz, 2H), 6.66 (d, *J* = 2.6 Hz, 2H), 4.65 (s, 4H), 1.38 (s, 18H).
^{13}C-NMR (76 MHz, DMSO-d_6): δ = 170.6, 151.6, 145.5, 140.7, 130.6, 113.9, 113.6, 65.0, 34.8, 29.7.
HRMS (DI-EI TOF): calcd. for $C_{24}H_{30}O_8^+$ [M]$^+$: 446.1941; found: 446.1944.

Dimethyl 2,2'-((5,5'-di-*tert*-butyl-6,6'-dihydroxy-[1,1'-biphenyl]- 3,3'-diyl)bis(oxy))diacetate (S6)

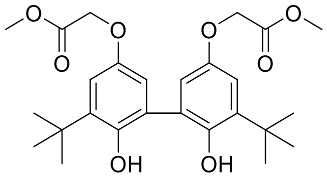

In a 5 mL one-neck round-bottom flask, equipped with a Teflon-coated magnetic stirring bar, **S5** (40.0 mg, 89.6 µmol) were dissolved in MeOH (1 mL) and treated with one drop conc. H_2SO_4 (~0.3 mmol). The resulting orange solution was stirred for 3 h at r.t. (reaction monitoring via HPLC-MS), quenched by the addition of satd. $NaHCO_3$ (2 mL) solution and extracted with CH_2Cl_2 (3 x 2 mL). The combined organic layers were dried over Na_2SO_4, filtered and concentrated *in vacuo* to give the pure title compound as an off-white solid (41.0 mg, 96%), which was used without further purification.

$C_{26}H_{34}O_8$ [474.55 g·mol^{-1}]
R_f = 0.41 (cyclohexane:EtOAc = 3:1 (v/v), $KMnO_4$)
HPLC-MS (method: 2-100-SB-C18-MeOH): t_R = 7.08 min; *m/z* (ESI-) = 473.2 [M-H]$^-$.
mp = 139-140 °C
^{1}H-NMR (300 MHz, $CDCl_3$): δ = 7.02 (d, *J* = 2.8 Hz, 2H), 6.56 (d, *J* = 2.9 Hz, 2H), 5.07 (s, 2H), 4.58 (s, 4H), 3.80 (s, 6H), 1.41 (s, 18H).
^{13}C-NMR (76 MHz, $CDCl_3$): δ = 169.7, 151.6, 146.9, 139.5, 123.0, 116.5, 113.0, 66.2, 52.4, 35.4, 29.6.
HRMS (EI-DI TOF): calcd. for $C_{26}H_{34}O_8^+$ [M]$^+$: 474.2254; found: 474.2258.

Dimethyl 2,2'-((4,8-di-*tert*-butyl-6-((2'-(dibenzo[*d*,*f*][1,3,2]dioxa-phosphepin-6-yloxy)-[1,1'-biphenyl]-2-yl)oxy)dibenzo[*d*,*f*][1,3,2]- dioxaphosphepin-2,10-diyl)bis(oxy))diacetate (S7)

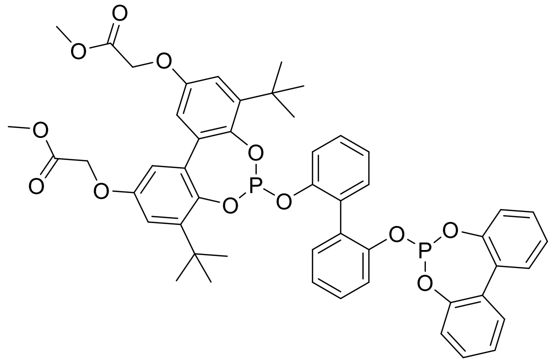

In a flame-dried, evacuated and argon-flushed 10 mL one-neck round-bottom flask, equipped with a Teflon-coated magnetic stirring bar, **6** (16.0 mg, 63.8 µmol) were cooled to 0 °C. Then a solution of **S6** (14.6 mg, 30.8 µmol) and anhydrous NEt_3 (0.03 mL, 0.2 mmol) in anhydrous toluene (0.5 mL) was added dropwise over a period of 5 min and the flask containing the former solution was rinsed with anhydrous toluene (0.25 mL). The resulting reaction mixture was stirred for 10 min at 0 °C and for an additional 46 h at r.t. (reaction monitoring via HPLC-MS). Subsequently, H_2O (1 mL) was added to the yellow-brownish suspension, the organic layer was separated and the aqueous layer was extracted with EtOAc (2 x 1 mL). The combined organic layers were dried over Na_2SO_4, filtered and concentrated *in vacuo*. Purification via flash column chromatography (5 g SiO_2, 8.5 x 1.3 cm, cyclohexane:EtOAc = 3:1 (v/v)) provided the title compound as a colorless solid (12.8 mg, 46%).

$C_{50}H_{48}O_{12}P_2$ [902.87 g·mol^{-1}]

R_f = 0.30 (cyclohexane:EtOAc = 3:1 (v/v), CAM)

HPLC-MS (method: 2-100-SB-C18): t_R = 8.13 min; *no mass signal could be detected*.

^{1}H-NMR (500 MHz, C_6D_6): δ = 7.40 (dd, *J* = 7.6, 1.5 Hz, 1H), 7.34-7.27 (m, 3H), 7.19-7.16 (m, 2H), 7.12 (d, *J* = 3.1 Hz, 2H), 7.09 (td, *J* = 8.1, 1.6 Hz, 1H), 7.04 (td, *J* = 8.0, 1.6 Hz, 1H), 6.99 (td, *J* = 7.6, 1.6 Hz, 2H), 6.96-6.86 (m, 6H), 6.59 (d, *J* = 3.0 Hz, 2H), 4.18 (s, 4H), 3.29 (s, 6H), 1.32 (s, 18H).

^{13}C-NMR (126 MHz, C_6D_6): δ = 169.0, 154.9, 150.0, 149.9, 149.9 (d, *J* = 5.2 Hz), 143.4, 142.9 (d, *J* = 5.8 Hz), 134.2 (d, *J* = 3.2 Hz), 133.2, 132.8, 131.8 (d, *J* = 2.5 Hz), 130.8 (d, *J* = 2.6 Hz), 130.6, 130.1, 129.3, 129.2, 129.0, 125.4, 124.3, 124.0, 122.6, 121.5 (d, *J* = 11.4 Hz), 121.2 (d, *J* = 10.2 Hz), 116.1, 114.1, 65.9, 51.5, 35.5, 31.0.

^{31}P-NMR (202 MHz, C_6D_6): δ = 144.7 (d, *J* = 4.0 Hz), 138.7 (d, *J* = 3.8 Hz), small resonance at 146.0 (9 %).

5,5'-Dibromo-[1,1'-biphenyl]-2,2'-diol (2)

This compound was prepared similar to a procedure described by Zhao et al.[3]

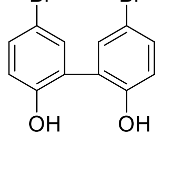

In a 1 L round-bottom flask, equipped with a Teflon-coated magnetic stirring bar, 2,2'-biphenol (10.0 g, 53.9 mmol) was dissolved in $CHCl_3$ (200 mL). Then a solution of Br_2 (5.8 mL, 113 mmol) in $CHCl_3$ (40 mL) was added dropwise over a period of 70 min and the reaction mixture was stirred at r.t. for 1.5 h (reaction monitoring via TLC). Afterwards

the reaction mixture was cooled to 0 °C. The resulting precipitate was isolated by filtration, washed with cold $CHCl_3$ (2 x 20 mL) and dried *in vacuo* to afford the desired compound as a colorless solid (13.9 g, 75%).

$C_{12}H_8Br_2O_2$ [344.00 g·mol^{-1}]
R_f = 0.61 (cyclohexane:EtOAc = 1:1 (v/v), CAM)
^{1}H-NMR (300 MHz, DMSO-d_6): δ = 9.79 (br s, 2H), 7.36-7.16 (m, 4H), 6.84 (d, *J* = 8.6 Hz, 2H).
^{13}C-NMR (76 MHz, DMSO-d_6): δ = 154.2, 133.7, 131.3, 126.8, 118.0, 109.8.
Analytical data are in accordance with the literature.[4]

5,5'-Dimethoxy-[1,1'-biphenyl]-2,2'-diol (3)

This compound was prepared similar to a procedure described by Capdevielle et al.[5]

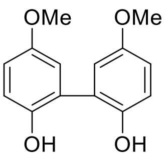

In a flame-dried and argon-flushed 100 mL Schlenk flask, equipped with a Teflon-coated magnetic stirring bar, sodium (8.24 g, 358 mmol) was added portionwise to anhydrous MeOH (60 mL). Upon complete dissolution EtOAc (3.2 mL, 32.8 mmol), **2** (10.0 g, 29.0 mmol), and CuBr (1.68 g, 11.7 mmol) were added. The reaction mixture was heated to 70 °C and stirred for 24 h (reaction monitoring via HPLC-MS). After cooling to r.t., the suspension was poured into ice-cold 1 M HCl (440 mL). The aqueous layer was back-extracted with EtOAc (3 x 240 mL). The combined organic layers were dried over Na_2SO_4, filtered and concentrated under reduced pressure. The crude product was purified by recrystallization from EtOAc to afford the desired compound as reddish crystals (3.30 g, 46%).

$C_{10}H_{14}O_4$ [246.26 g·mol^{-1}]
R_f = 0.56 (cyclohexane:EtOAc = 1:1 (v/v), $KMnO_4$)
HPLC-MS (method: 2-100-EC-C18): t_R = 4.42 min; *m/z* (ESI+) = 247 [M+H]$^+$.
mp = 117-119 °C
^{1}H-NMR (300 MHz, $CDCl_3$): δ = 6.97 (d, *J* = 8.8 Hz, 2H), 6.89 (dd, *J* = 8.8, 2.9 Hz, 2H), 6.82 (d, *J* = 2.9 Hz, 2H), 5.27 (s, 2H), 3.80 (s, 6H).
^{13}C-NMR (76 MHz, $CDCl_3$): δ = 154.3, 146.7, 125.5, 118.0, 116.3, 115.6, 56.0.
Analytical data are in accordance with the literature.[6]

5,5'-Dimethoxy-[1,1'-biphenyl]-2,2'-diyl tetraethyl bis(phosphate) (4)

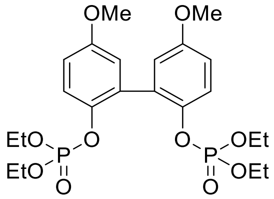

In a flame-dried and argon-flushed 100 mL Schlenk flask, equipped with a Teflon-coated magnetic stirring bar, **3** (1.01 g, 4.09 mmol) was dissolved in anhydrous ACN (25 mL) and cooled to -15 °C. Subsequently, CCl_4 (4.0 mL, 39.8 mmol), DMAP (110 mg, 0.884 mmol), and DIPEA (2.9 mL, 16.7 mmol) were added. Afterwards, diethyl phosphite (1.5 mL, 11.8 mmol) was added over a period of 3 min. The reaction mixture was slowly warmed to r.t. and stirred for 3 h (reaction monitoring via HPLC-MS). Afterwards, the reaction mixture was quenched by the addition of 0.5 M KH_2PO_4 (8.4 mL) and the aqueous layer was back-extracted with EtOAc (3 x 10 mL). The combined organic layers were dried over Na_2SO_4, filtered and concentrated under reduced pressure. The crude product was purified via flash column chromatography (150 g SiO_2, 20 x 6 cm, toluene:EtOAc = 1:6 to 1:20 (v/v)) to afford the desired compound as an off-white solid (1.82 g, 86%).

$C_{22}H_{32}O_{10}P_2$ [518.44 g·mol^{-1}]
R_f = 0.17 (toluene:EtOAc = 1:2 (v/v), $KMnO_4$)
HPLC-MS (method: 2-100-EC-C18): t_R = 5.07 min; *m/z* (ESI+) = 519 $[M+H]^+$.
mp = 58-60 °C
^{1}H-NMR (300 MHz, $CDCl_3$): δ = 7.34 (d, *J* = 8.9 Hz, 2H), 6.91 (d, *J* = 2.3 Hz, 2H), 6.86 (dd, *J* = 8.9, 3.1 Hz, 2H), 4.02-3.84 (m, 8H), 3.79 (s, 6H), 1.18 (t, *J* = 6.8 Hz, 12H).
^{13}C-NMR (76 MHz, $CDCl_3$): δ = 156.2 (d, *J* = 0.9 Hz), 142.1 (d, *J* = 6.7 Hz), 130.2 (dd, *J* = 7.1, 0.5 Hz), 120.7 (d, *J* = 2.2 Hz), 116.8, 114.6 (d, *J* = 1.2 Hz), 64.4 (d, *J* = 6.4 Hz), 55.9, 16.1 (d, *J* = 6.8 Hz).
^{31}P-NMR (81 MHz, $CDCl_3$): δ = -6.4.
HRMS (DI-ESI): calc. for $C_{22}H_{33}O_{10}P_2^+$ $[M+H]^+$: 519.1543; found: 519.1545.

Tetraethyl (2,2'-dihydroxy-5,5'-dimethoxy-[1,1'-biphenyl]-3,3'-diyl)bis(phosphonate) (5)

This compound was prepared similar to a procedure described Melvin et al.[7]

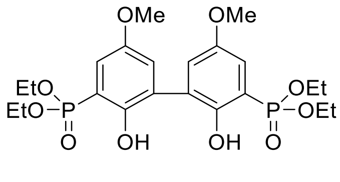

In a flame-dried and argon-flushed Schlenk flask, equipped with a Teflon-coated magnetic stirring bar, *i*Pr_2NH (0.900 mL, 6.28 mmol) was dissolved in anhydrous THF (20 mL) and cooled to -78 °C. Subsequently, *n*BuLi (4.7 mL, 1.25 M in hexanes, 5.88 mmol) was added over a period of 4 min. Then a solution of **4** (1.30 g, 2.51 mmol) in anhydrous THF (20 mL) was added over a period of 10 min. The resulting mixture was stirred at -78 °C for 15 min and at -10 °C for 50 min (reaction monitoring via HPLC-MS, *note: higher reaction temperature may lead to dephosphorylation*). Afterwards, the reaction mixture was quenched by addition of satd. NH_4Cl (50 mL). The layers were separated and the aqueous layer was back-extracted with EtOAc (3 x 100 mL). The combined organic layers

were dried over Na_2SO_4, filtered and concentrated under reduced pressure to afford the desired compound as a brown oil (1.37 g, quant.) which was used without further purification.

$C_{22}H_{32}O_{10}P_2$ [518.44 g·mol^{-1}]
R_f = 0.35 (toluene:EtOAc = 1:1 (v/v), $KMnO_4$)
HPLC-MS (method: 2-100-EC-C18): t_R = 5.58 min; *m/z* (ESI+) = 519 $[M+H]^+$.
^{1}H-NMR (300 MHz, $CDCl_3$): δ = 9.99 (s, 2H), 7.16 (d, *J* = 3.2 Hz, 2H), 6.91 (dd, *J* = 15.5, 3.2 Hz, 2H), 4.28-3.99 (m, 8H), 3.78 (s, 6H), 1.35 (t, *J* = 7.1 Hz, 12H).
^{13}C-NMR (76 MHz, $CDCl_3$): δ = 153.4 (d, *J* = 7.1 Hz), 152.0 (d, *J* = 18.1 Hz), 127.2 (dd, *J* = 15.2, 2.9), 124.2 (d, *J* = 2.9 Hz), 115.1 (d, *J* = 6.9 Hz), 109.7 (d, *J* = 179.5 Hz), 63.0 (d, *J* = 4.8 Hz), 56.1, 16.4 (d, *J* = 6.6 Hz).
^{31}P-NMR (202 MHz, $CDCl_3$): δ = 22.0.
HRMS (DI-ESI): calc. for $C_{22}H_{33}O_{10}P_2^+$ $[M+H]^+$: 519.1543; found: 519.1538.

6-Chlorodibenzo[*d*,*f*][1,3,2]dioxaphosphepine (6)

This compound was prepared according to a procedure described by Cuny et al.[8]

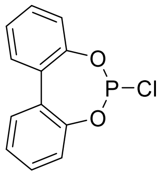

In a flame-dried and nitrogen-flushed 25 mL two-neck round-bottom flask, equipped with a Teflon-coated magnetic stirring bar, air condenser and bubbler, 2,2′-biphenol (4.59 g, 24.7 mmol.) and PCl_3 (8.0 mL, 92 mmol) were heated under reflux in an oil bath for 3 h. The resulting yellowish solution was allowed to cool to r.t., before the excess of unreacted PCl_3 was removed under reduced pressure (0.6-1.0 mbar, 40-50 °C). The remaining residue was purified via vacuum distillation (0.68 mbar, 140-142 °C) to afford the pure title compound as a colorless, highly viscous oil (4.09 g, 66%), which crystallizes upon standing in an inert atmosphere.

$C_{12}H_8ClO_2P$ [250.62 g·mol^{-1}]
bp = 140-142 °C (0.68 mbar)
^{1}H-NMR (300 MHz, $CDCl_3$): δ = 7.43 (dd, *J* = 7.3, 1.6 Hz, 2H), 7.39-7.25 (m, 4H), 7.16 (d, *J* = 7.8 Hz, 2H).
^{13}C-NMR (76 MHz, $CDCl_3$): δ = 149.4 (d, *J* = 5.8 Hz), 131.1 (d, *J* = 3.3 Hz), 130.4 (d, *J* = 1.2 Hz), 129.6, 126.4, 122.4 (d, *J* = 2.1 Hz).
^{31}P-NMR (122 MHz, $CDCl_3$): δ = 179.5.
Analytical data are in accordance with the literature.[8]

Tetraethyl (2,2'-bis(dibenzo[*d*,*f*][1,3,2]dioxaphosphepin-6-yloxy)-5,5'-dimethoxy-[1,1'-biphenyl]-3,3'-diyl)bis(phosphonate) (L2)

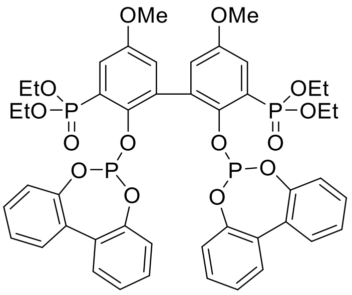

In a flame-dried and argon-flushed 25 mL round-bottom flask, equipped with a Teflon-coated magnetic stirring bar, **6** (702 mg, 2.80 mmol) was dissolved in anhydrous toluene (2 mL) and cooled to 0 °C. Subsequently, a solution of **5** (578 mg, 1.11 mmol) and anhydrous NEt_3 (0.78 mL, 5.60 mmol) in anhydrous toluene (10 mL) was added over a period of 5 min. The resulting mixture was allowed to warm to r.t. and stirred for 16 h (reaction monitoring via HPLC-MS). Afterwards, the reaction mixture was quenched by the addition of H_2O (20 mL) and diluted with EtOAc (20 mL). The aqueous layer was back-extracted with EtOAc (2 x 20 mL). The combined organic layers were dried over Na_2SO_4, filtered and concentrated under reduced pressure. The crude product was purified by recrystallization from anhydrous ACN under argon atmosphere to afford the desired compound as colorless crystals (825 mg, 78%). Note: *While the ligand is stable in air at room temperature (for purposes of purification, weighing, and transferring), we recommend storage in a freezer to maximize lifetime.*

$C_{46}H_{46}O_{14}P_4$ [946.76 g·mol^{-1}]
R_f = 0.38 (toluene:EtOAc = 1:4 (v/v), $KMnO_4$)
HPLC-MS (method: 2-100-EC-C18): t_R = 6.77 min; *m/z* (ESI+) = 947 [M+H]$^+$.
mp = 190-191 °C
^{1}H-NMR (300 MHz, CD_2Cl_2): δ = 7.55 (dd, *J* = 15.9, 3.2 Hz, 2H), 7.46-7.33 (m, 4H), 7.33-7.18 (m, 10H), 7.14 (d, *J* = 3.0 Hz, 2H), 6.91-6.77 (m, 2H), 4.18-3.92 (m, 4H), 3.87 (s, 6H), 3.84-3.57 (m, 4H), 1.17 (t, *J* = 7.0 Hz, 6H), 0.84 (t, *J* = 7.0 Hz, 6H).
^{13}C-NMR (76 MHz, CD_2Cl_2): δ = 156.1 (d, *J* = 18.2 Hz), 150.2-150.0 (m), 149.1, 146.1-145.8 (m), 133.6 (dd, *J* = 11.8, 2.8 Hz), 131.8, 131.0, 130.0, 129.7, 129.7, 129.4, 125.9, 125.5, 123.5 (d, *J* = 179.7 Hz), 123.4, 123.1 (d, *J* = 2.6 Hz), 122.9, 121.2 (d, *J* = 8.4 Hz), 62.8 (d, *J* = 5.0 Hz), 56.6, 16.6 (d, *J* = 7.0 Hz), 16.3 (d, *J* = 7.2 Hz).
^{31}P-NMR (202 MHz, CD_2Cl_2): δ = 144.8, 14.7.
HRMS (DI-ESI): calc. for $C_{46}H_{47}O_{14}P_4^+$ [M+H]$^+$: 947.1911; found: 947.1896.

Synthesis and Characterization of Diselenides

In a flame-dried and argon-flushed Schlenk flask, equipped with a Teflon-coated with magnetic stirring bar, BIPHEPHOS (**L1**, 27.1 mg, 34.4 µmol) and selenium (28.8 mg, 365 µmol) were suspended in anhydrous toluene (1.5 mL). The suspension was evacuated and argon-flushed three times and heated to 110 °C until complete consumption of the starting material (14 h). The reaction mixture was then allowed to cool to r.t. and the solvent was removed under reduced pressure. The crude product was dissolved in DMSO-d_6 (760 µL), filtered through a plug of cotton wool, and analyzed by ^{31}P-NMR spectroscopy.

^{31}P-NMR (162 MHz, DMSO-d_6): δ = 75.9, 75.9 (d, *J* = 1037 Hz).

In a flame-dried and argon-flushed Schlenk flask, equipped with a Teflon-coated with magnetic stirring bar, **L2** (12.0 mg, 12.7 µmol) and selenium (12.4 mg, 157 µmol) were suspended in anhydrous toluene (1 mL). The suspension was evacuated and argon-flushed three times and heated to 110 °C until complete consumption of the starting material (24 h). The reaction mixture was then allowed to cool to r.t. and the solvent was removed under reduced pressure. The crude product was dissolved in DMSO-d_6 (760 µL), filtered through a plug of cotton wool, and analyzed by ^{31}P-NMR spectroscopy.

^{31}P-NMR (162 MHz, DMSO-d_6): δ = 76.1, 76.1 (d, J = 1044 Hz), 13.9.

Synthesis of Allylic Reagents

Reagents methyl prenyl carbonate, **Ra**, and **Rb** were prepared according to previously published procedures.[9]

4-Nitrophenyl ((2*E*,6*E*)-3,7,11-trimethyldodeca-2,6,10-trien-1-yl) carbonate (S8)

This compound was prepared similar to a procedure described by Tilley et al.[10]

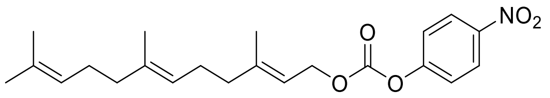

In a 50 mL round-bottom flask, equipped with a Teflon-coated magnetic stirring bar, 4-nitrophenyl chloroformate (502 mg, 2.49 mmol) were dissolved in CH_2Cl_2 (5 mL). Then a solution of *trans,trans*-farnesol (501 mg, 2.25 mmol) and pyridine (0.36 mL, 4.5 mmol) in CH_2Cl_2 (5 mL) was added dropwise over a period of 5 min and the resulting mixture was stirred at r.t. for 15 min. Upon complete consumption of the starting material (according to TLC), the reaction mixture was washed with 1 M HCl (2 x 10 mL) and satd. $NaHCO_3$ (5 mL), dried over Na_2SO_4, filtered and concentrated under reduced pressure. The crude product was purified via flash column chromatography (100 g SiO_2, 12.5 x 5.0 cm, cyclohexane:EtOAc = 20:1 (v/v)) to give the desired compound as a yellowish oil (583 mg, 67%).

$C_{22}H_{29}NO_5$ [387.48 g·mol^{-1}]
R_f = 0.51 (cyclohexane:EtOAc = 20:1 (v/v), $KMnO_4$)
^{1}H-NMR (300 MHz, $CDCl_3$): δ = 8.27 (d, *J* = 9.1 Hz, 2), 7.38 (d, *J* = 9.1 Hz, 2H), 5.45 (t, *J* = 6.8 Hz, 1H), 5.19-5.01 (m, 2H), 4.80 (d, *J* = 7.2 Hz, 2H), 2.23-1.89 (m, 8H), 1.77 (s, 3H), 1.68 (s, 3H), 1.60 (s, 6H).
^{13}C-NMR (76 MHz, $CDCl_3$): δ = 155.8, 152.7, 145.5, 145.0, 135.9, 131.5, 125.4, 124.4, 123.5, 121.9, 116.9, 66.2, 39.8, 39.7, 26.9, 26.2, 25.8, 17.8, 16.8, 16.2.
Analytical data are in accordance with the literature.[10]

Sodium 2-aminoethane-1-sulfonate (S9)

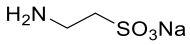

In a 50 mL round-bottom flask, equipped with a Teflon-coated magnetic stirring bar, a solution of NaOH (639 mg, 16.0 mmol) in H_2O (20 mL) was added to a suspension of 2-aminoethanesulfonic acid (2.00 g, 16.0 mmol) in H_2O (10 mL). The resulting colorless solution was stirred at r.t. for 5 min and lyophilized to afford the desired compound as a colorless solid (2.35 g, quant.).

$C_2H_6NNaO_3S$ [147.12 g·mol^{-1}]
mp = 188 °C
^{1}H-NMR (300 MHz, D_2O): δ = 3.13-2.87 (m, 4H).
^{13}C-NMR (76 MHz, D_2O): δ = 53.0, 36.5.

Sodium 2-(((((2*E*,6*E*)-3,7,11-trimethyldodeca-2,6,10-trien-1-yl)oxy)carbonyl)amino)ethane-1-sulfonate (Rc)

This compound was prepared according to a procedure described by Tilley et al.[10]

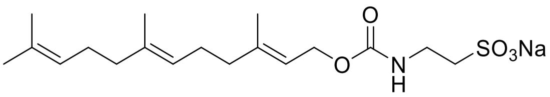

In an evacuated and argon-flushed 10 mL round-bottom flask, equipped with a Teflon-coated magnetic stirring bar, **S8** (559 mg, 1.44 mmol) were dissolved in anhydrous DMF (1.5 mL). Then **S9** (214 mg, 1.45 mmol) was added and the resulting yellow suspension was stirred at r.t. for 5 d. Upon complete consumption of the starting material (according to HPLC-MS), the reaction mixture was transferred to a 50 mL round-bottom flask (rinsing with 5 mL MeOH) and concentrated under reduced pressure (*note: intensive foaming*). The yellow residue was transferred onto a Celite column (9.5 x 3.0 cm) and washed consecutively with ACN (2 x 50 mL), 10% HOAc in ACN (2 x 50 mL), 25% HOAc in ACN (2 x 50 mL) and ACN (3 x 50 mL). The product was then eluted with MeOH (4 x 50 mL) and the filtrate was concentrated under reduced pressure to give the desired compound as an off-white solid (309 mg, 54%).

$C_{18}H_{30}NNaO_5S$ [395.49 g·mol^{-1}]
mp = 181-183 °C (decomp.)
HPLC-MS (method: 2-100-EC-C18): t_R = 5.28 min; *m/z* (ESI+) = 374 $[M-Na+2H]^+$.
^{1}H-NMR (300 MHz, D_2O): δ = 5.42-5.20 (m, 1H), 5.20-4.94 (m, 2H), 4.54 (d, *J* = 5.4 Hz, 2H), 3.60-3.35 (m, 2H), 3.17-2.93 (m, 2H), 2.23-1.82 (m, 8H), 1.67 (s, 3H), 1.63 (s, 3H), 1.55 (s, 6H).
^{13}C-NMR (76 MHz, D_2O): δ = 157.8, 142.0, 135.0, 130.8, 124.5, 124.0, 118.7, 62.0, 50.4, 39.6, 39.5, 36.4, 26.7, 26.3, 25.3, 17.3, 16.0, 15.7.
Analytical data are in accordance with the literature.[10]

Catalytic Performance Study

67 vol% ACN in H_2O: In a flame-dried and argon-flushed 10 mL Schlenk flask, $Pd(dba)_2$ (2 mol%, 2 μmol) and ligand (2 mol%, 2 μmol) were suspended in 0.70 mL anhydrous ACN and stirred in a pre-heated oil bath at 60 °C for 15 min to obtain a yellow solution. Subsequently, methyl prenyl carbonate (1.2 eq., 0.12 mmol) and a stock solution of GSH in degassed H_2O (0.33 mL, 92.4 mg·mL^{-1} GSH, 0.10 mmol) were added. The mixture was stirred at 60°C and the reaction was monitored by HPLC-MS analysis at different time points.

30 vol% ACN in H_2O: In a flame-dried and argon-flushed 10 mL Schlenk flask, $Pd(dba)_2$ (2 mol%, 3 μmol) and ligand (2 mol%, 3 μmol) were suspended in 0.45 mL anhydrous ACN and stirred in a pre-heated oil bath at 60 °C for 15 min to obtain a yellow solution. Subsequently, methyl prenyl carbonate (1.2 eq., 0.18 mmol) and a stock solution of GSH in degassed H_2O (1.0 mL, 46.3 mg·mL^{-1} GSH, 0.15 mmol) were added. The mixture was stirred at 60°C and the reaction was monitored by HPLC-MS analysis at different time points.

15 vol% ACN in H_2O: In a flame-dried and argon-flushed 10 mL Schlenk flask, $Pd(dba)_2$ (2 mol%, 6 μmol) and ligand (2 mol%, 6 μmol) were suspended in 0.45 mL anhydrous ACN and stirred in a pre-heated oil bath at 60 °C for 15 min to obtain a yellow solution. Subsequently, methyl prenyl carbonate (1.2 eq., 0.36 mmol) and a stock solution of GSH in degassed H_2O (2.5 mL, 36.9 mg·mL^{-1} GSH, 0.30 mmol) were added. The mixture was stirred at 60°C and the reaction was monitored by HPLC-MS analysis at different time points.

Peptide/Protein Experiments

General Information

All commercially available chemicals and solvents were purchased from Acros Organics, Alfa Aesar, Fisher, Fluka, Honeywell, Merck, Roth, Sigma-Aldrich, TCI, VWR and used without further purification, unless otherwise stated. Solvents for peptide synthesis and chromatography were of “peptide synthesis grade” and “HPLC grade”, respectively. Protected Fmoc-amino acids, resins and coupling reagents were purchased from Novabiochem and Iris. The medium and buffers for protein expression and purification were prepared with substances from Roth, Sigma-Aldrich and PanReac AppliChem.
The solid-phase peptide synthesis including purification and analysis as well as protein expression, purification and analysis were performed based on our published protocols.[9]

Allylation of Peptide P1

In a flame-dried and argon-flushed 10 mL Schlenk flask, $Pd(dba)_2$ (1.2 eq., 0.7 mg) and **L2** (1.2 eq., 1.1 mg) were suspended in 50 µL anhydrous ACN and stirred in a pre-heated oil bath at 60 °C for 15 min to obtain a yellow solution. After cooling down to 40 °C, the allylation reagent **Ra** (1.2 eq., 0.2 mg), the peptide **P1** (1 µmol, 1.9 mg) and 950 µL degassed H_2O were added. The mixture was stirred at 40°C and the reaction was monitored by HPLC-MS analysis at different time points.

Farnesylation of Peptide P1 using Carbonate Reagent Rb

In a flame-dried and argon-flushed 10 mL Schlenk flask, $Pd(dba)_2$ (1.2 eq., 0.7 mg) and **L2** (1.2 eq., 1.1 mg) were suspended in 50 µL anhydrous ACN and stirred in a pre-heated oil bath at 60 °C for 15 min to obtain a yellow solution. After cooling down to 40 °C, the farnesylation reagent **Rb** (1.2 eq., 0.5 mg), the peptide **P1** (1 µmol, 1.9 mg) and 950 µL degassed H_2O were added. The mixture was stirred at 40 °C and the reaction was monitored by HPLC-MS analysis at different time points.

Farnesylation of Peptide P1 using Carbamate Reagent Rc

In a flame-dried and argon-flushed 10 mL Schlenk flask, $Pd(dba)_2$ (1.2 eq., 0.7 mg) and **L2** (1.2 eq., 1.1 mg) were suspended in 50 µL anhydrous ACN and stirred in a pre-heated oil bath at 60°C for 15 min to obtain a yellow solution. After cooling down to 40°C, the farnesylation reagent **Rc** (1.2 eq., 0.5 mg), the peptide **P1** (1 µmol, 1.9 mg) and 950 µL degassed H_2O were added. The mixture was stirred at 40°C and the reaction was monitored by HPLC-MS analysis at different time points.

Allylation of Proteins

In a flame-dried and argon-flushed 10 mL Schlenk tube, $Pd(dba)_2$ (1.2 eq., 1 mg) and the ligand **L2** (1.2 eq., 1.4 mg) were suspended in 300 µL anhydrous ACN and stirred in a pre-heated oil bath at 60°C for 15 min. After cooling down to 40°C, the allylation reagent **Ra** (1.2 eq., 0.3 mg) was added to the yellow solution to obtain the catalyst stock-solution.

In a second argon-flushed 10 mL Schlenk tube, the desired protein (0.25 µmol, UBL3: 3.2 mg, Hsp27: 5.7 mg) was dissolved in 950 µL degassed H_2O. 50 µL of the catalyst stock solution were added and the reaction mixture was stirred at 40°C and the reaction was monitored by HPLC-MS analysis at different time points.

Farnesylation of Proteins

In a flame-dried and argon-flushed 10 mL Schlenk tube, $Pd(dba)_2$ (1.2 eq., 1 mg) and the ligand **L2** (1.2 eq., 1.4 mg) were suspended in 300 µL anhydrous ACN and stirred in a pre-heated oil bath at 60°C for 15 min. After cooling down to 40 °C, the farnesylation reagent **Rc** (1.2 eq., 0.7 mg) was added to the yellow solution to obtain the catalyst stock-solution.

In a second argon-flushed 10 mL Schlenk tube, the desired protein (0.25 µmol, UBL3: 3.2 mg, Hsp27: 5.7 mg) was dissolved in 950 µL degassed H_2O. 50 µL of the catalyst stock solution were added and the reaction mixture was stirred at 40 °C and the reaction was monitored by HPLC-MS analysis at different time points.

Dialysis of UBL3 and Hsp27

<u>Dialysis of unmodified UBL3:</u>

UBL3 was incubated with catalyst solution (1.2 eq. $Pd(dba)_2$ and 1.2 eq. **L2**) or with 1.2 eq. **L2** alone in 5 vol% ACN in H_2O for 5 min at 40°C. Afterwards, the solution was centrifuged (10000 rpm, 5 min) to remove precipitated catalyst. The supernatant was dialyzed against 200 volumes of 50 mM phosphate buffer pH 7.0 (KPi) for 16 h using a 3 kDa cut-off membrane and the concentration was determined by NanoDrop.

<u>Two-step dialysis of alkyne-tagged UBL3 and Hsp27:</u>

After the modification of UBL3 and Hsp27 with an alkyne functionality, the reaction mixtures were centrifuged (10000 rpm, 5 min). The supernatant was dialyzed against 200 volumes of 5 vol% ACN in H_2O for 16 h, followed by dialysis agvainst 200 volumes of KPi buffer for 16 h using 3 kDa cut-off membranes on both cases.

<u>Incubation of alkyne-tagged UBL3 and Hsp27 with 10 mM DTT or 10 mM EDTA:</u>

After the modification of UBL3 and Hsp27 with an alkyne functionality, the reaction mixtures were centrifuged (10000 rpm, 5 min). The supernatant was incubated with 10 mM DTT (in H_2O) or 10 mM EDTA (in H_2O) for 1 h and the precipitate was removed by centrifugation (10000 rpm, 10 min). The supernatant was dialyzed against 200 volumes of KPi buffer for 16 h using a 3 kDa cut-off membrane.

Circular Dichroism

For the circular dichroism measurements, a Chirascan plus CD-spectrophotometer (Applied Photophysics) was used. The measurements were performed at 22°C from 200 to 250 nm in 1 nm steps. Each spectrum was obtained by the average of three measurements and substraction of the background. Protein concentrations between 0.1 and 0.5 mg/mL (determined by NanoDrop) were measured in a 1.0 mm microcuvette. The raw data were exported as CSV files and processed using BeStSel[11] and OriginPro.

Pd Content Determination by ICP-MS

Pd content under reaction conditions:

UBL3 (1 eq.) is treated with $Pd(dba)_2$ (1.2 eq.) during allylation (*vide supra*), therefore

$$\text{Pd content (before)} = 1.2 \text{ mol Pd/mol UBL3}$$

Pd content of modified protein:

After purification the Pd content of the modified protein was determined by ICP-MS to be 0.4 µg ($3.76 \cdot 10^{-9}$ mol) per 1 mg alkyne-tagged UBL3 ($7.76 \cdot 10^{-8}$ mol), therefore

$$\text{Pd content (after)} = \frac{3.76 \cdot 10^{-9}}{7.76 \cdot 10^{-8}} = 0.048 \text{ mol Pd/mol UBL3}$$

Pd removal efficiency:

The efficiency of Pd removal during isolation and purification therefore is

$$\text{Pd removal} = \frac{1.2 - 0.048}{1.2} \cdot 100 = 96\%$$

References

[1] S. B. Salunke, N.S. Babu, C.-T. Chen, *Adv. Synth. Catal.* **2011**, *353*, 1234–1240.

[2] J. I. van der Vlugt, A. C. Hewat, S. Neto, R. Sablong, A. M. Mills, M. Lutz, A. L. Spek, C. Müller, D. Vogt, *Adv. Synth. Catal.* **2004**, *346*, 993–1003.

[3] H. W. Zhao, Z. H. Sheng, W. Meng, Y. Y. Yue, H. L. Li, X. Q. Song and Z. Yang, *Synlett*, 2013, 24, 2743–2747.

[4] A. B. Gamble, P. A. Keller, *Chem. Commun.* **2010**, *46*, 4076–4078.

[5] P. Capdevielle and M. Maumy, *Tetrahedron Lett*, 1993, 34, 1007–1010.

[6] M. Ochiai, K. Fukui, S. Iwatsuki, K. Ishihara and K. Matsumoto, *Organometallics*, 2005, 24, 5528–5536.

[7] L. S. Melvin, *Tetrahedron Lett*, 1981, 22, 3375–3376.

[8] G. D. Cuny, S. L. Buchwald, *J. Am. Chem. Soc.* **1993**, *115*, 2066–2068.

[9] T. Schlatzer, J. Kriegesmann, H. Schröder, M. Trobe, C. Lembacher-Fadum, S. Santner, A. V. Kravchuk, C. F. W. Becker and R. Breinbauer, *J. Am. Chem. Soc.* **2019**, *141*, 14931–14937.

[10] S. D. Tilley, M. B. Francis, *J. Am. Chem. Soc.* **2006**, *128*, 1080–1081.

[11] A. Micsonai, F. Wien, L. Kernya, Y. H. Lee, Y. Goto, M. Réfrégiers and J. Kardos, *Proc. Natl. Acad. Sci.* **2015**, *112*, E3095–E3103.

NMR-Spectra

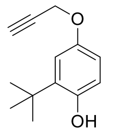

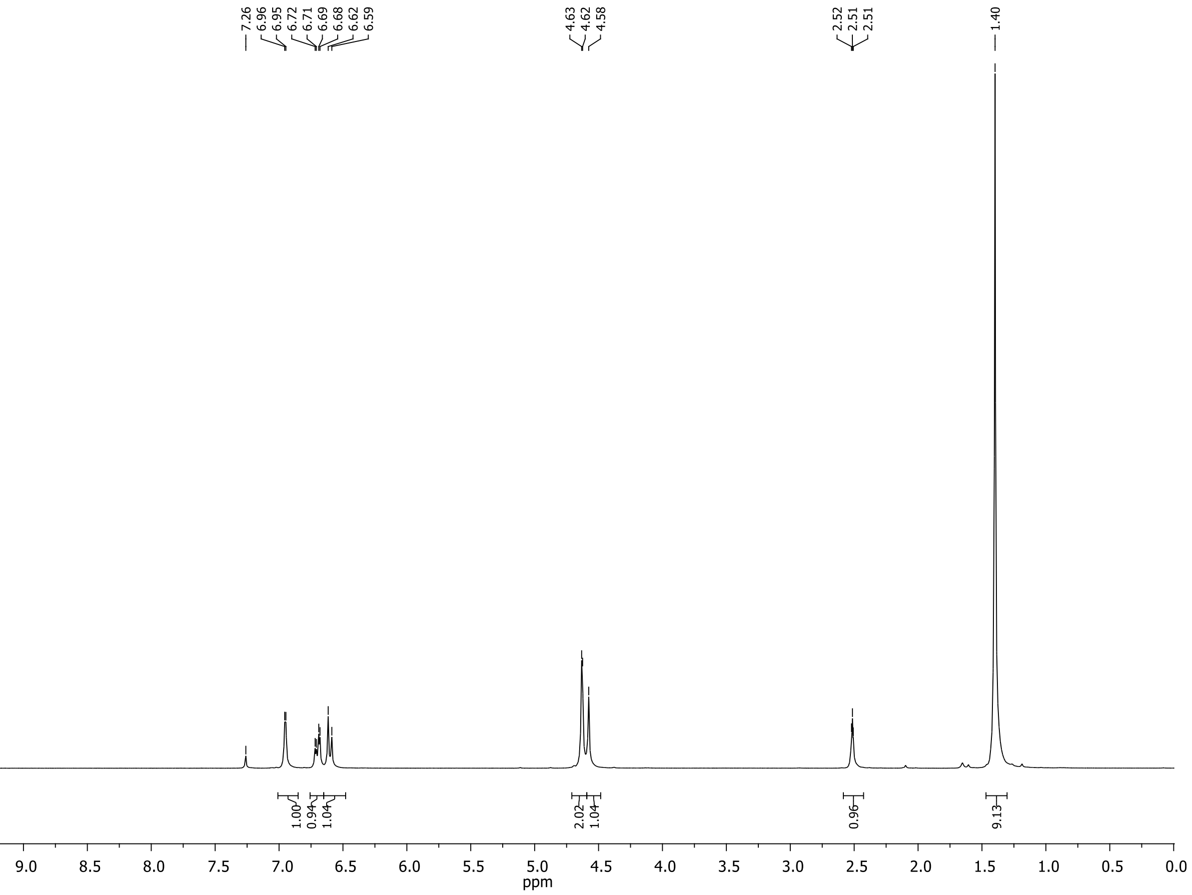

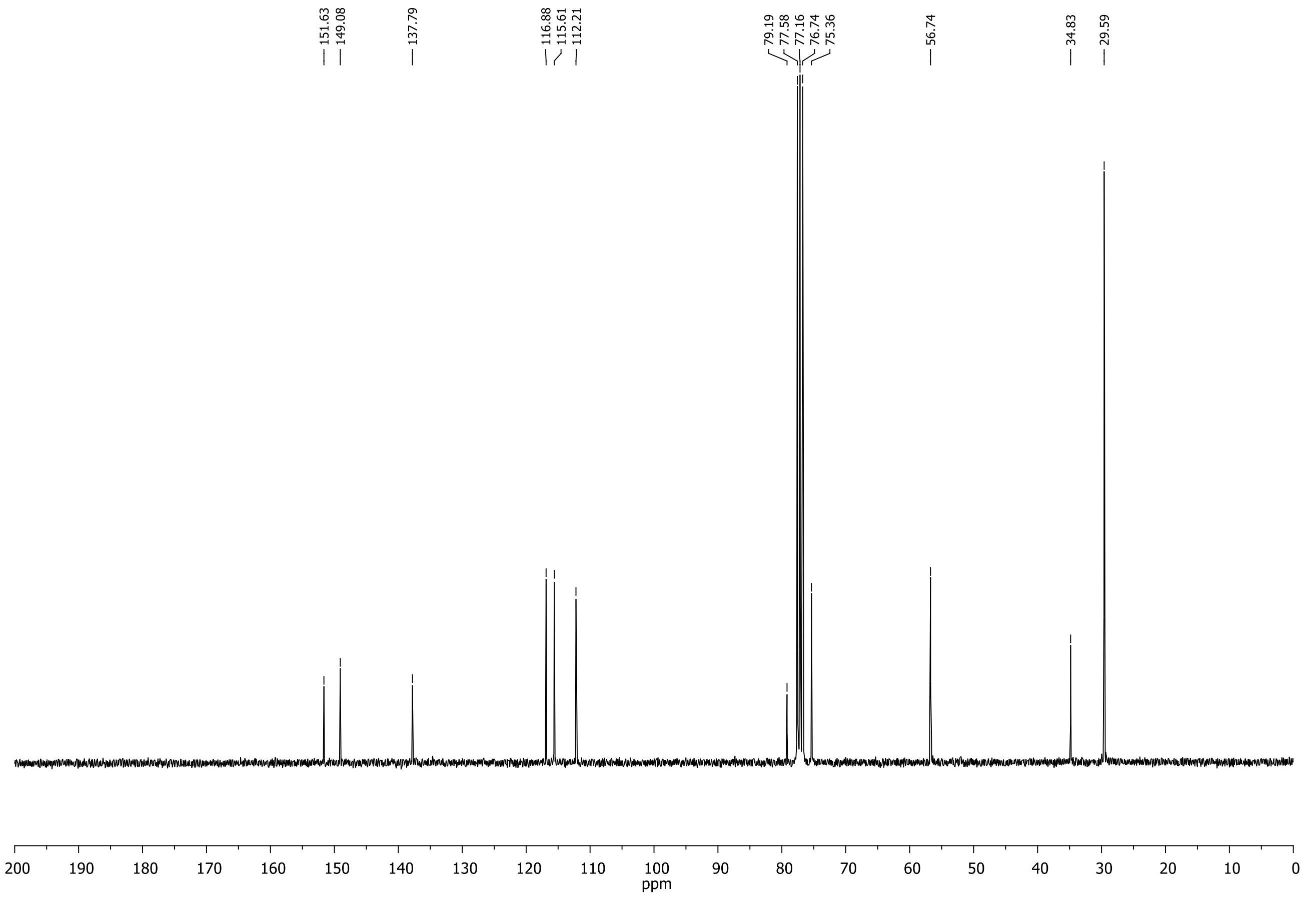

Compound **S1**: ^{1}H-NMR (300 MHz, $CDCl_3$); ^{13}C-NMR (76 MHz, $CDCl_3$)

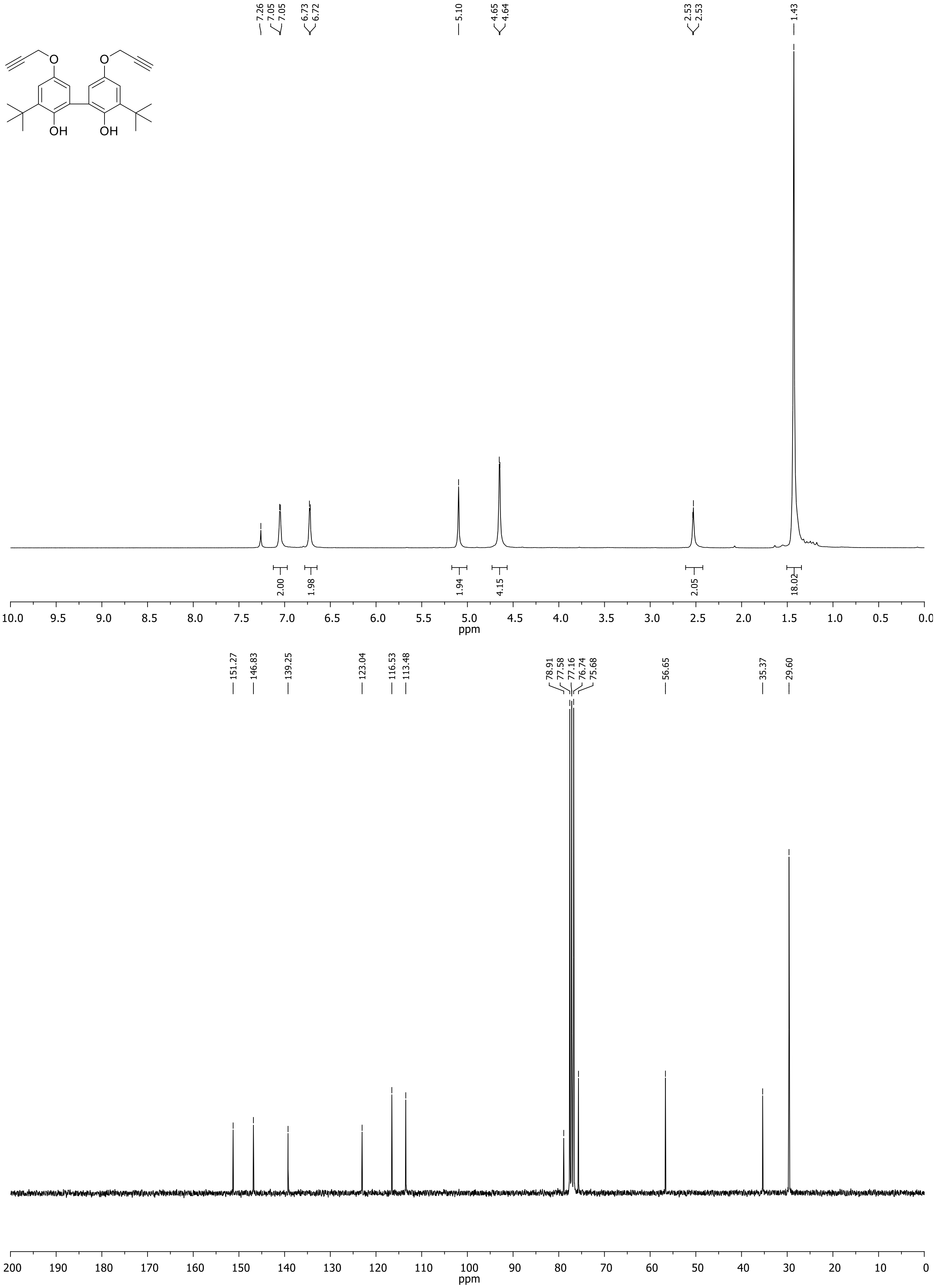

Compound **S2**: ^{1}H-NMR (300 MHz, $CDCl_3$); ^{13}C-NMR (76 MHz, $CDCl_3$)

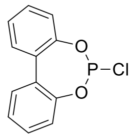

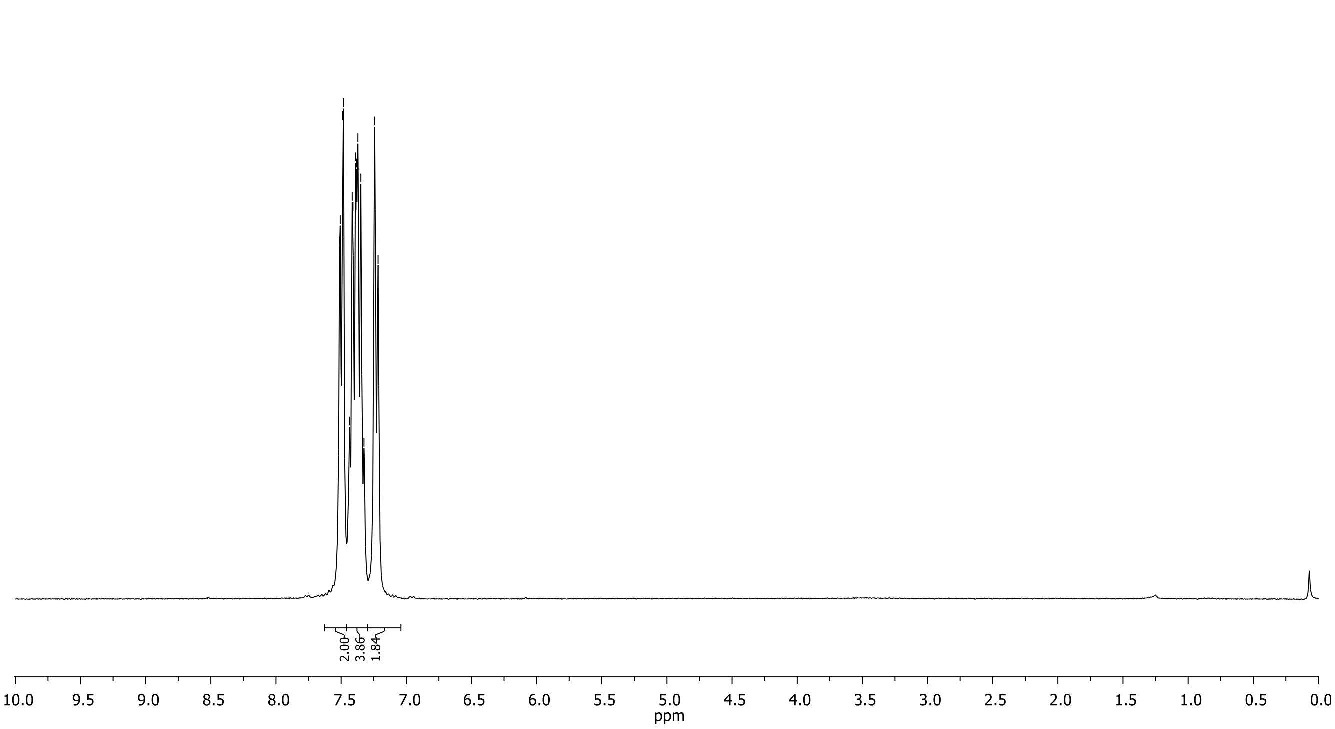

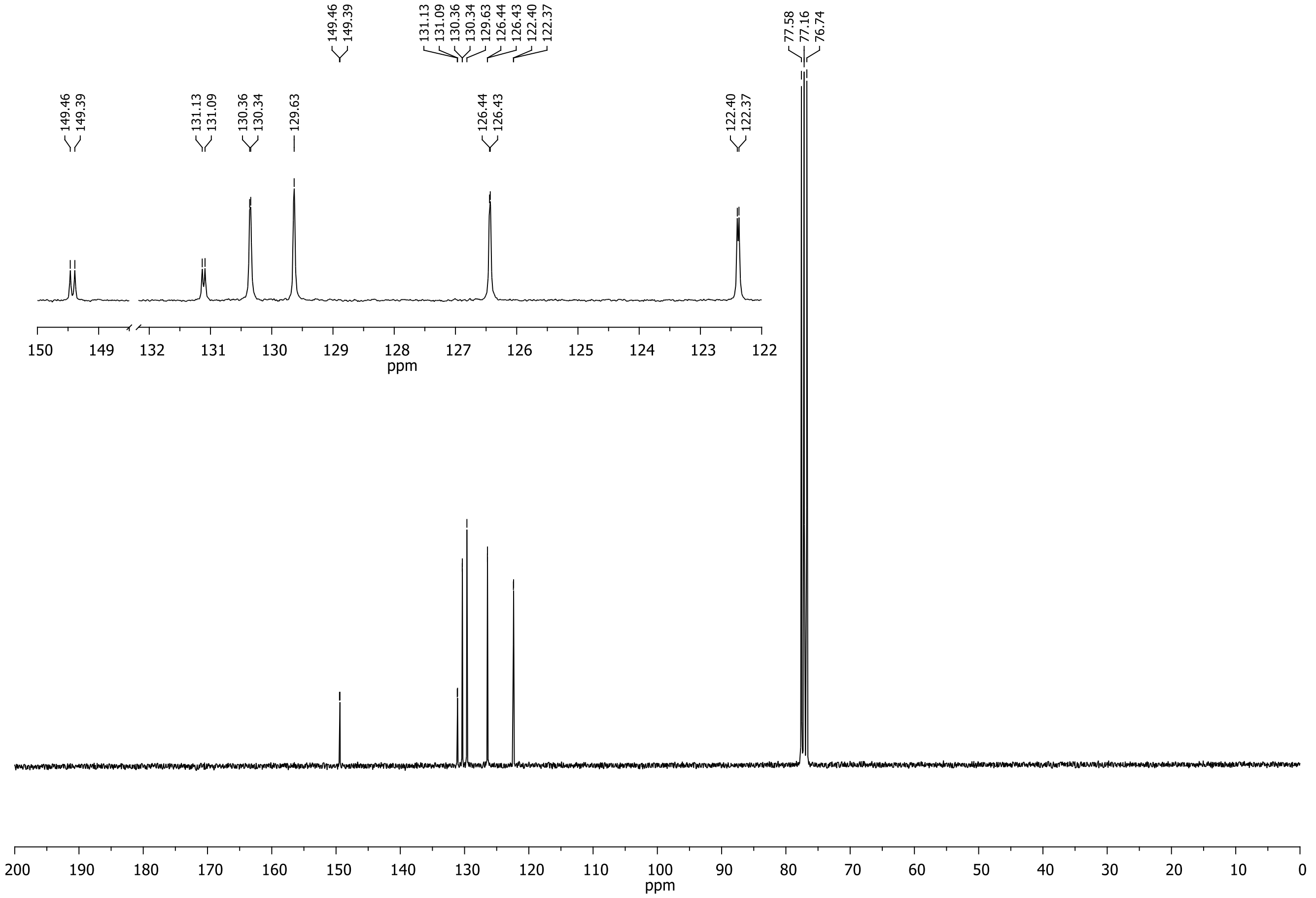

Compound **6**: ^{1}H-NMR (300 MHz, $CDCl_3$); ^{13}C-NMR (76 MHz, $CDCl_3$)

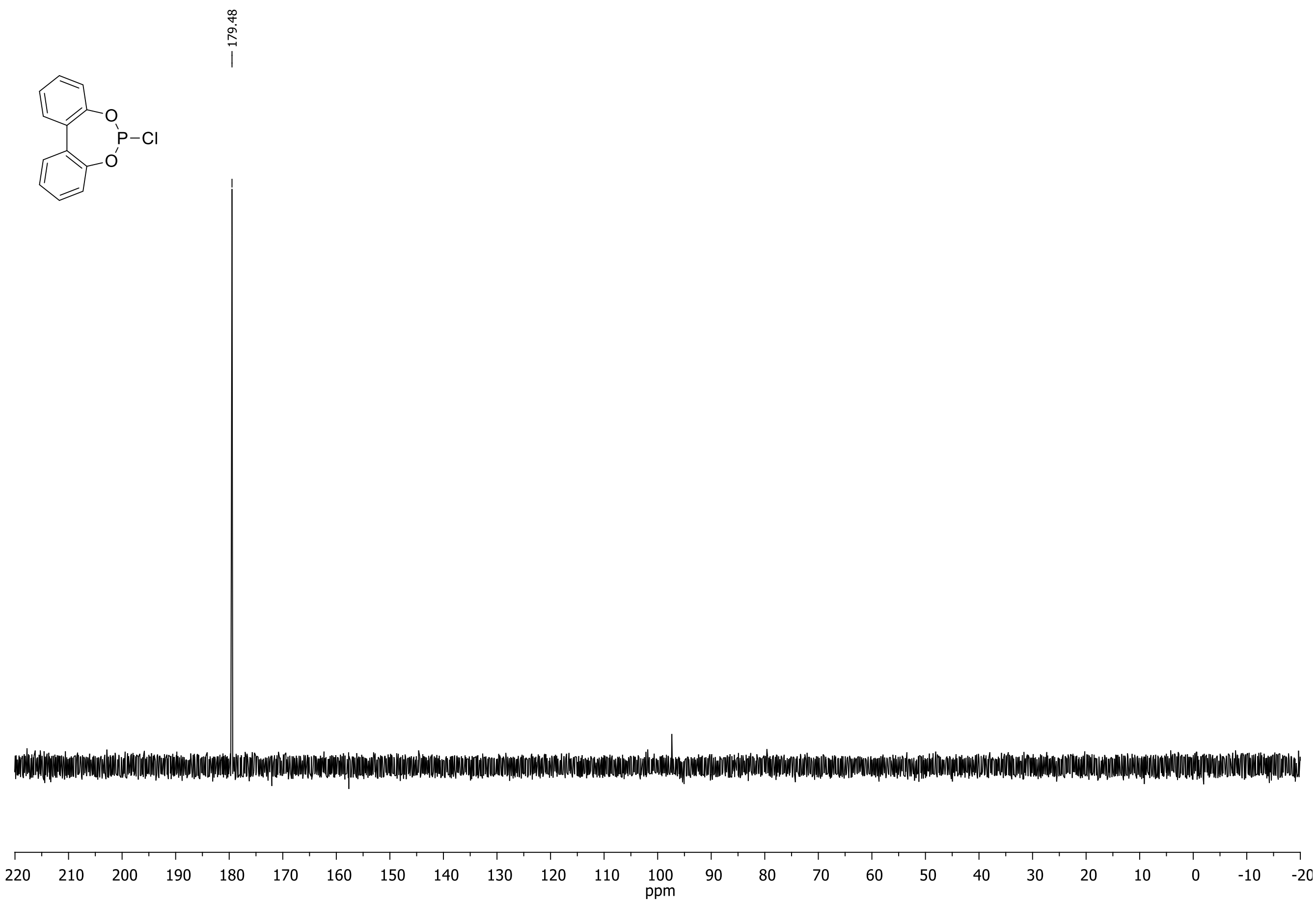

Compound **6**: ^{31}P-NMR (122 MHz, $CDCl_3$)

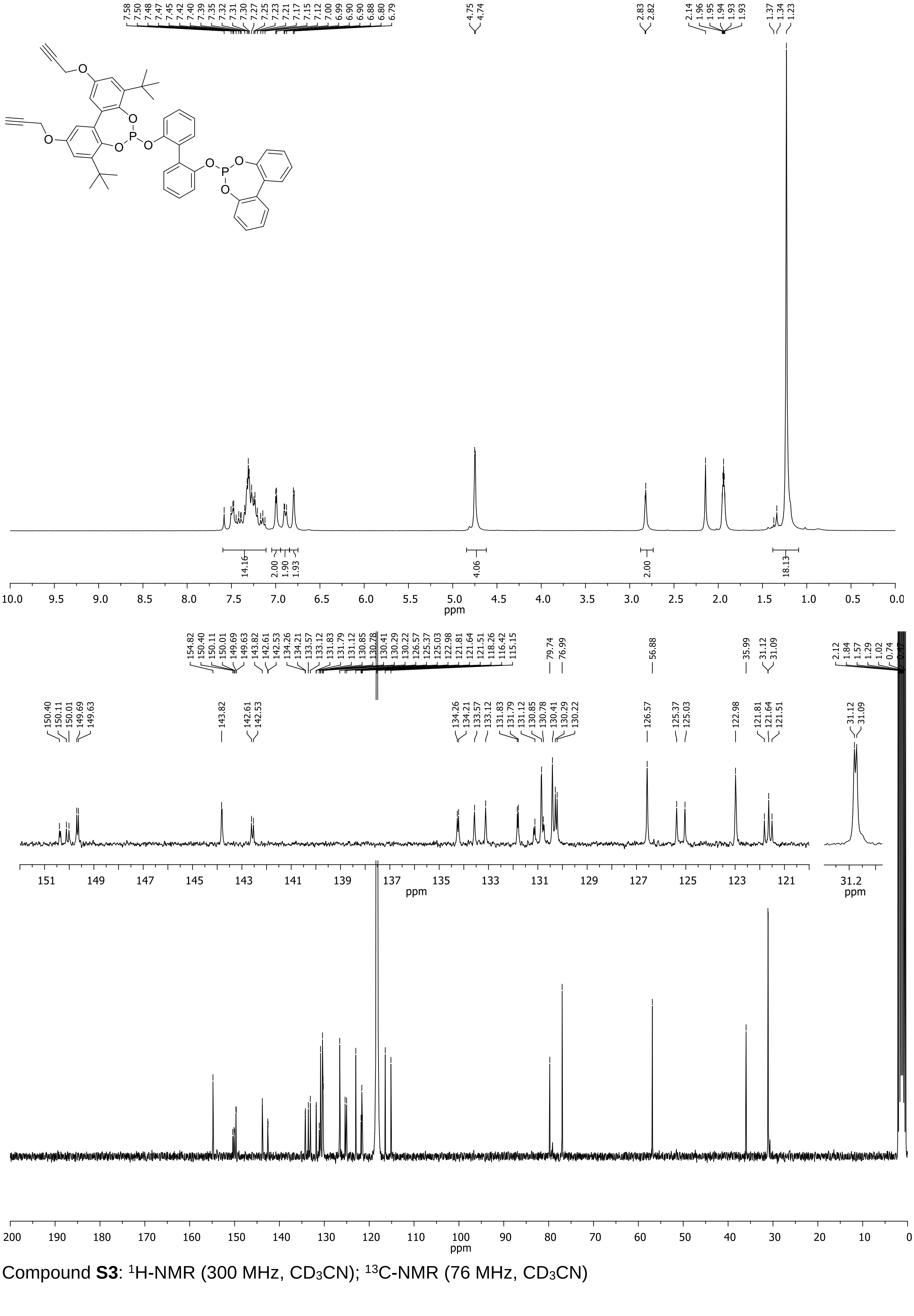

Compound **S3**: ^{1}H-NMR (300 MHz, CD_3CN); ^{13}C-NMR (76 MHz, CD_3CN)

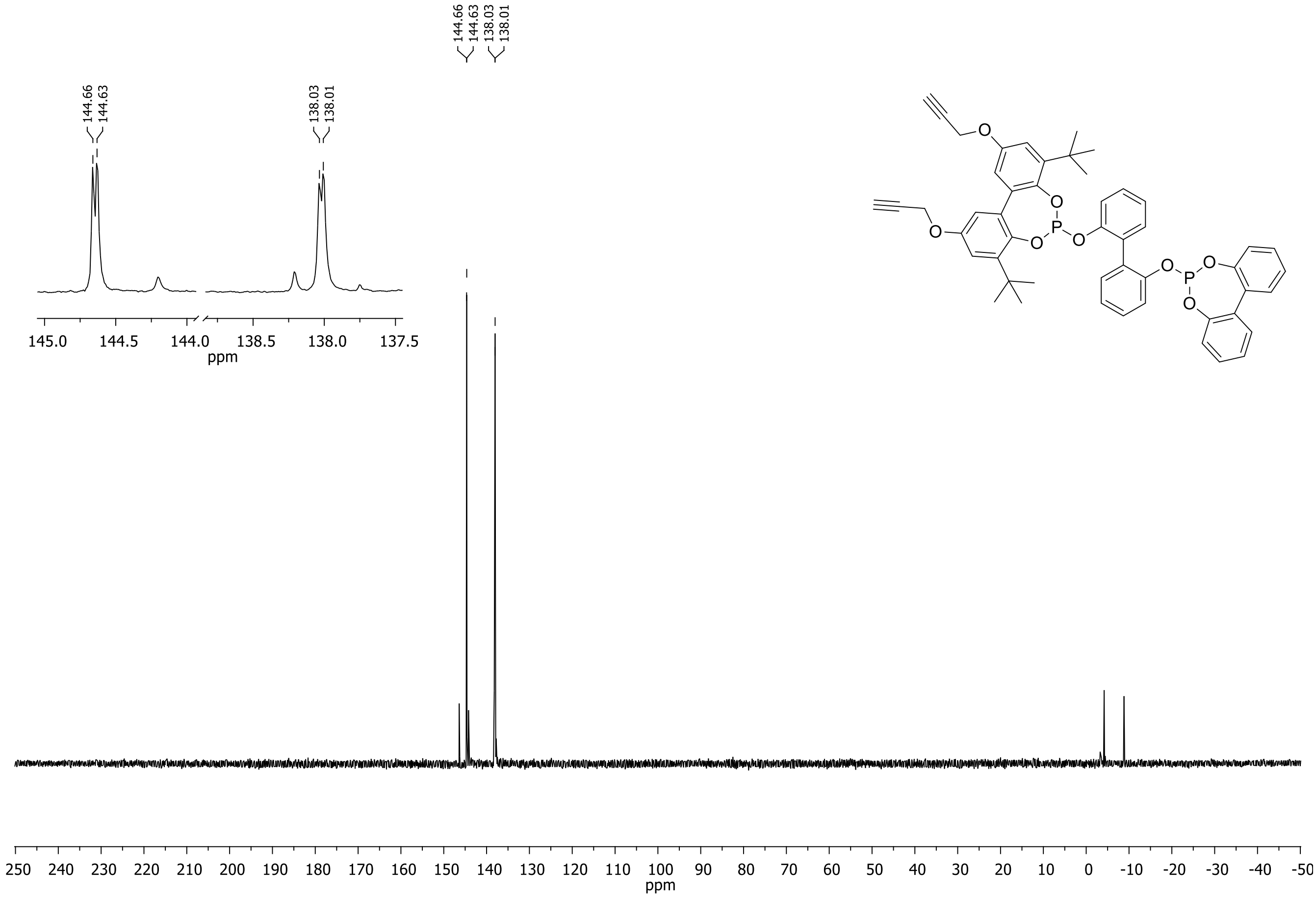

Compound **S3**: ^{31}P-NMR (122 MHz, CD_3CN)

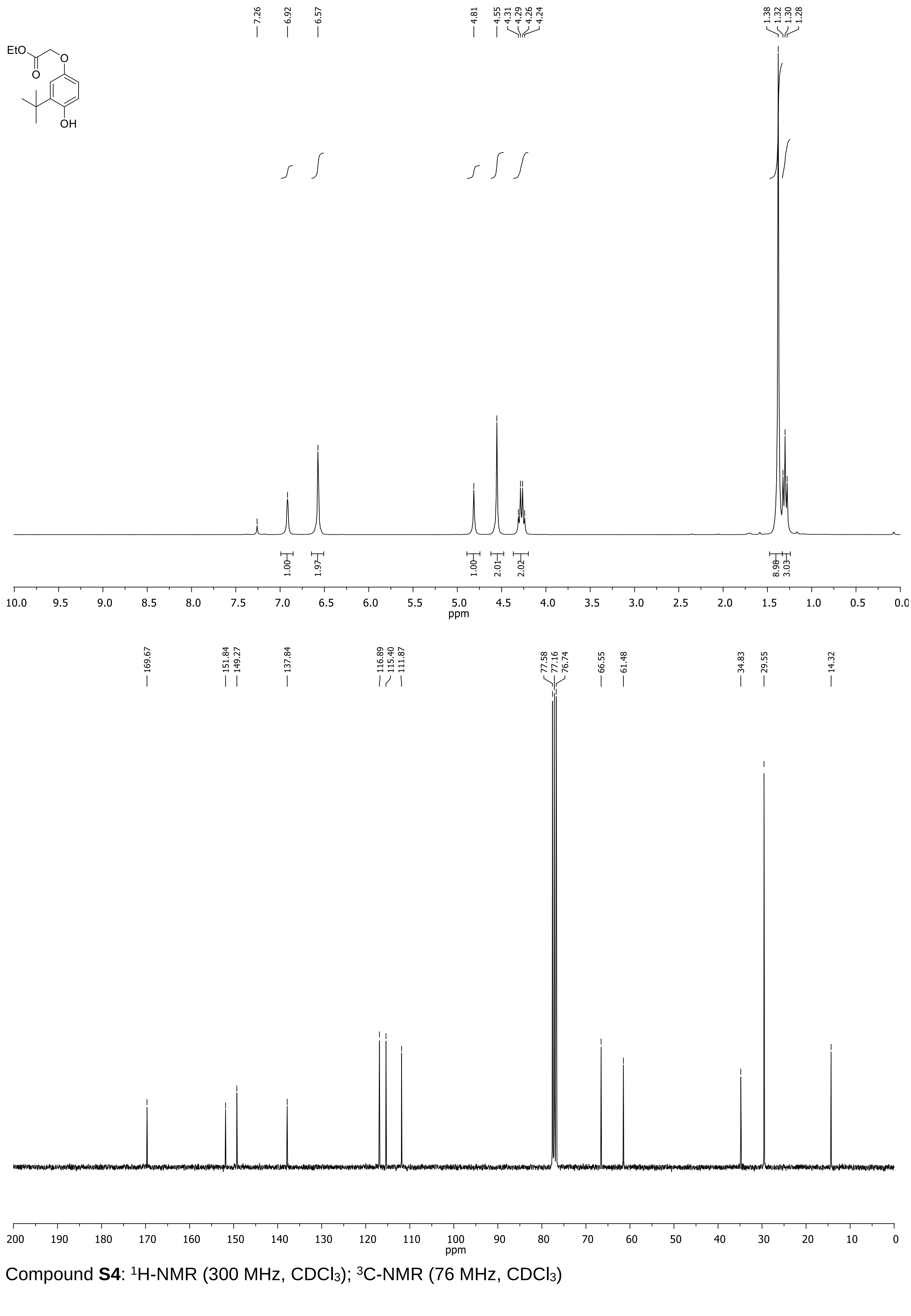

Compound **S4**: ^{1}H-NMR (300 MHz, $CDCl_3$); ^{3}C-NMR (76 MHz, $CDCl_3$)

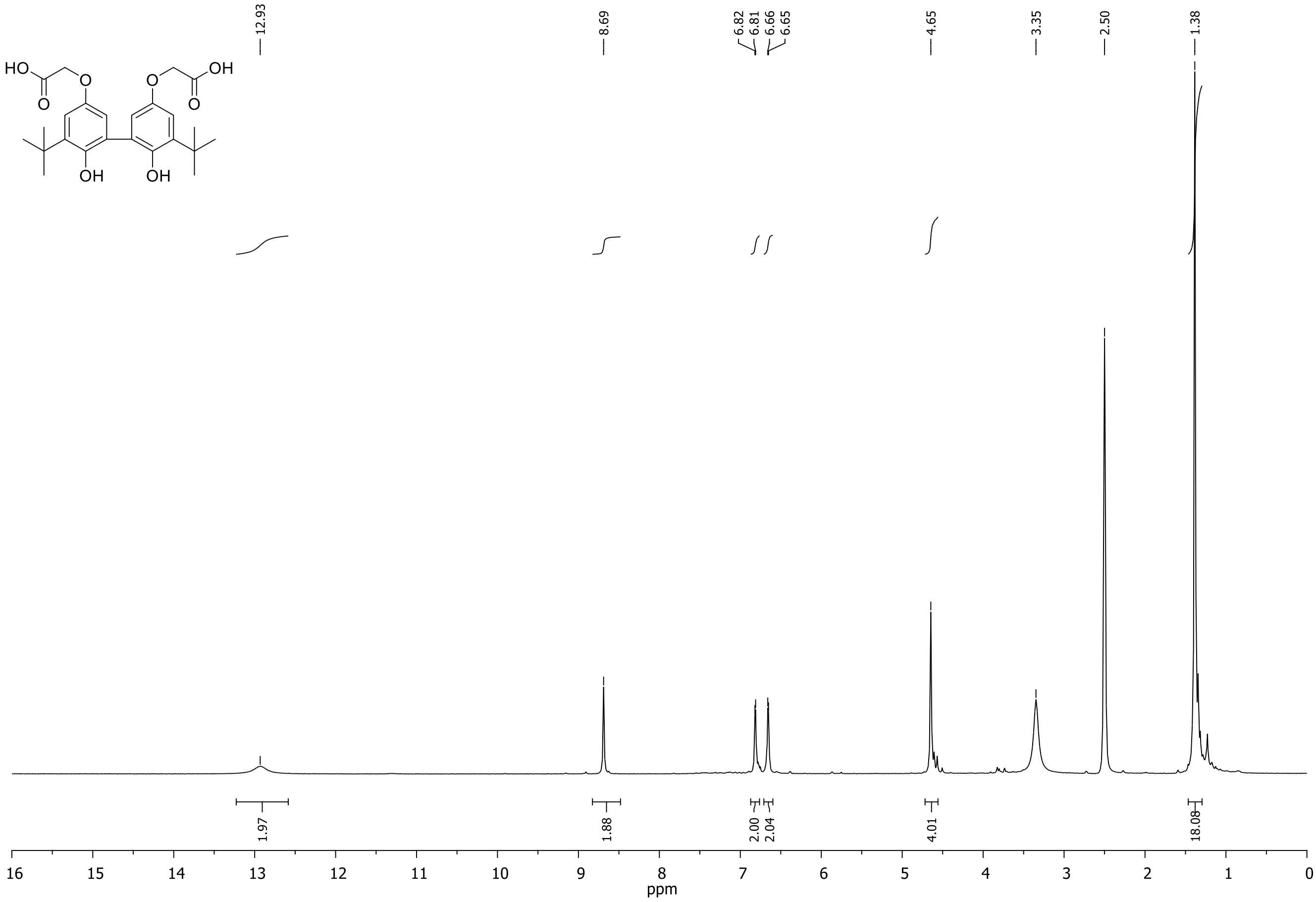

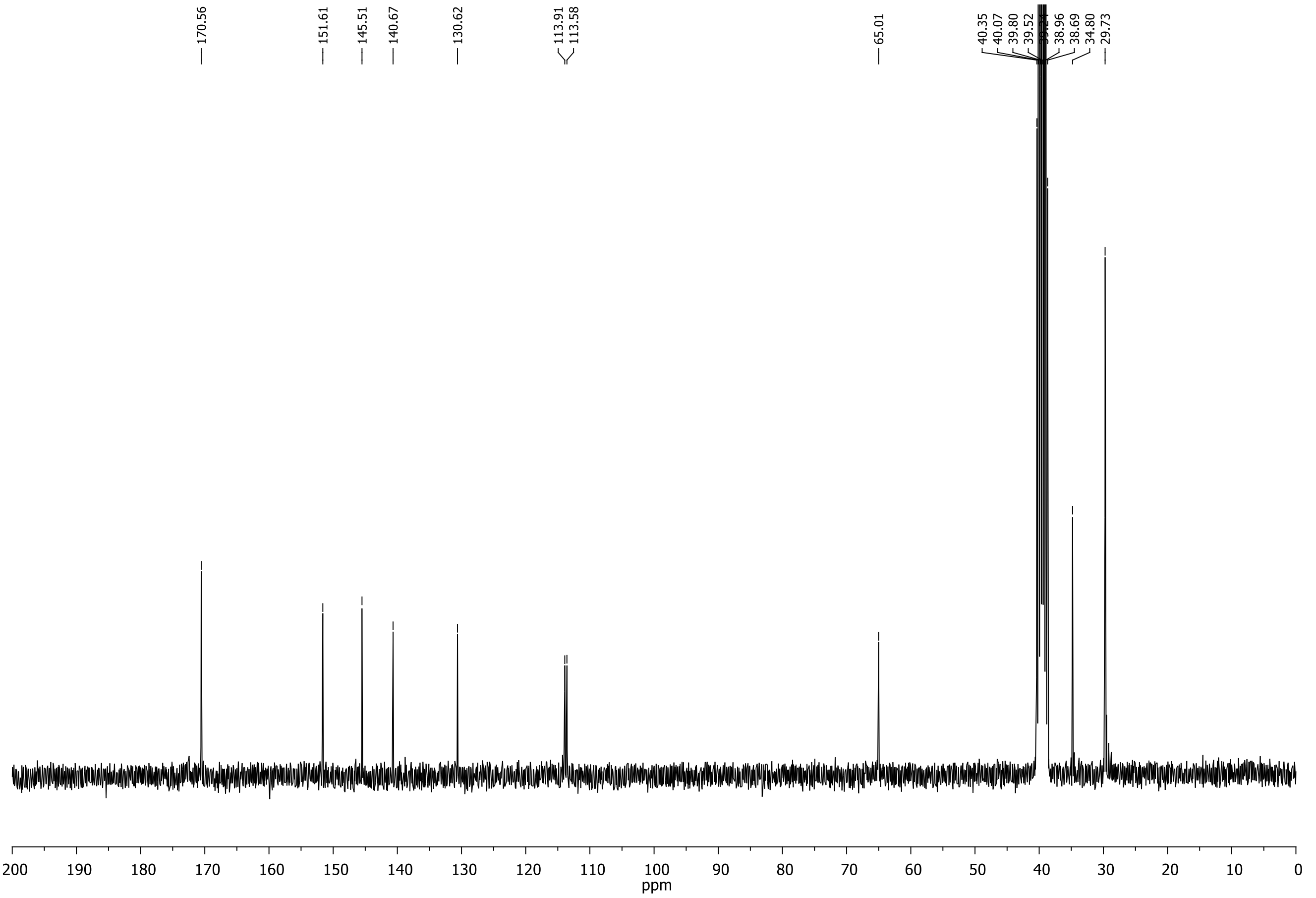

Compound **S5**: ^{1}H-NMR (300 MHz, DMSO-d_6); ^{13}C-NMR (76 MHz, DMSO-d_6)

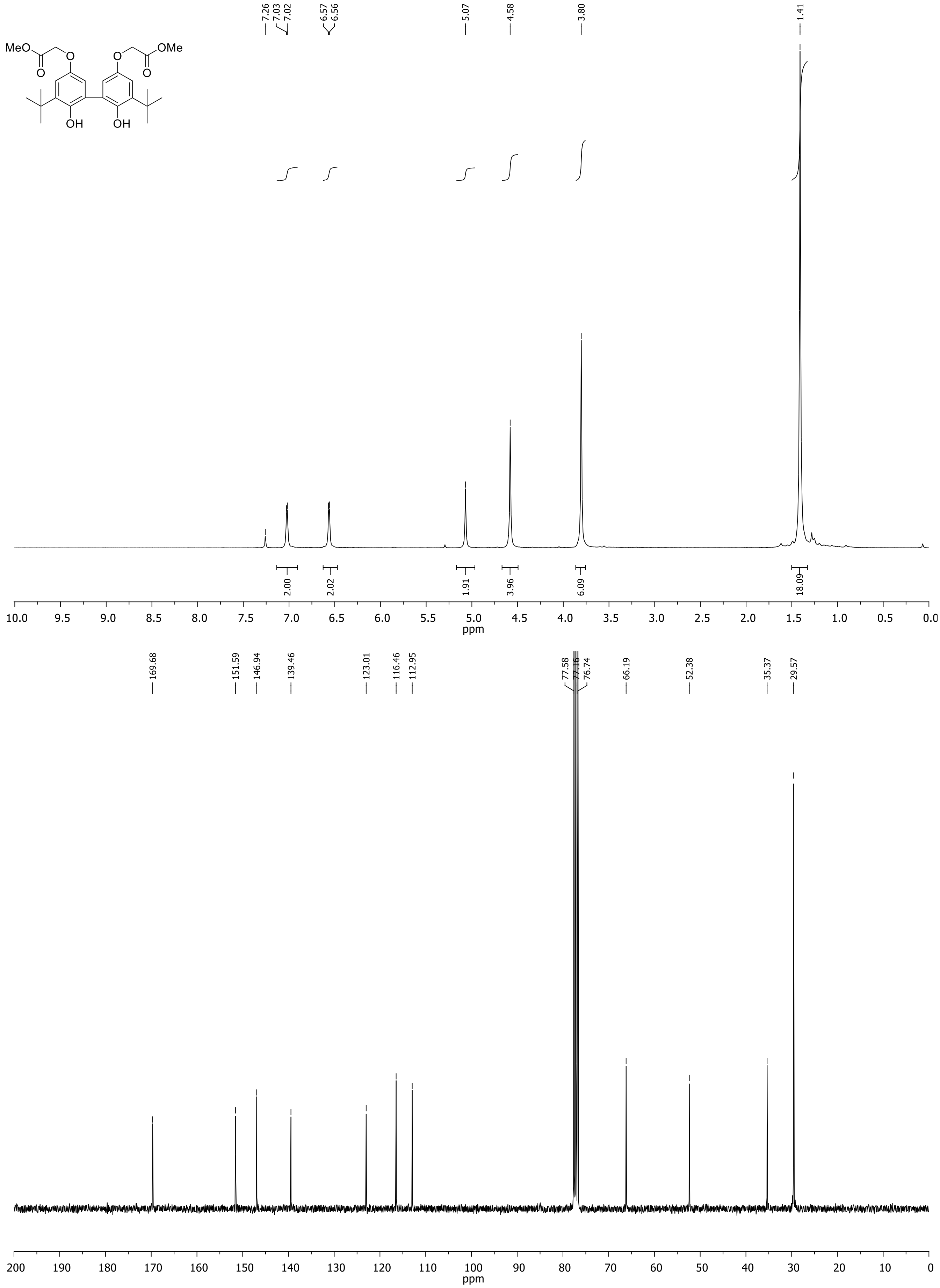

Compound **S6**: ^{1}H-NMR (300 MHz, $CDCl_3$); ^{13}C-NMR (76 MHz, $CDCl_3$)

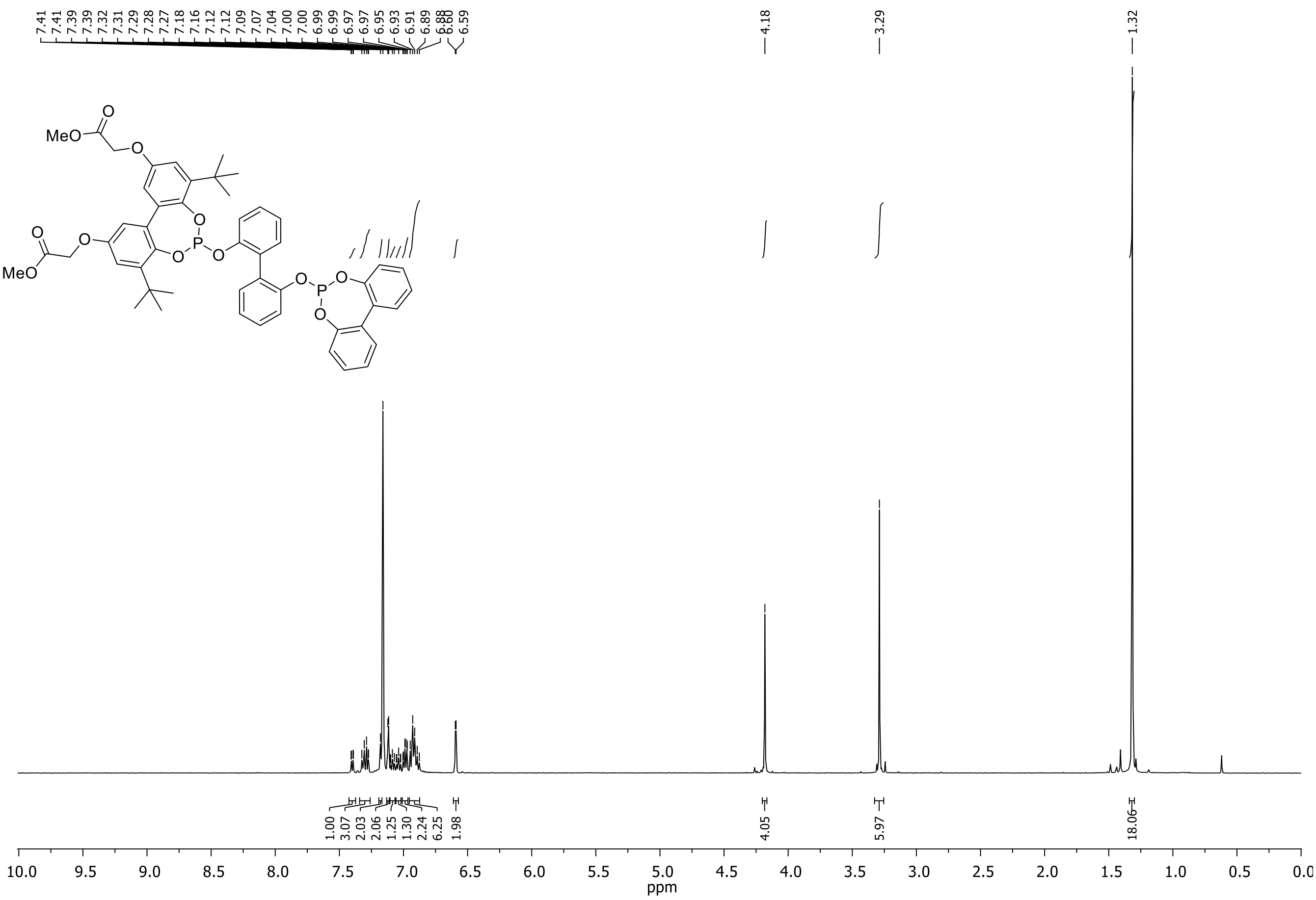

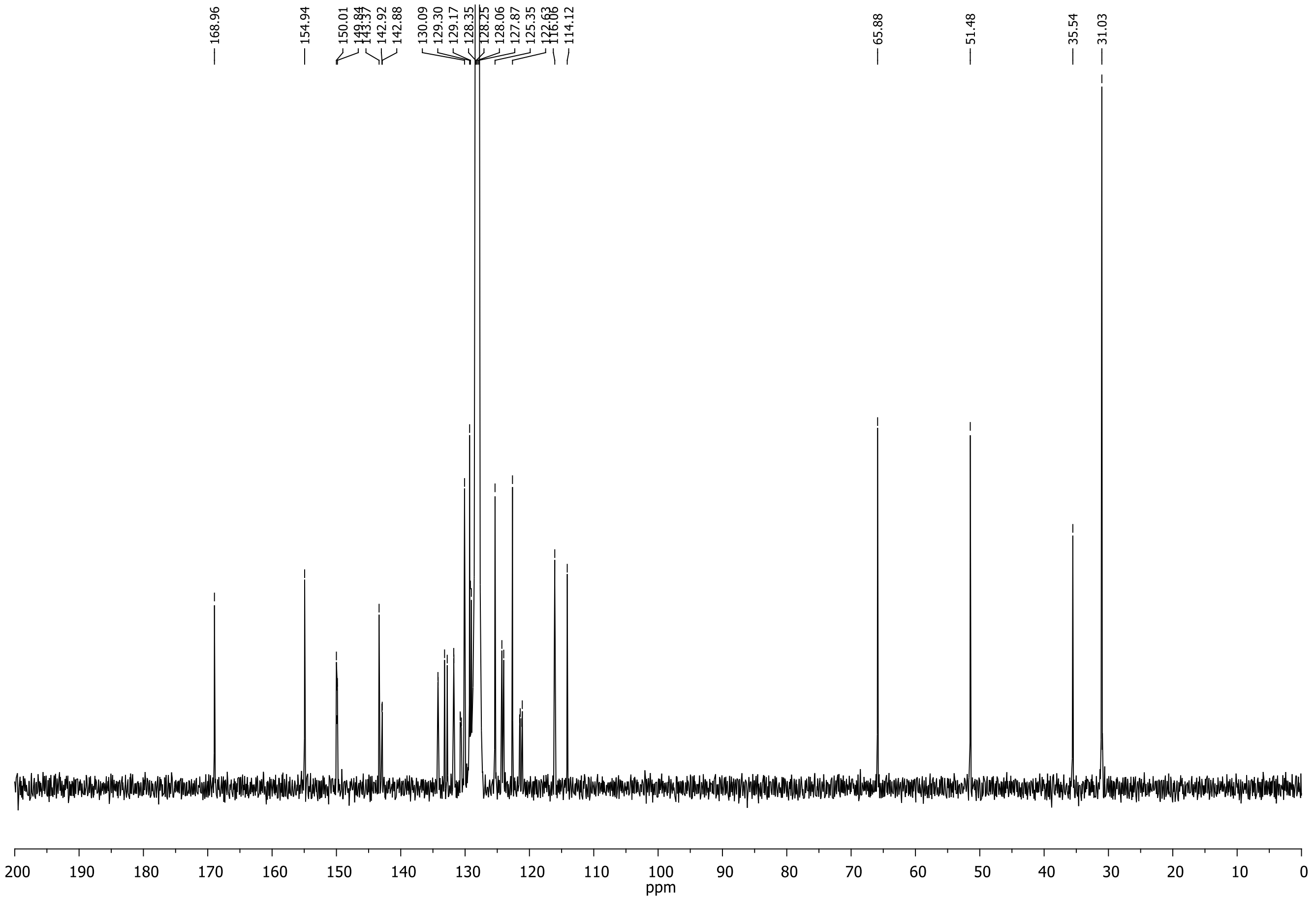

Compound **S7**: ^{1}H-NMR (500 MHz, C_6D_6); ^{13}C-NMR (126 MHz, C_6D_6)

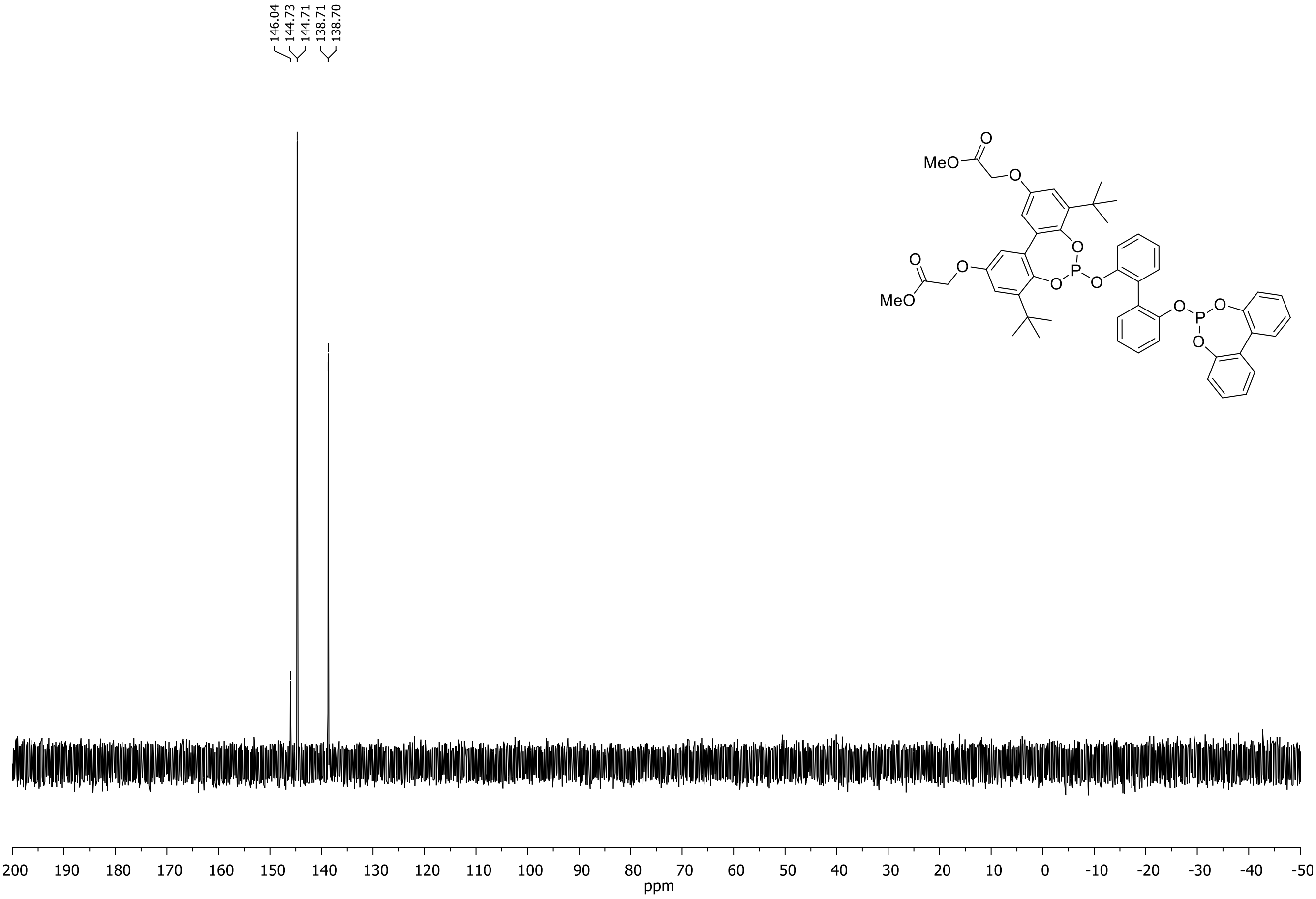

Compound **S7**: ^{31}P-NMR (202 MHz, C_6D_6)

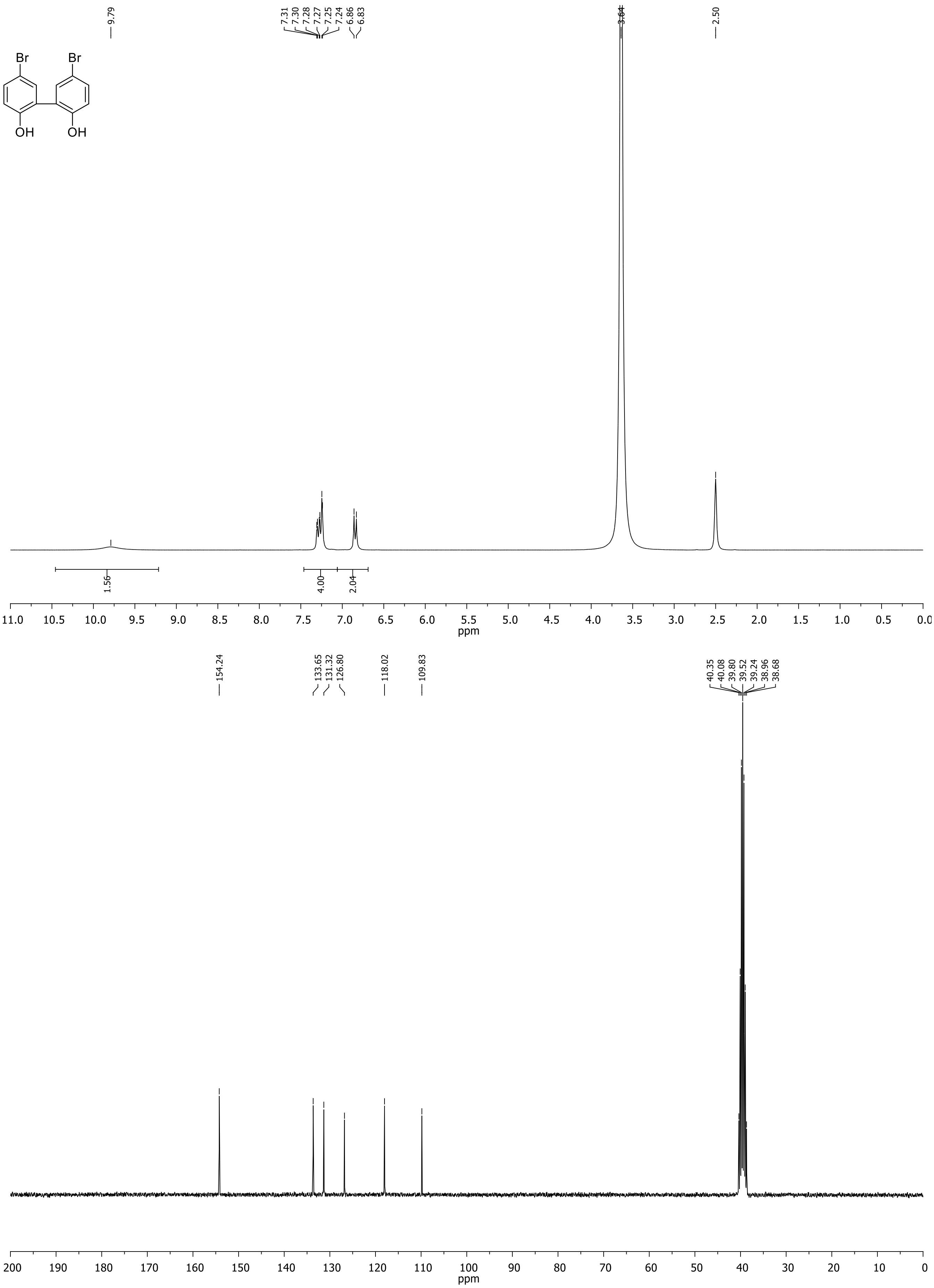

Compound **2**: ^{1}H-NMR (300 MHz, DMSO-d_6); ^{13}C-NMR: (76 MHz, DMSO-d_6)

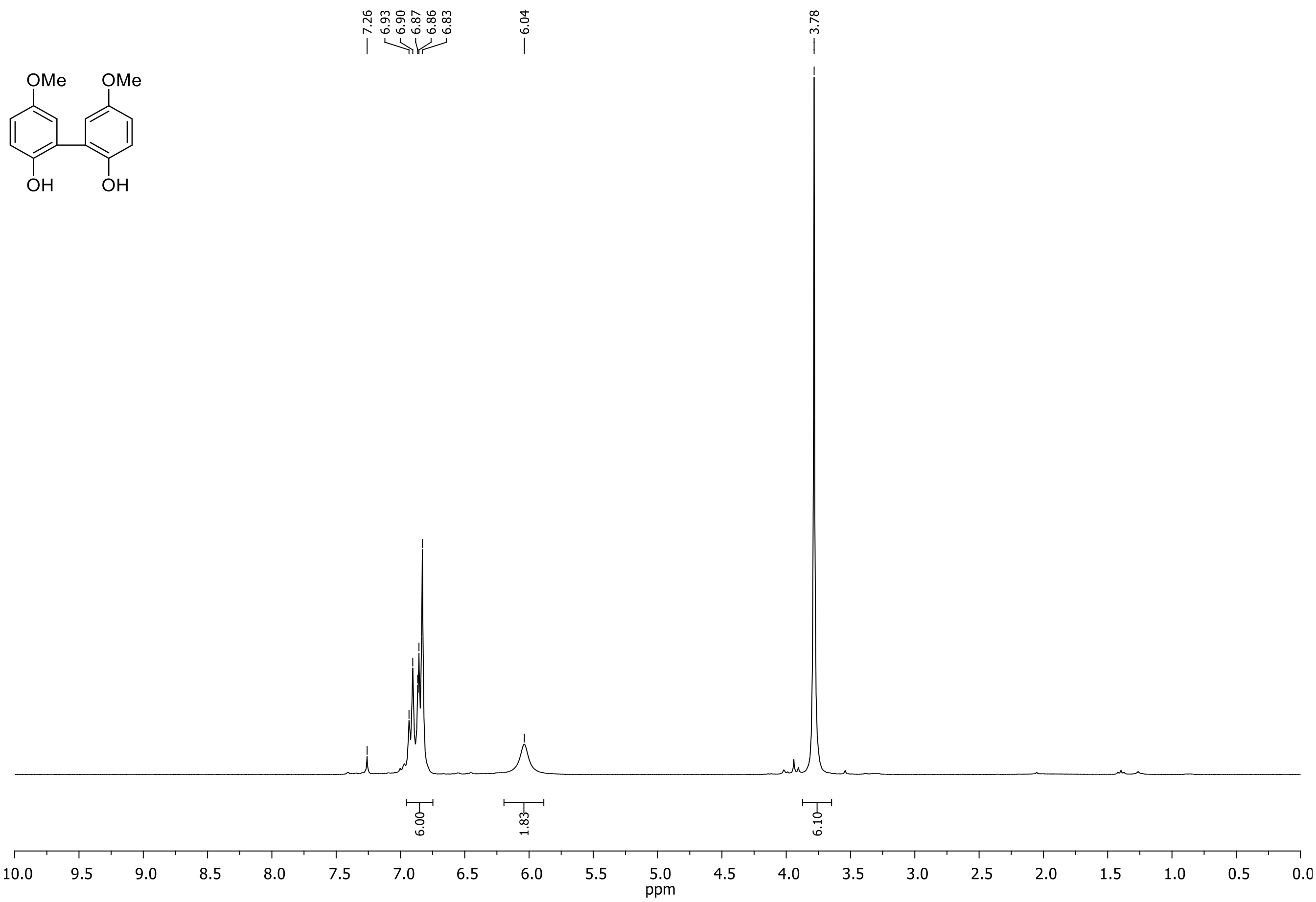

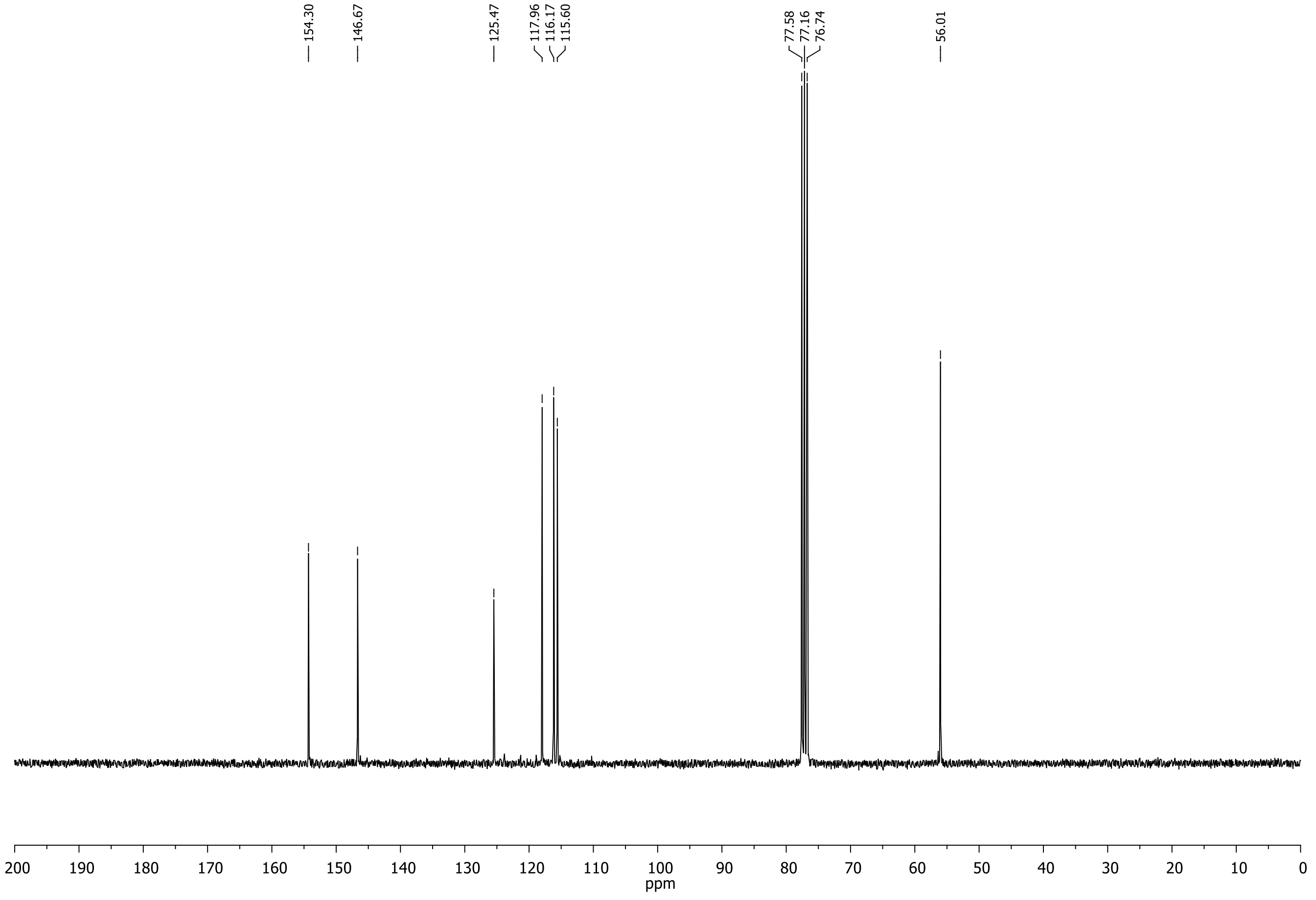

Compound **3**: ^{1}H-NMR (300 MHz, $CDCl_3$); ^{13}C-NMR: (76 MHz, $CDCl_3$)

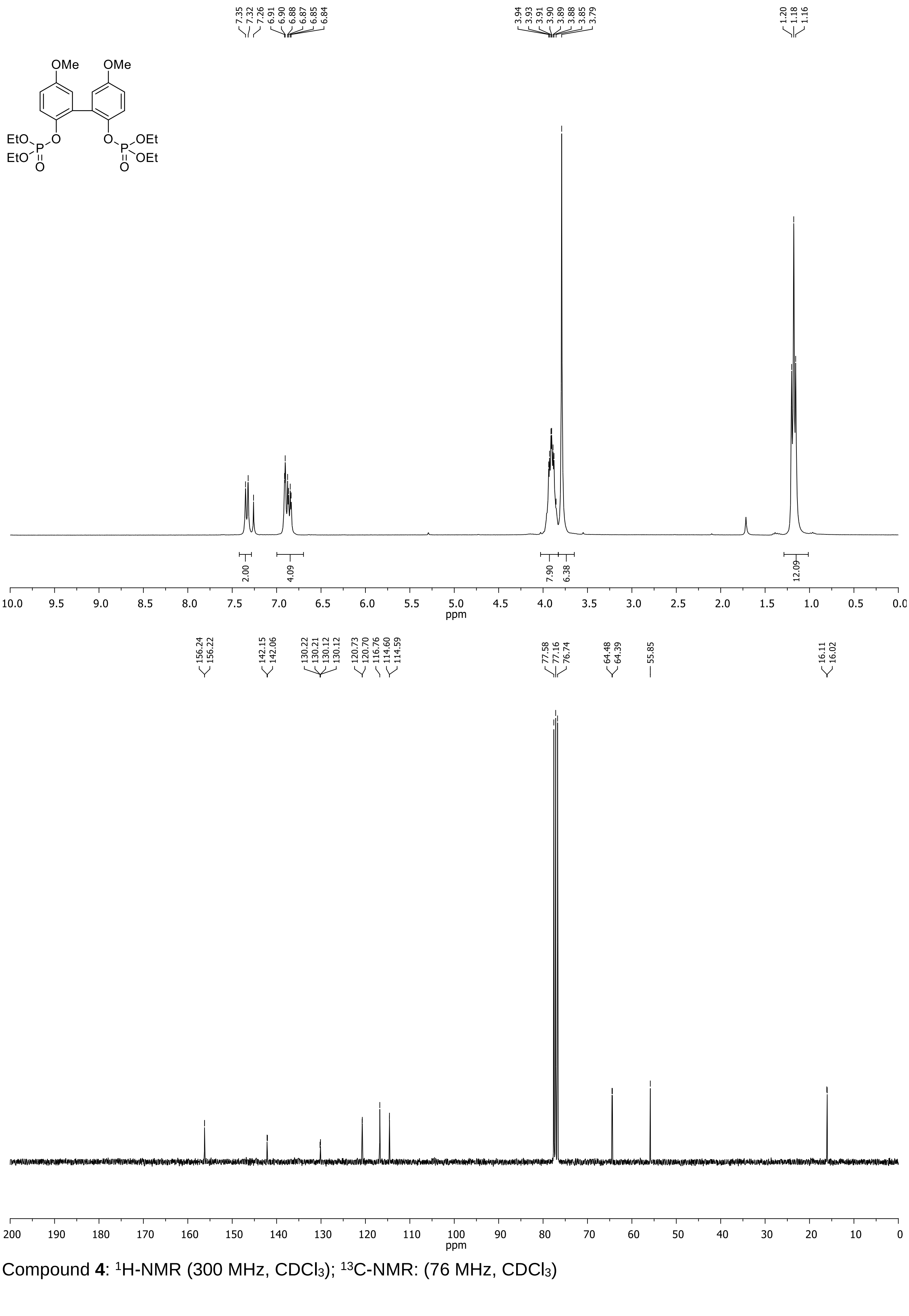

Compound **4**: ^{1}H-NMR (300 MHz, $CDCl_3$); ^{13}C-NMR: (76 MHz, $CDCl_3$)

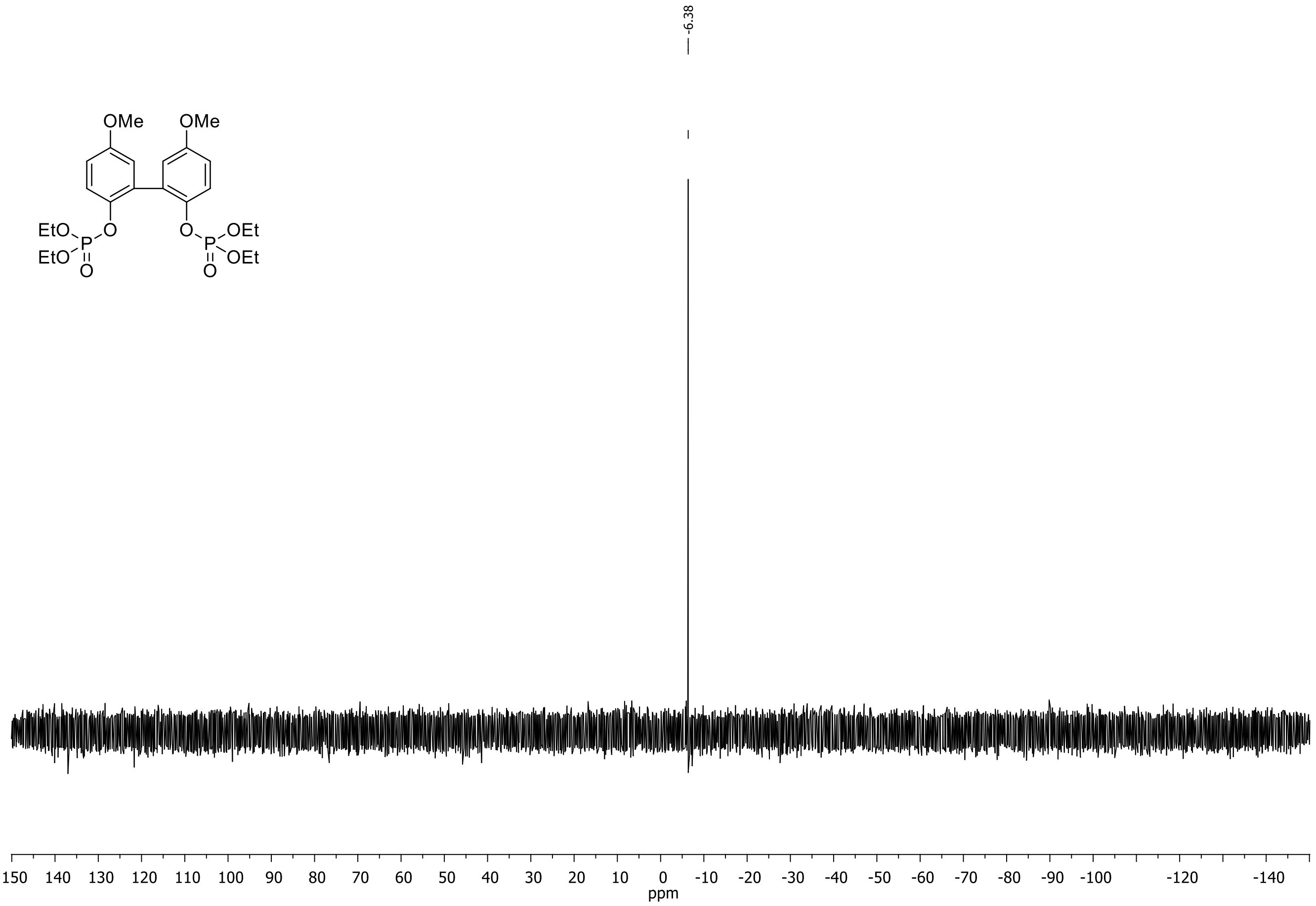

Compound **4**: ^{31}P-NMR (81 MHz, $CDCl_3$)

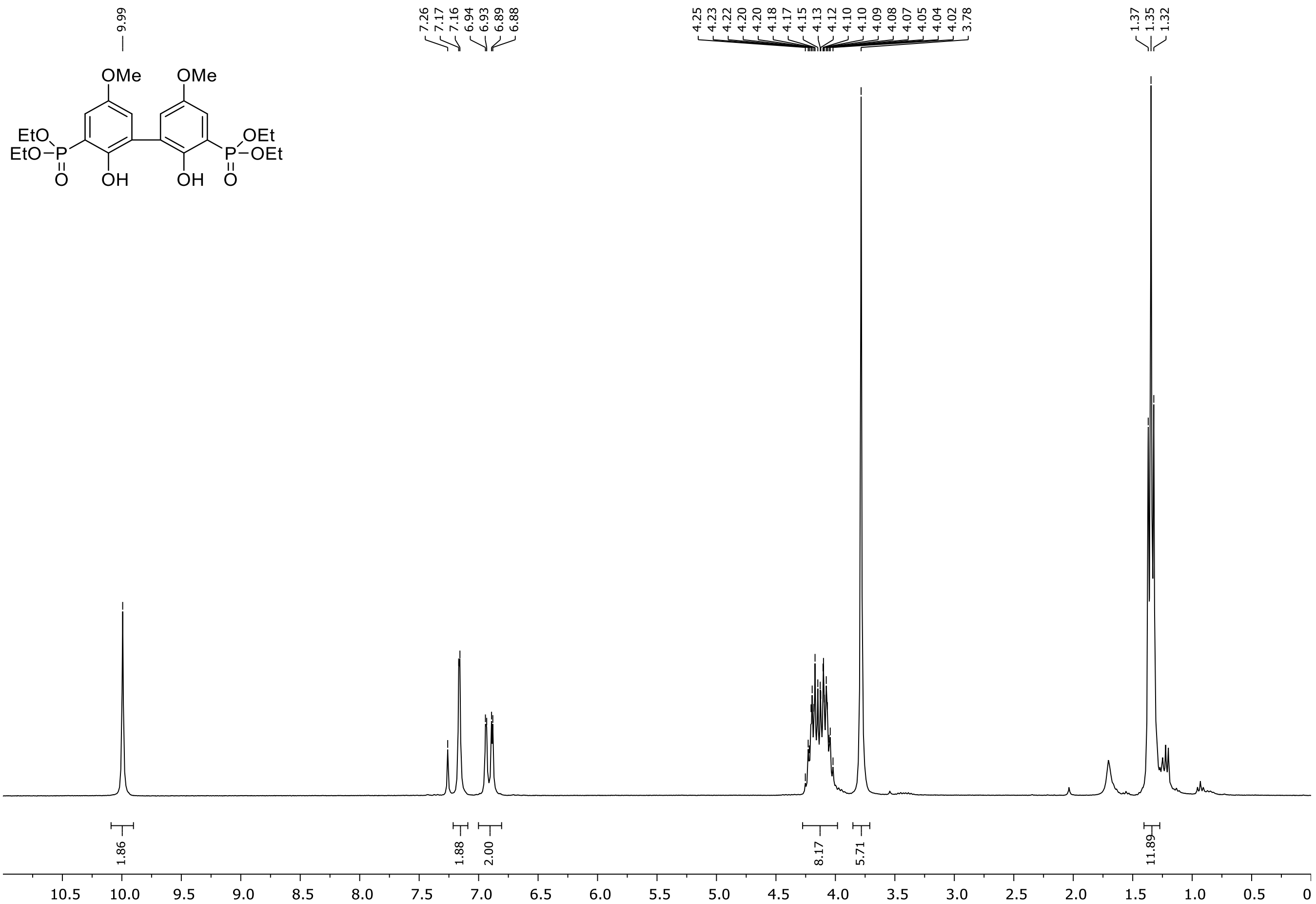

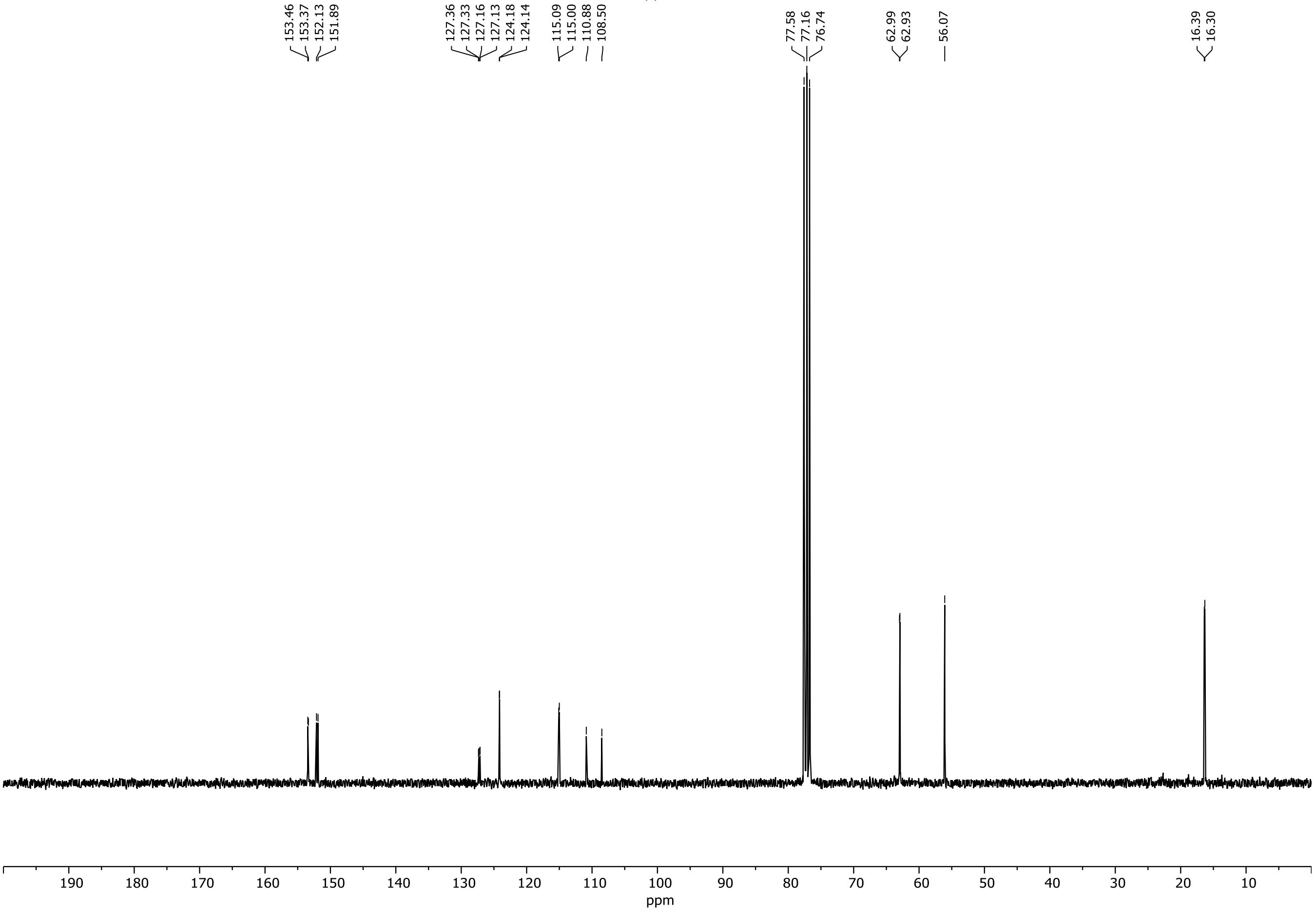

Compound **5**: ^{1}H-NMR (300 MHz, $CDCl_3$); ^{13}C-NMR: (76 MHz, $CDCl_3$)

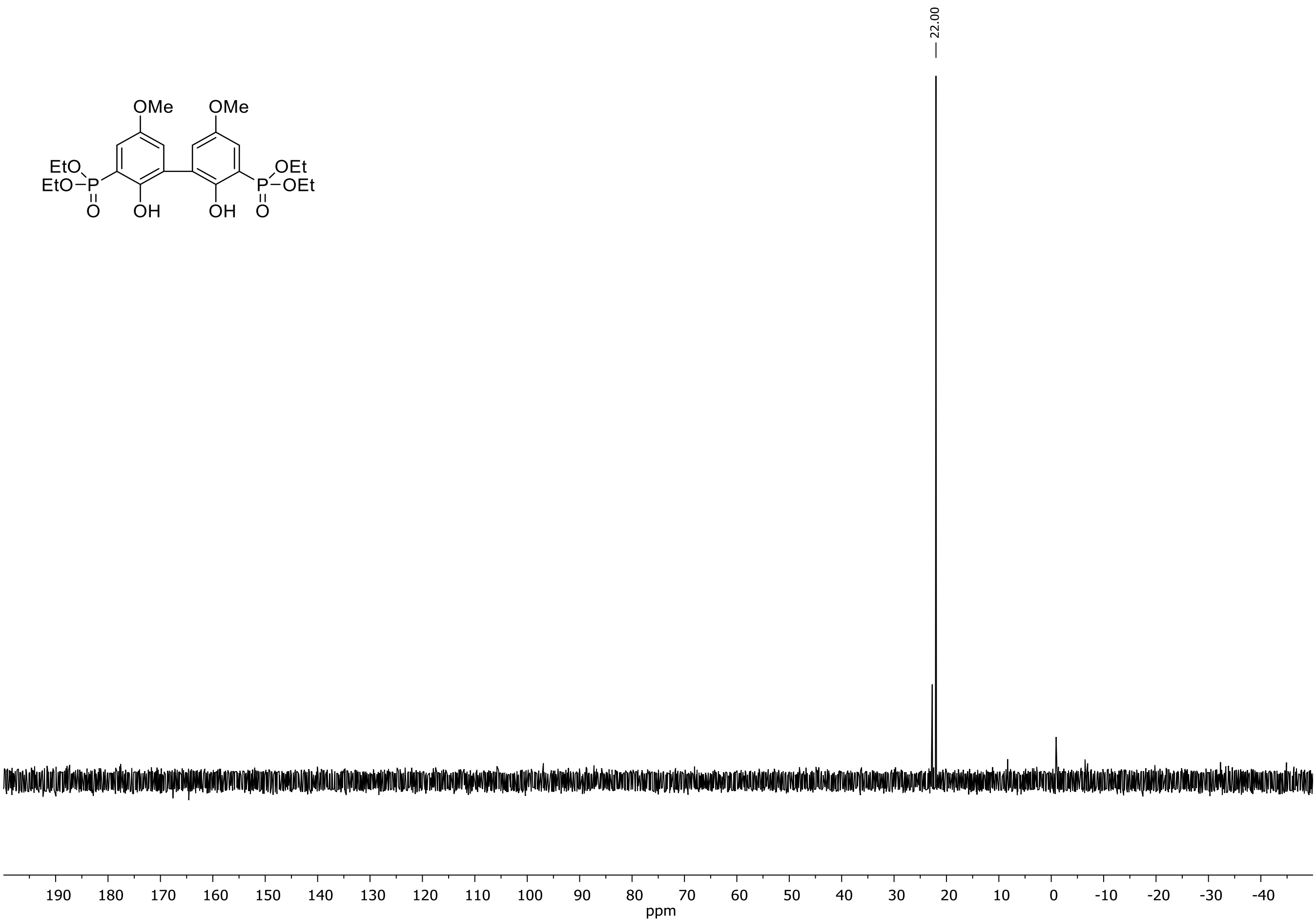

Compound **5**: ^{31}P-NMR (202 MHz, $CDCl_3$)

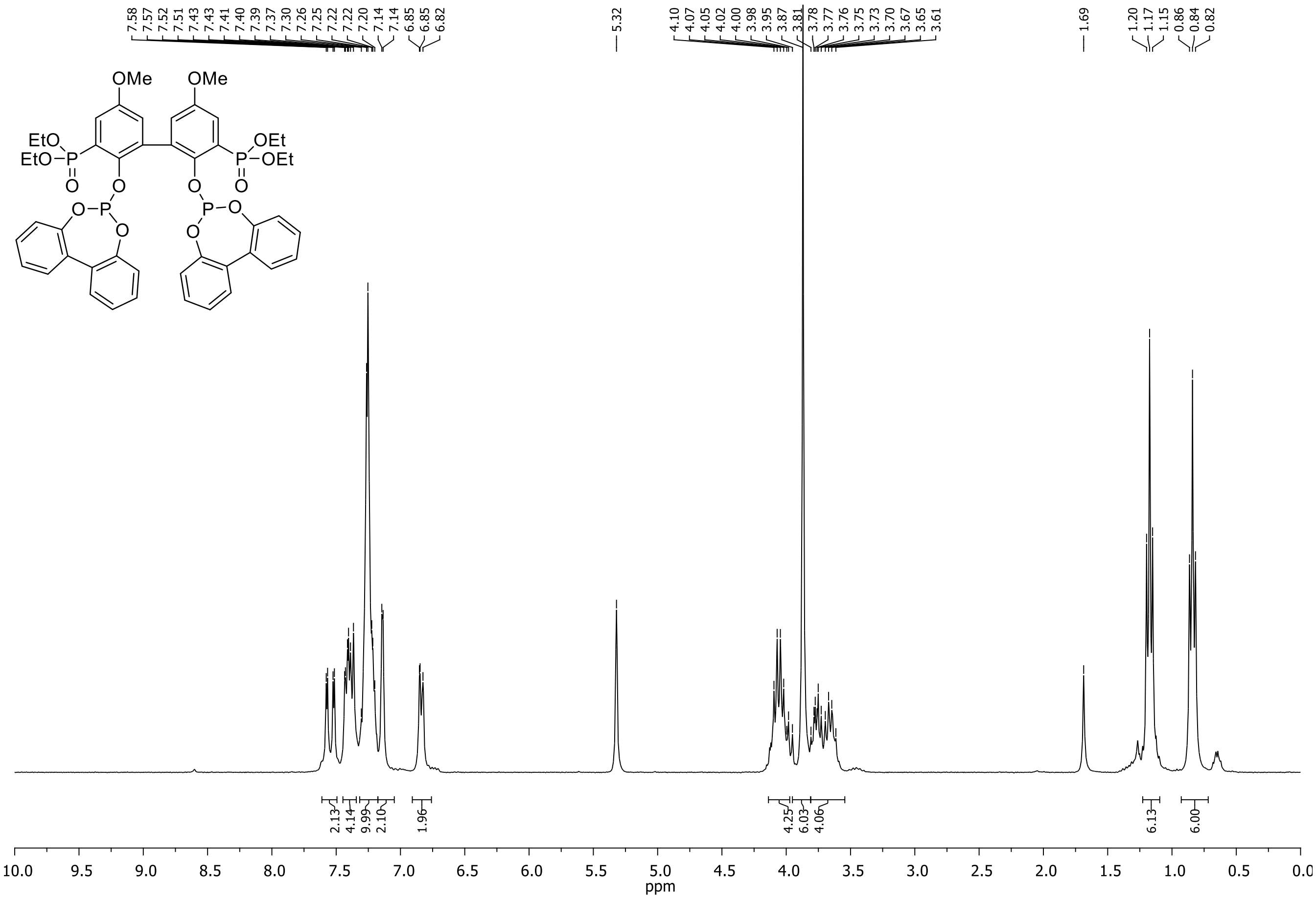

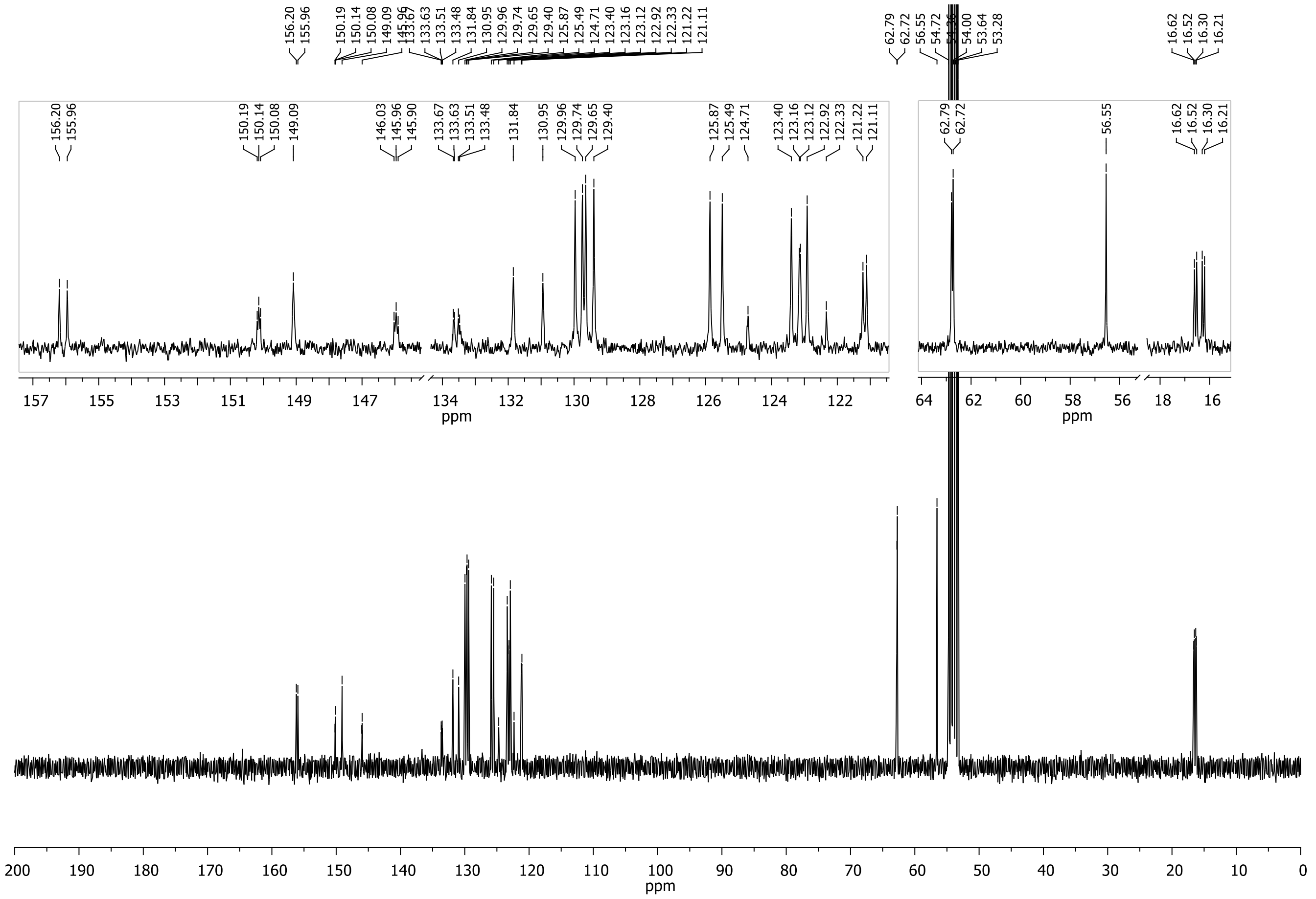

Compound **L2**: ^{1}H-NMR (300 MHz, CD_2Cl_2); ^{13}C-NMR: (76 MHz, CD_2Cl_2)

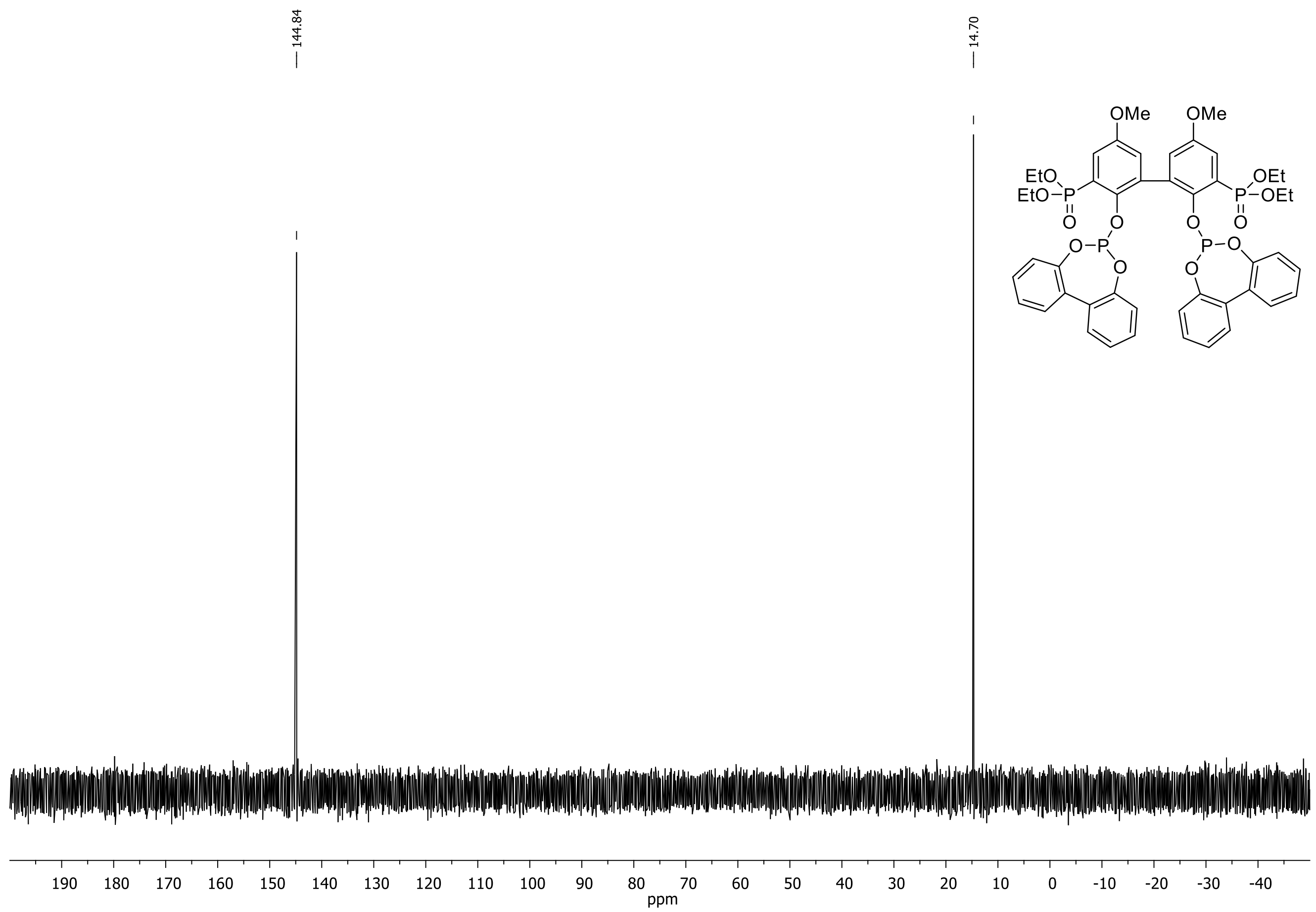

Compound **L2**: ^{31}P-NMR (202 MHz, CD_2Cl_2)

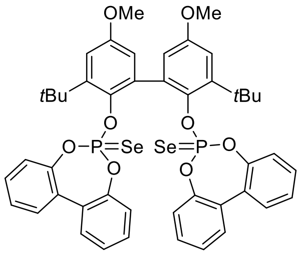

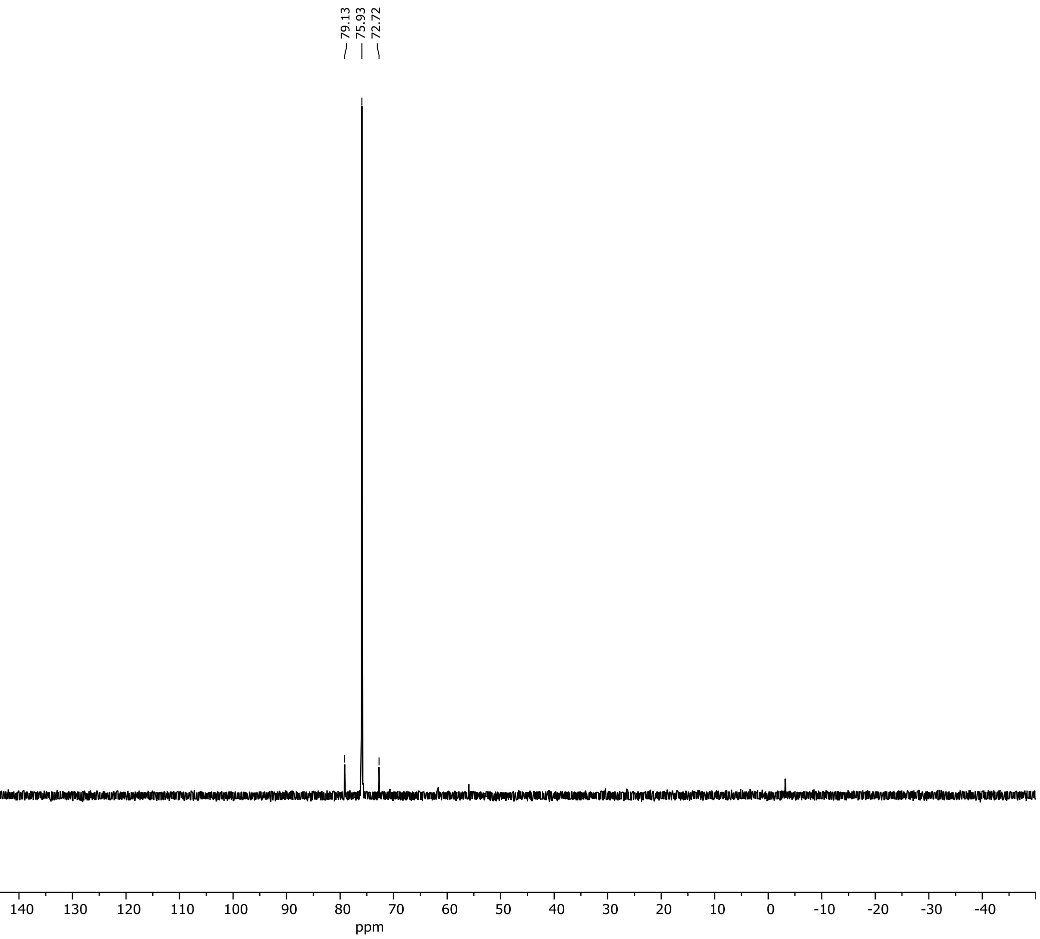

BIPHEPHOS-Se_2: ^{31}P-NMR (162 MHz, DMSO-d_6)

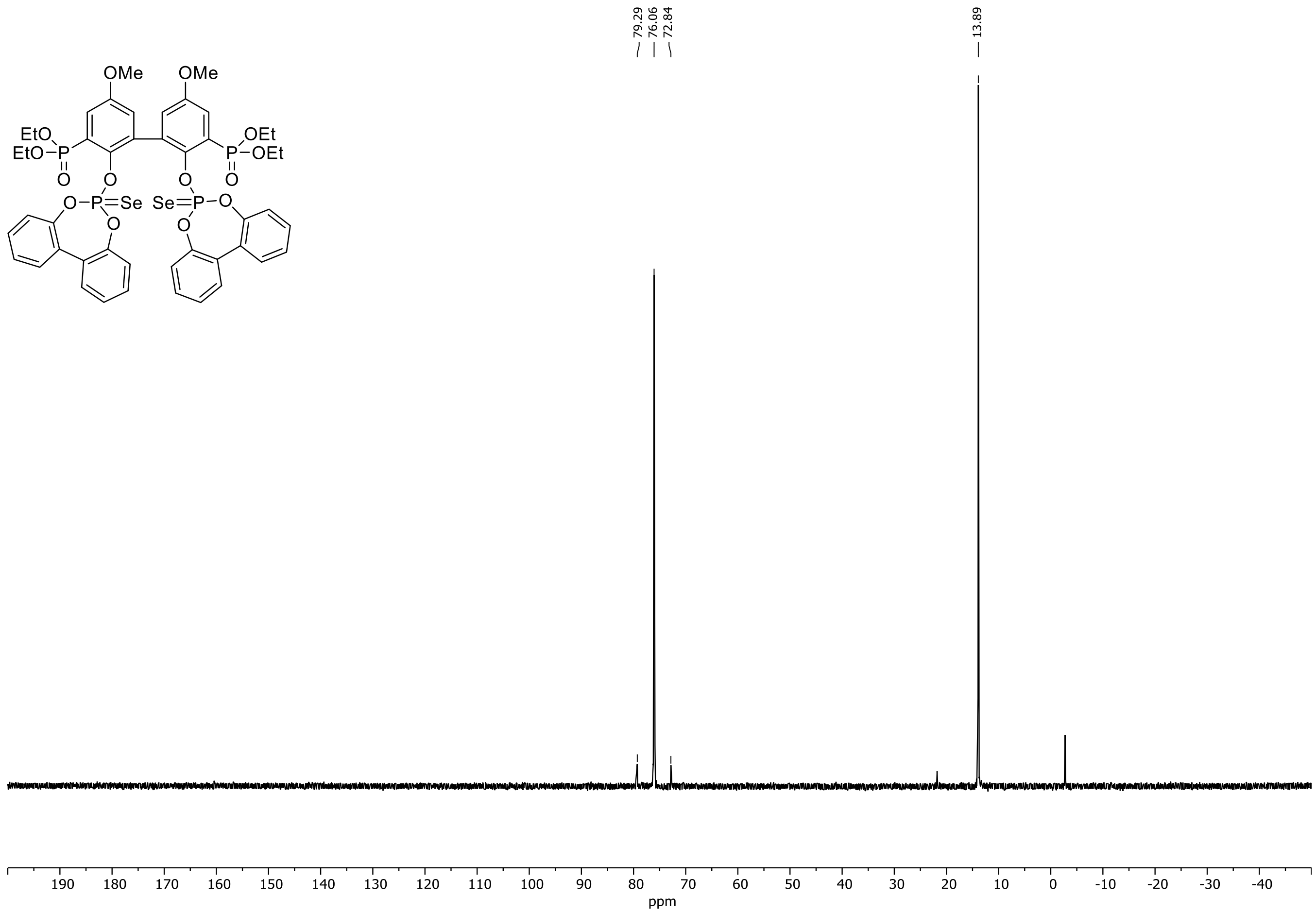

Compound **L2**-Se$_2$: ^{31}P-NMR (162 MHz, DMSO-d_6)

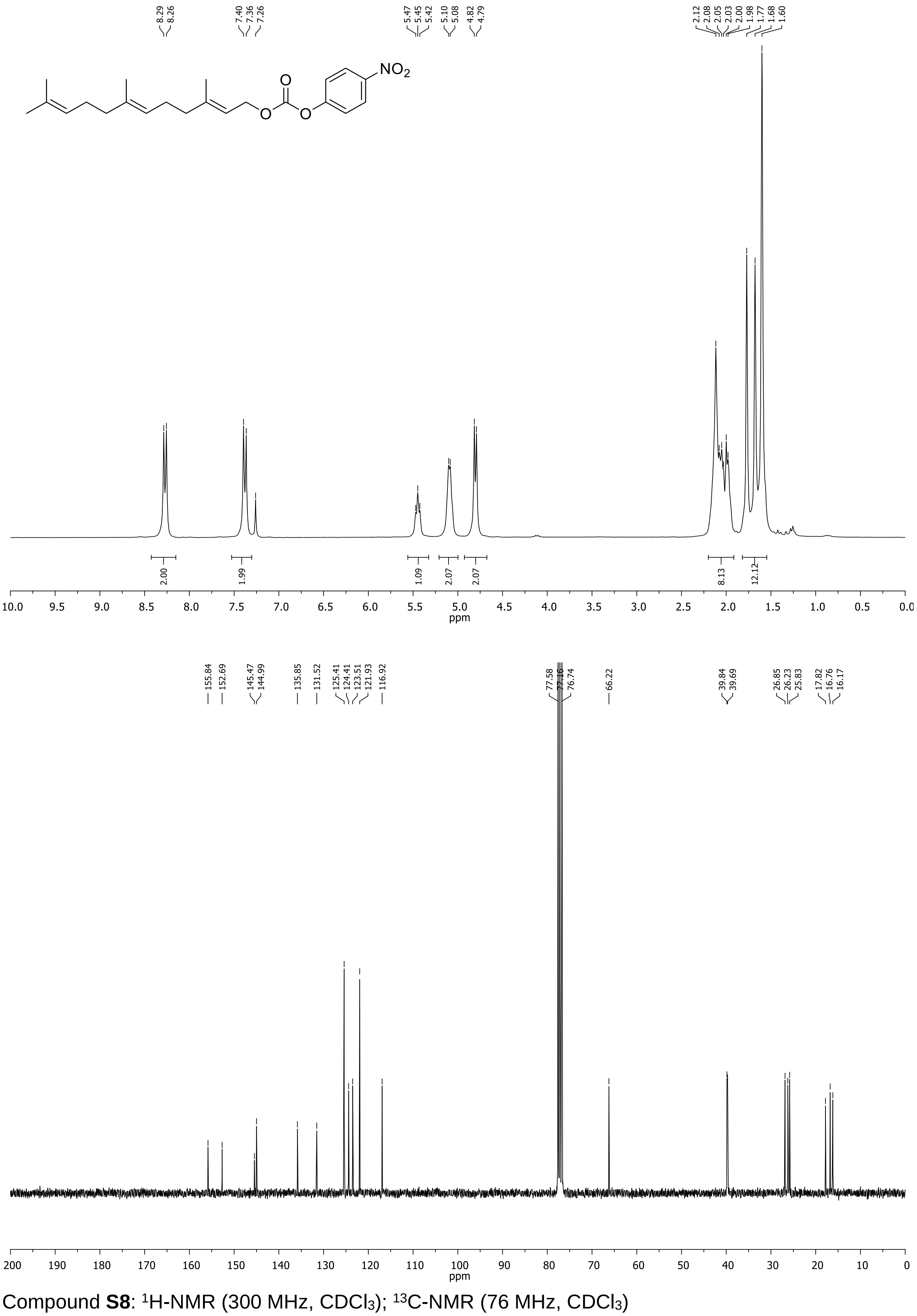

Compound **S8**: ^{1}H-NMR (300 MHz, $CDCl_3$); ^{13}C-NMR (76 MHz, $CDCl_3$)

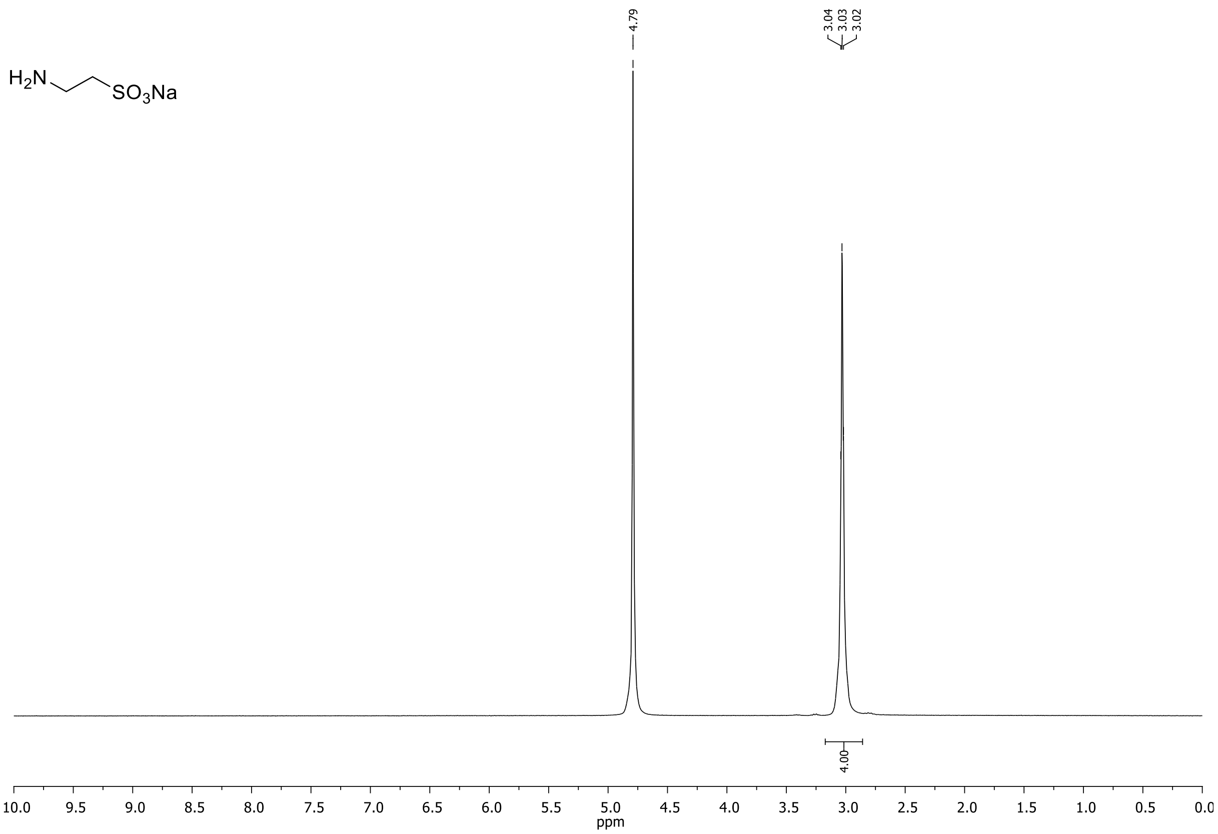

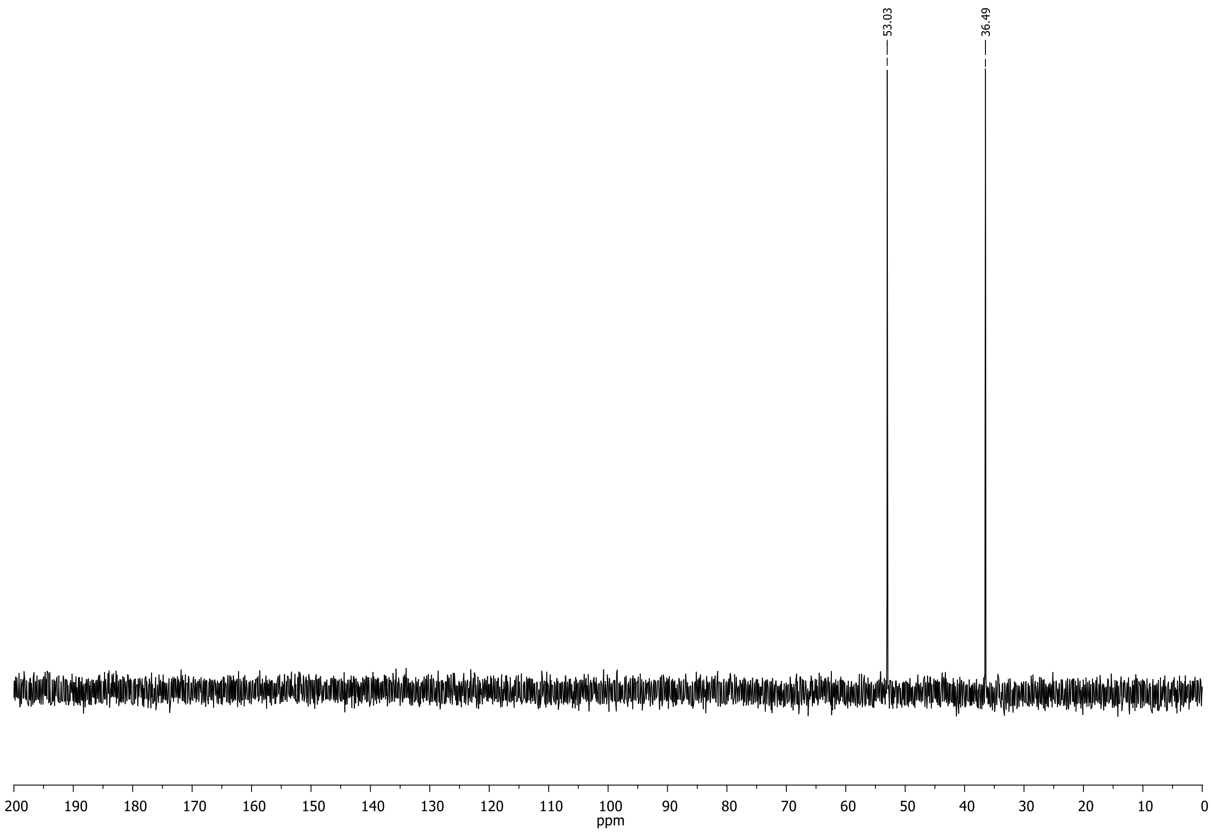

Compound **S9**: ^{1}H-NMR (300 MHz, D_2O); ^{13}C-NMR (76 MHz, D_2O)

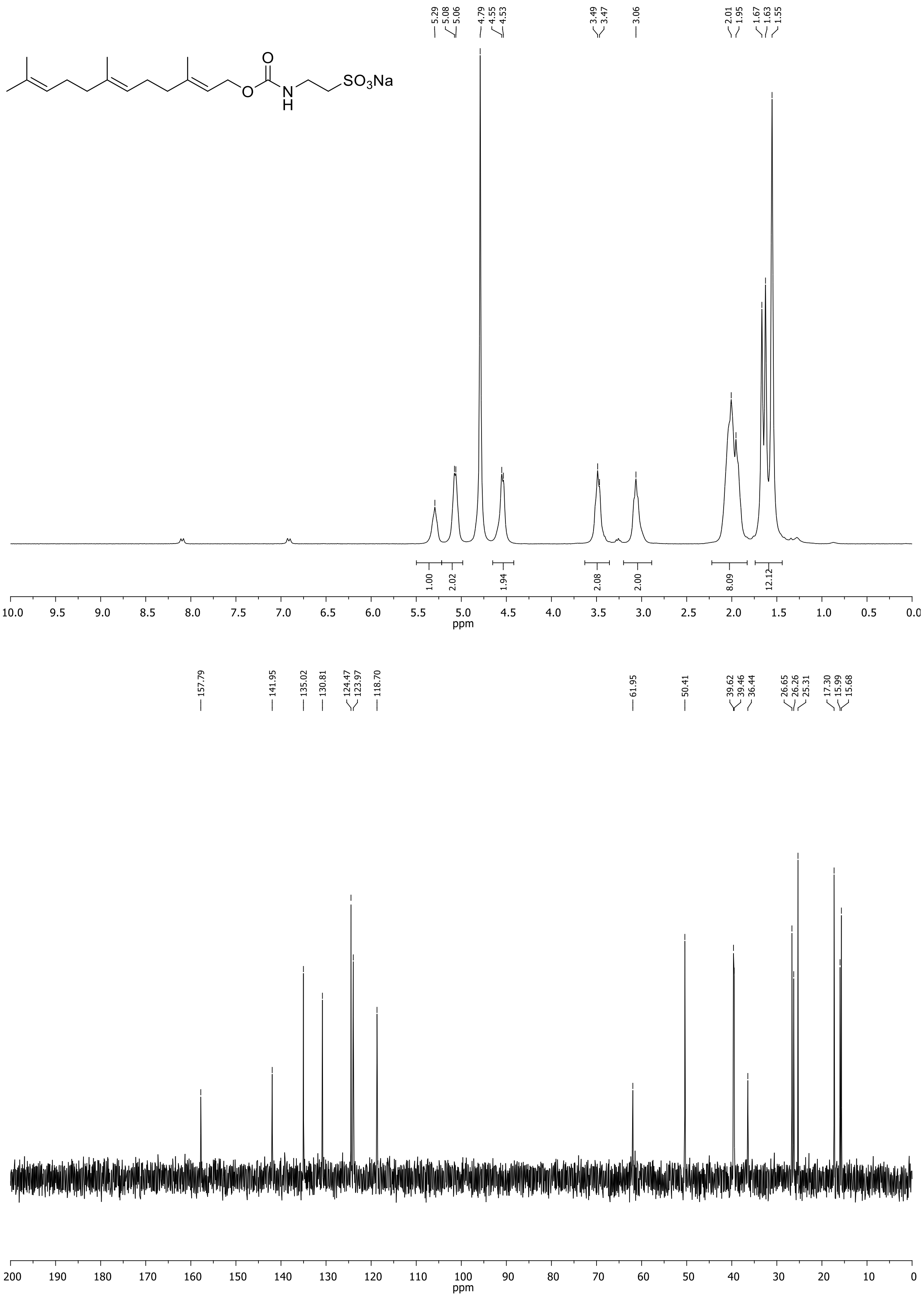

Compound **Rc**: ^{1}H-NMR (300 MHz, D_2O); ^{13}C-NMR (76 MHz, D_2O)
